# A hydroxamic-acid-containing nucleoside inhibits DNA repair nuclease SNM1A†
†Electronic supplementary information (ESI) available: Experimental details for known compounds, NMR spectra of novel compounds, PAMPA experimental details. See DOI: 10.1039/c9ob01133a


**DOI:** 10.1039/c9ob01133a

**Published:** 2019-08-05

**Authors:** William Doherty, Eva-Maria Dürr, Hannah T. Baddock, Sook Y. Lee, Peter J. McHugh, Tom Brown, Mathias O. Senge, Eoin M. Scanlan, Joanna F. McGouran

**Affiliations:** a School of Chemistry and Trinity Biomedical Sciences Institute , Trinity College Dublin , 152-160 Pearse St , Dublin 2 , Ireland . Email: jmcgoura@tcd.ie; b Department of Oncology , MRC Weatherall Institute of Molecular Medicine , University of Oxford , John Radcliffe Hospital , Oxford OX3 9DS , UK; c Department of Chemistry , University of Oxford , 12 Mansfield Road , Oxford OX1 3TA , UK; d Molecular Medicine , Trinity Translational Medicine Institute , Trinity Centre for Health Sciences , Trinity College Dublin , The University of Dublin , St James's Hospital , Dublin 8 , Ireland

## Abstract

Nine modified nucleosides, incorporating zinc-binding pharmacophores, have been synthesised and evaluated as inhibitors of the DNA repair nuclease SNM1A. The series included oxyamides, hydroxamic acids, hydroxamates, a hydrazide, a squarate ester and a squaramide. A hydroxamic acid-derived nucleoside inhibited the enzyme, offering a novel approach for potential therapeutic development through the use of rationally designed nucleoside derived inhibitors.

## Abstract 2

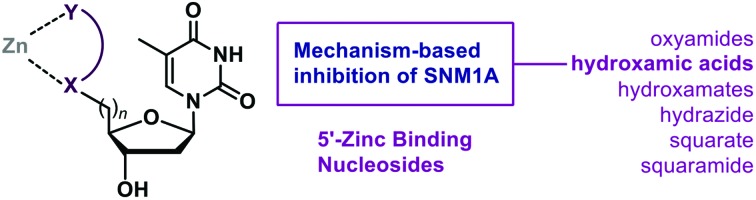
Modified thymidine incorporating zinc-binding pharmacophores offers a novel approach for inhibitor development of SNM1A using rationally designed nucleoside derived inhibitors.

## Introduction

SNM1A is a DNA damage repair enzyme implicated in interstrand crosslink (ICL) repair.^1^ It digests DNA with a 5′-to-3′ polarity past lesions by hydrolysing the phosphodiester backbone of DNA, producing predominantly mononucleotide products.^2^ ICL repair factors are associated with the ageing process,^1^ certain genetic diseases,^3^ and resistance to cancer therapy.^4^ As cells depleted in SNM1A show higher sensitivity to ICLs introduced by several anticancer crosslinking agents,^5^,^6^ SNM1A is a potential therapeutic target for treating cancers that have developed resistance to traditional DNA crosslinking agents. SNM1A interacts with long DNA strands *via* a positively charged patch on the enzyme's surface that binds the negatively charged DNA backbone. This leads to processive activity with higher molecular weight substrates, whereas no processivity is observed with small oligonucleotides.^2^ The 5′-phosphate group of the oligonucleotide substrate binds through H-bonding in the active site and is required for hydrolytic activity. The scissile phosphodiester is postulated to bind to the zinc metal centre prior to attack by an activated water molecule.^2^,^7^ There are a limited number of inhibitors of SNM1A. Among the known inhibitors are cephalosporins^8^ and the metal chelator *o*-phenanthroline.^2^ However, to date, there has not been a modified nucleoside inhibitor.

**Fig. 1:**
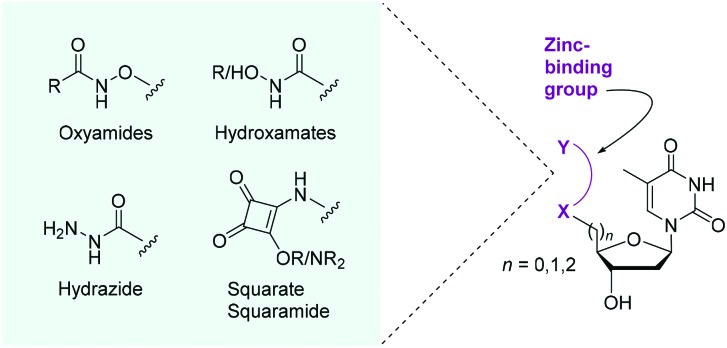
Proposed incorporation of ZBGs at the 5′-position of thymidine for the inhibition of the DNA repair nuclease SNM1A.

We reasoned that the incorporation of compact zinc-binding pharmacophores^9^ at the 5′-position of a nucleoside would have the potential to block the zinc atom-containing active site of SNM1A and thus inhibit the digestion of single strands of DNA. The nucleobase and tetrahydrofuran ring would provide natural substrate recognition for the enzyme while the zinc-binding groups (ZBGs) would serve to both mimic the phosphate group and form a stable chelating interaction with the metal(s) in the active site. We focused on the installation of ZBGs and potential metal binding groups at the 5′-position of thymidine (Fig. 1). We aimed to incorporate the following bidentate chelating moieties: oxyamides, hydroxamic acids, hydroxamates, a hydrazide and a squarate ester/amide. These modifications will serve a dual purpose: both mimicking the 5′-phosphate group and binding the zinc in the active site of SNM1A. Hydroxamic acids are classic ZBGs with their incorporation featured in histone deacetylase inhibitors, for example.^10^ As a renowned metal-binding group^11^ we anticipated that hydroxamic acid derivatives could be good inhibitors of SNM1A. We also explored neutral oxyamide/hydroxamate moieties as potential inhibitors. Squaramides have been demonstrated to chelate metals with the added dimension of being phosphate mimics and thus display excellent precedent for incorporation into nucleoside-based inhibitors.^12^,^13^

In terms of inhibitor design, thymine was selected as the nucleobase as SNM1A has no reported sequence selectivity and the absence of a primary amine group in thymine allows for ease of synthesis and avoids protecting group manipulations on this moiety. The ZBG-modified nucleosides were tested as competitive inhibitors against oligonucleotide strands (20–21 nucleotides in length) which represents a natural substrate for SNM1A in assays with plate reader and gel electrophoresis readouts.

## Results and discussion

**Scheme 1:**
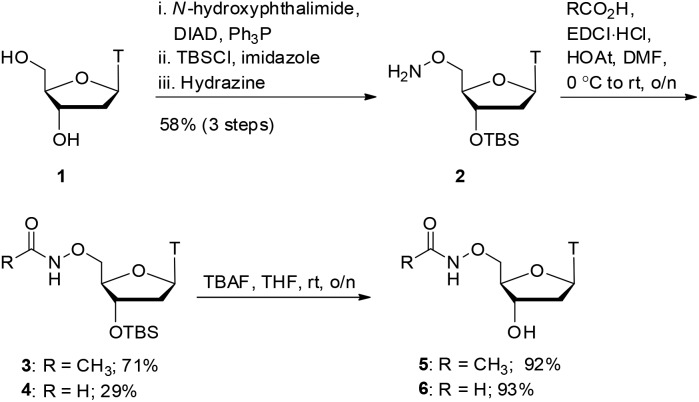
Acetyl and formyl oxyamide synthesis. T = thymine.

As shown in Scheme 1, we focused our initial efforts on the synthesis of oxyamide-derived thymidine compounds. Using literature conditions,^14^ commercially available thymidine **1** was reacted with *N*-hydroxyphthalimide in a Mitsunobu reaction, followed by 3′-OH protection as a silyl ether and subsequent hydrazinolysis to give the oxyamine product **2** in 58% yield over 3 steps. This material was coupled with acetic acid and formic acid in 71% and 29% yield respectively using EDCI/HOAt-mediated coupling.^15^ Deprotection of the silyl ethers **3** and **4** using TBAF gave the hydroxy compounds **5** and **6** in excellent yields (92% and 93% respectively).

**Scheme 2:**
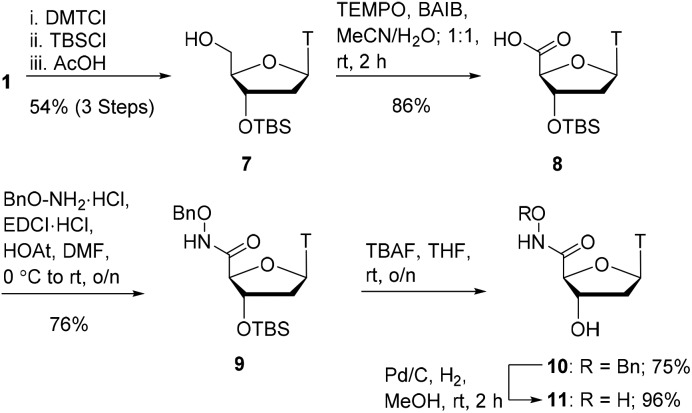
Hydroxamic acid synthesis.

With oxyamides **5** and **6** in hand, we then turned our attention to the synthesis of hydroxamic acid-derived nucleosides (Scheme 2). Using literature conditions, thymidine **1** was converted into the 3′-silyl ether **7** in 54% yield over 3 steps. This was achieved by 5′-OH protection of thymidine **1** with a dimethoxytrityl group,^16^ 3′-OH protection as a silyl ether,^17^ and finally dimethoxytrityl deprotection.^18^ A TEMPO-BAIB-mediated oxidation in acetonitrile/water provided carboxylic acid^19^**8** which would later serve as a point of divergence in our syntheses. Coupling of carboxylic acid **8** with benzyloxyamine under EDCI/HOAt conditions provided the hydroxamate **9** in 76% yield. TBS deprotection was achieved using TBAF in tetrahydrofuran which gave the hydroxy compound **10** in 75% yield. The benzyl protecting group was removed *via* catalytic hydrogenation (Pd/C–H_2_) to give hydroxamic acid **11** in 96% yield.

**Scheme 3:**
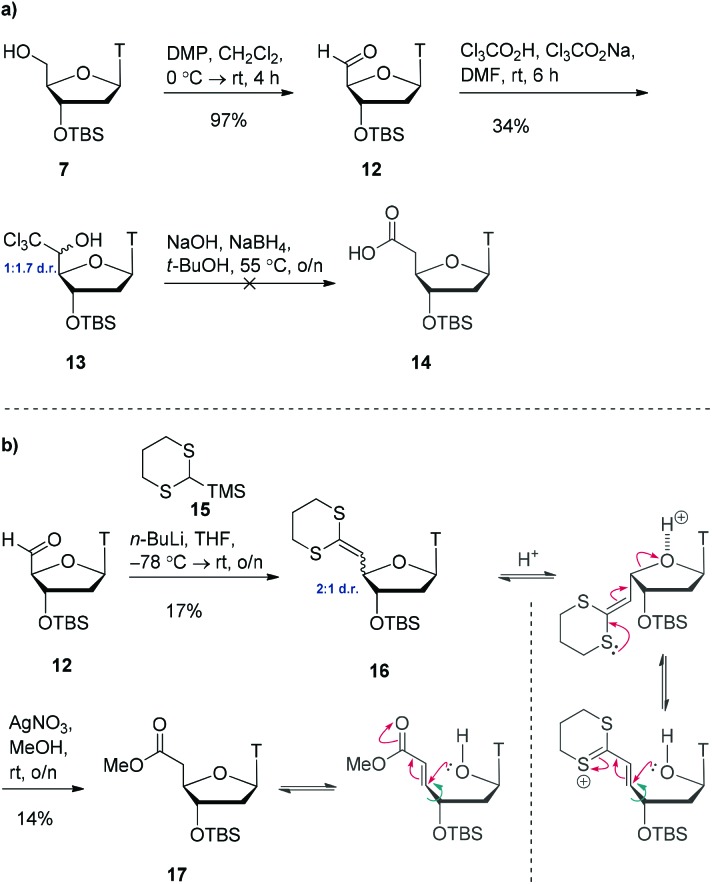
(a) Attempted one-carbon homologation *via* trichloromethylcarbinol **13**. (b) One-carbon homologation *via* Peterson olefination and postulated epimerisation mechanisms.

We postulated that modifying the length of the carbon chain attached to the hydroxamic acid would provide a valuable structure–activity-relationship. We therefore pursued 1- and 2-carbon homologues of compound **11** as shown in Scheme 3a. Initial attempts to oxidise alcohol **7** using TEMPO/BAIB in anhydrous dichloromethane failed to yield any aldehyde **12** but instead yielded multiple decomposition products. However, using Dess–Martin periodinane,^20^ we were able to access the aldehyde **12** in almost quantitative yield. Using Snowden's conditions,^21^ treatment of this freshly prepared aldehyde **12** with trichloroacetic acid and sodium trichloroacetate provided the trichloromethyl carbinol **13** as a 1 : 1.7 mixture of 5′-epimers in 34% yield. Treatment of this material with sodium borohydride and sodium hydroxide to access carboxylic acid **14** resulted in decomposition. After multiple unsuccessful attempts at optimising this reaction, we opted for a different approach.

Starting from aldehyde **12**, a Peterson olefination with commercially available silane **15** gave the ketene-dithioacetal **16** in low yield (Scheme 3b). This compound was found to be highly acid sensitive and as such it was isolated as a 2 : 1 mixture of 4′-epimers. This epimerisation event occurred during column chromatography as analysis of the crude reaction mixture by ^1^H NMR spectroscopy indicated the presence of one diastereoisomer. Attempts at purification on alumina resulted in decomposition. Due to the acid sensitivity of this molecule, it was used immediately in the next step (carried forward as a mixture of 4′-epimers). We initially attempted to convert the ketene-dithioacetal **16** into the corresponding methyl ester **17** using CuSO_4_·5H_2_O in refluxing methanol,^22^ however this resulted in decomposition of the starting material. Switching to AgNO_3_ in refluxing methanol^23^ for one hour (or rt overnight) did provide product **17**, but in very low yield (14%). Interestingly, analysis of the ^1^H NMR spectrum of the complex crude reaction mixture showed a virtually 1 : 1 mixture of 4′-epimers. Remarkably, after column chromatography on silica gel, only one epimer was isolated. We speculate that upon exposure of this molecule to silica gel, a β-elimination-type reaction could be occurring, delivering only one epimer (determined by NOE). However, given the poor yields for this sequence, we do not view this route as viable to access the 1-carbon homologue of compound **11**.

**Scheme 4:**
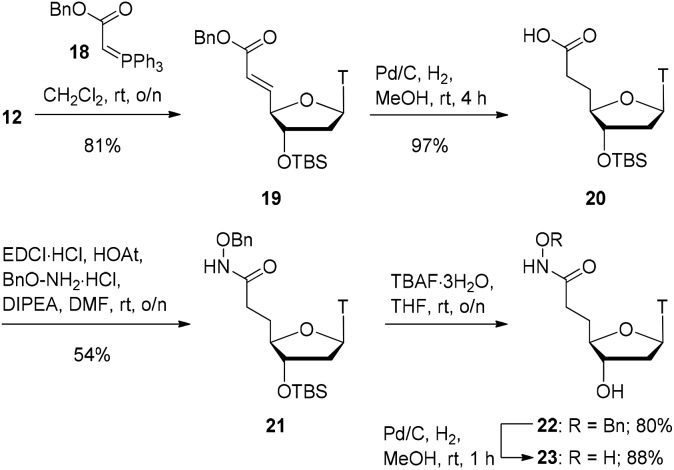
Synthesis of two-carbon homologue **23**.

At this point, we turned our focus to the 2-carbon homologation (Scheme 4). Starting from aldehyde **12**, using adapted literature conditions,^24^ a Wittig reaction with stablised ylide **18** gave the α,β-unsaturated benzyl ester **19** in 81% yield. Catalytic hydrogenation of this material with Pd/C delivered the global hydrogenation product **20** (saturated carboxylic acid) in virtually quantitative yield. EDCI-mediated coupling of carboxylic acid **20** with benzyloxyamine provided the hydroxamate product **21** in 54% yield. The silyl ether in compound **21** was removed with TBAF to provide alcohol **22** in 80% yield. Finally, hydrogenolysis of the benzyl group with Pd/C–H_2_ furnished the final hydroxamic acid product **23** in 88% yield.

**Scheme 5:**
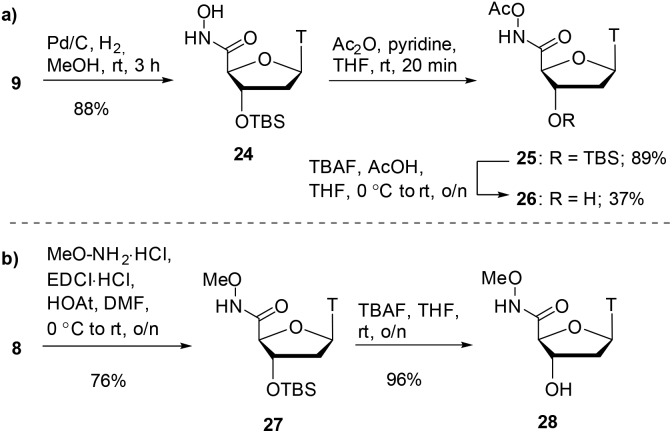
a) Acetate-capped hydroxamic acid synthesis. (b) Methyl-capped hydroxamic acid synthesis.

Alongside hydroxamic acids **11** and **23**, we explored the idea of capping the hydroxyl portion of the hydroxamic acid with labile and non-labile groups. It was postulated that the labile acetate group on the hydroxamic could be cleaved in buffer during the assay and could have pro-drug potential. In order to discern the importance the free hydroxy group has in the inhibition of SNM1A, we also pursued an *O*-methylated hydroxamic acid. As shown in Scheme 5a, hydroxamate **9** was reduced by catalytic hydrogenation which gave hydroxamic acid **24** in 88% yield. Acetylation of compound **24** with acetic anhydride capped the hydroxamic acid moiety as an acetate giving compound **25** in 89% yield. Achieving high yields for the silyl deprotection of this compound proved to be a considerable challenge.

Optimisation was required for this transformation as use of TBAF alone did not sufficiently deprotect the silyl ether. The addition of acetic acid improved the yields of alcohol **26** to 37% yield. As shown in Scheme 5b, the final hydroxamate in the series was a methyl-capped hydroxamic acid. We performed a coupling reaction of carboxylic acid **8** with methoxyamine which gave the methoxyamide product **27** in 76% yield. Deprotection of the silyl ether with TBAF proceeded smoothly to give the alcohol compound **28** in 96% yield.

**Scheme 6:**
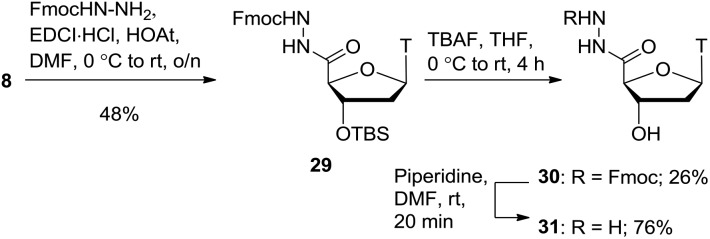
Hydrazide synthesis.

Further to the hydroxamic acid and hydroxamate series, the hydrazide moiety is also known to be a potent zinc binder.^9^ As shown in Scheme 6, coupling of carboxylic acid **8** with Fmoc-protected hydrazine (see ESI† for synthesis) gave the protected hydrazide product **29** in 48% yield. Deprotection of the 3′-silyl ether proved to be a difficult transformation and a range of conditions were explored including various acids, TBAF and a combination of TBAF and acid. Ultimately, the use of TBAF with acetic acid gave the alcohol product **30** in a modest 26% yield. The Fmoc group was removed with piperidine to afford the free hydrazide product **31** in 76% yield.

The squaramide functionality is a known phosphate bioisostere in nucleoside chemistry^12^,^13^ and has also found other varied uses in medicinal chemistry.^25^ Furthermore, hydroxylamine derived squaramides have been demonstrated to possess metal and zinc-binding capabilities.^26^–^29^

**Scheme 7:**
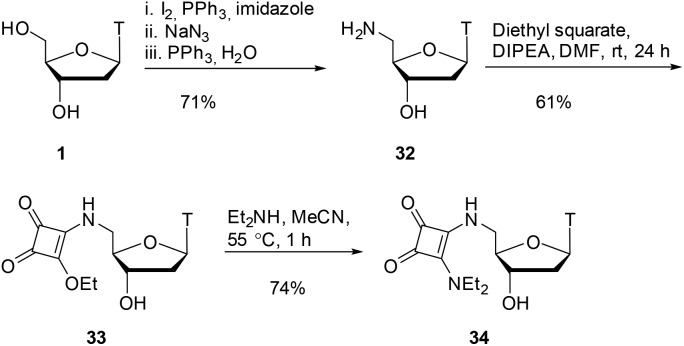
Squarate ester and squaramide synthesis.

As shown in Scheme 7, starting from thymidine **1**, using literature conditions, we converted the 5′-OH group into an amino group through an iodination/displacement/reduction sequence in 71% yield over 3 steps.^30^–^32^ Using literature conditions,^13^ coupling of amine **32** with diethyl squarate under basic conditions provided the squarate ester **33** in 61% yield. This material was subsequently converted into the squaramide **34** in 74% yield.

**Fig. 2:**
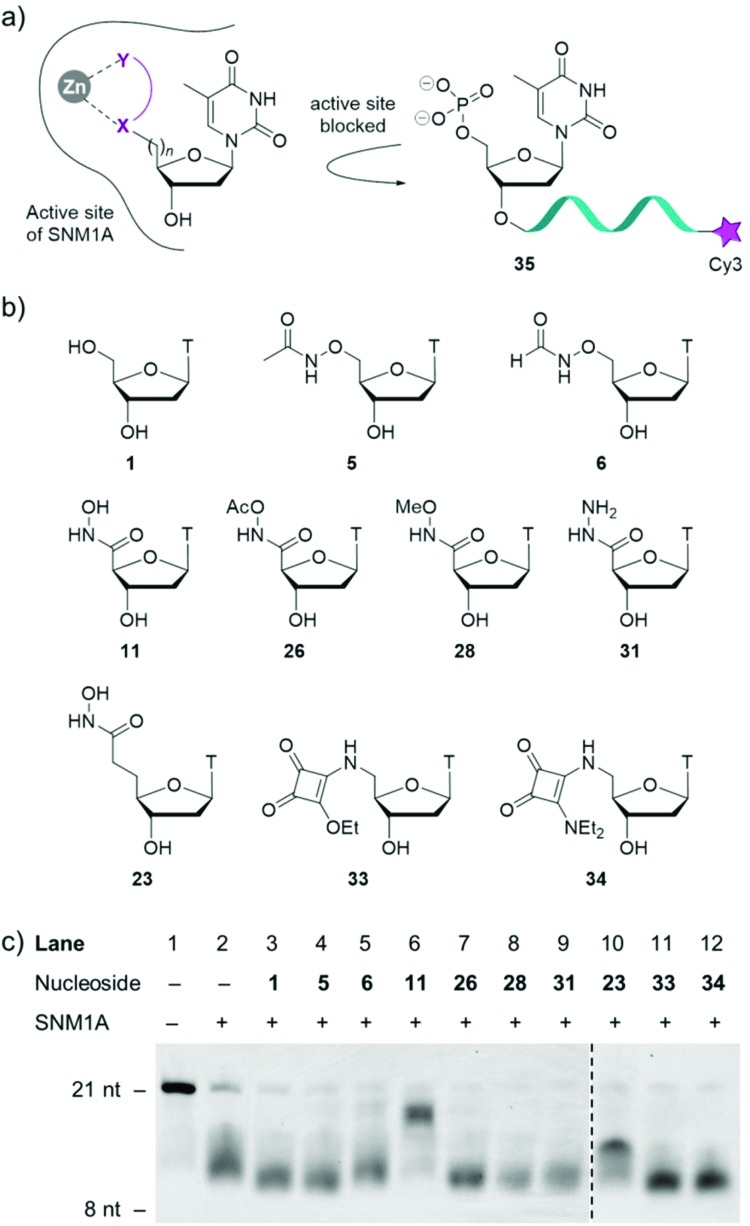
Biological evaluation of modified nucleosides **5**, **6**, **11**, **23**, **26**, **28**, **31**, **33** and **34** as competitive inhibitors of SNM1A. (a) Postulated inhibition mechanism. (b) Modified nucleosides evaluated as inhibitors of SNM1A. (c) Denaturing PAGE gel of the extent of digestion of oligonucleotide **35**. SNM1A was pre-incubated with inhibitors for 5 minutes at 1 mM concentrations. Oligonucleotide **35** was then added and the assay was halted after one hour. nt = nucleotides.

With a diverse range of 5′-modified nucleosides in hand, their ability to inhibit the human exonuclease SNM1A was evaluated (Fig. 2). As SNM1A has been documented to efficiently digest single-stranded DNA (ssDNA) substrates, we synthesised a ssDNA oligonucleotide containing the Cy3 fluorophore at the 3′-end of the oligonucleotide (compound **35**). This compound served as a substrate for the enzyme and could be used to monitor the activity of our inhibitor series by gel electrophoresis. SNM1A was incubated with inhibitors **5**, **6**, **11**, **23**, **26**, **28**, **31**, **33** and **34**, respectively at 1 mM concentrations for 5 minutes at 37 °C. After inhibitor pre-incubation, oligonucleotide strand **35** was introduced to the reactions to compete with the inhibitors. After 1 hour, the assay was stopped and the extent of digestion of the oligonucleotide **35** was analysed by denaturing gel electrophoresis. As a control, thymidine **1** was included in the assay to ensure any inhibitory effect could be attributed to the modifications on the nucleoside rather than simply non-covalent interaction between the enzyme and the thymine nucleobase and the tetrahydrofuran ring.

As shown in Fig. 2c, the results (acquired in duplicate) demonstrate that hydroxamic acids **11** and **23** act as inhibitors of SNM1A as the oligonucleotide is not fully digested in this assay. The difference in digestion of oligonucleotide **35** in the presence of the two hydroxamic acids **11** and **23** highlights the importance of the position of the hydroxamic acid group relative to the nucleoside. In the presence of compound **11** the oligonucleotide products obtained are only slightly shorter than the substrate **35**, indicating that hydroxamic acid **11** competes with the 21-nucleotide substrate. The reaction yields shorter fragments in the presence of nucleoside **23**, which implies that compound **23** is unable to compete with the 21 mer **35** or the longer oligonucleotides that result from the initial hydrolysis events. However, compound **23** inhibits the hydrolysis of already shortened oligonucleotides, which benefit less from distal binding sites of SNM1A.

All the other inhibitors (**5**, **6**, **26**, **28**, **31**, **33** and **34**) in this assay show virtually no inhibition of SNM1A. Formyloxyamide **6** does show marginal levels of enzyme inhibition but given the relatively weak inhibitory potential of this compound, we opted not to pursue this for further analysis.

Acetamide derivative **5** displays no inhibition and both labile and non-labile capped hydroxamate **26** and **28**, respectively show no inhibition, indicating the crucial role the free hydroxy group has in enzyme inhibition. Further corroborating evidence for this is that replacing the OH with NH_2_ (*i.e.* hydrazide **31**) results in no inhibition of SNM1A. Squarate ester **33** and squaramide **34** display no activity in this assay.

**Fig. 3:**
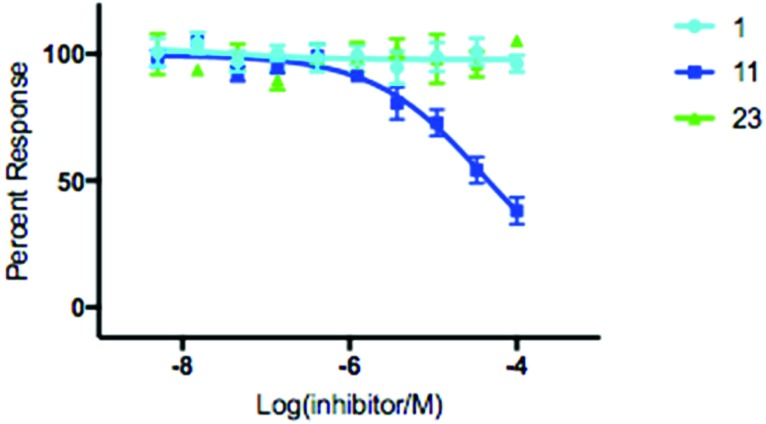
IC_50_ determination of modified nucleoside **11** with SNM1A. Error bars generated from 4 independent repeats.

With hydroxamic acids **11** & **23** showing the most initial promise, we opted to further investigate the extent to which it could inhibit SNM1A through determination of IC_50_ values (Fig. 3). Thymidine **1** was included as a control for this experiment, and for inhibitors **11** & **23** IC_50_ determination was carried out using a real time fluorescence assay in a manner analogous to Lee *et al.* with a determined IC_50_ of 139 μM for compound **11** with no discernible inhibition of SNM1A by either control compound **1** or hydroxamic acid **23** (Fig. 3).^8^ To evaluate the membrane permeability of compound **11**, a parallel artificial membrane permeability assay (PAMPA) was performed.^33^ Nucleoside **11** was unable to cross the artificial membrane (see ESI†), presumably due to the hydrophilic nature of the hydroxamic acid moiety. A pro-drug approach where the active moiety is masked by a labile protecting group, as envisaged for compound **26**, could be used to overcome this limitation. As evident by the lack of activity of compound **26** (Fig. 2c, lane 7), the nature of the protecting group is crucial to the successful release of the active compound. The results of the permeability assay indicate that a significant increase in lipophilicity is required for the successful diffusion of derivatives of compound **11** across a membrane.

**Fig. 4:**
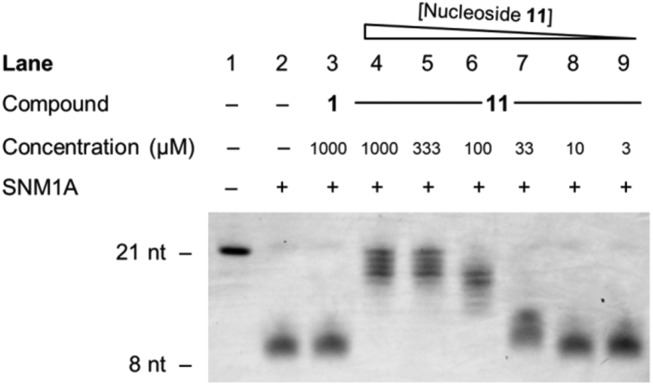
Denaturing PAGE electrophoresis gel of the extent of digestion of oligonucleotide **35** with decreasing concentrations of hydroxamic acid **11** (1 mM to 3 μM). SNM1A was pre-incubated with inhibitors for 5 minutes. Oligonucleotide **35** was then added and the assay was halted after one hour. nt = nucleotides.

Finally, inhibitor **11** was examined in a gel-based assay across concentrations from 1 mM to 3 μM, with thymidine **1** (1 mM) included as a control. Hydroxamic acid **11** was incubated with SNM1A for 5 minutes at 37 °C, in decreasing concentrations, after which time oligonucleotide **35** was added to each reaction. After one hour, the reaction mixture was halted and the extent of digestion of the oligonucleotide was analysed by gel electrophoresis. This experiment was run in duplicate. As can be seen from Fig. 4, hydroxamic acid **11** is an effective inhibitor of SNM1A at higher concentrations (1 mM–0.1 mM) and has a marginal effect as low as 33 μM.

## Conclusions

In summary, we have synthesised a range of zinc-binding pharmacophores incorporated at the 5′-position of thymidine and these were tested against the human exonuclease SNM1A for inhibition. The hydroxamic acid-derived nucleoside **11** proved to be a novel competitive inhibitor of SNM1A with an IC_50_ of 139 μM and is the first nucleoside-based inhibitor of the exonuclease. The free hydroxy portion of the hydroxamic acid group is essential to activity, as capped or protected hydroxamates **26** and **28** are not active in this assay. Furthermore, the proximity of the hydroxamic acid group to the nucleoside ring proved to be crucial as hydroxamic acid **23** showed significantly less inhibition of SNM1A. Surprisingly, the hydrazide containing nucleoside **31** showed no activity in this assay, highlighting the crucial role the oxygen of the hydroxamic acid plays in SNM1A inhibition. It is proposed that the mechanism of inhibition of this enzyme is *via* the occupation of the active site and complexation of the zinc ion(s). Hydroxamic acid **11** serves as a lead compound and further optimisation such as utilising other nucleobases or labile protecting groups could improve the activity and membrane permeability of this molecule. Compound **11** could find application in a combination therapy for cancers which are resistant to interstrand cross-linking agents and is currently under investigation in our laboratory.

## Experimental

### Biological evaluation

#### Inhibition assay with SNM1A

SNM1A was stored as a 1.0 μM solution in reaction buffer (20 mM HEPES–KOH, pH 7.5, 50 mM KCl, 10 mM MgCl_2_, 0.05% Triton-X, 0.1 mg mL^–1^ BSA, 5% glycerol, 0.5 mM DTT). Thymidine **1** (control), and modified nucleosides **5**, **6**, **11**, **23**, **26**, **28**, **31**, **33** and **34** (1 mM) were treated with SNM1A (698–1040) (25 fmol) in reaction buffer containing 4% DMSO (10 μL) on ice and incubated at 37 °C for 5 minutes prior to the addition of the fluorescent oligonucleotide substrate **35** (0.8 pmol) and further incubation at 37 °C for a further 60 minutes. The reaction was stopped by the addition of stop solution (2 μL, 95% formamide, 10 mM EDTA) followed by heating to 95 °C for 3 minutes. Digested oligonucleotides were separated on a 15% acrylamide 6.5 M urea gel [2.9 g urea, 2.7 mL 40% acrylamide–bisacrylamide 25 : 1, 1.4 mL 5× TBE (0.45 M Tris, 0.45 M boric acid, 0.01 M EDTA pH 8.0), 0.6 mL H_2_O] in 1× TBE at 150 V for 100 minutes alongside bromophenol blue and xylene cyanol as markers for **8** nucleotides and **28** nucleotides respectively and imaged using Typhoon FLA 9500.

#### Inhibition assay with SNM1A (decreasing concentrations of hydroxamic acid **11**)

In a procedure identical to the above inhibition assay, hydroxamic acid **11** was incubated with SNM1A in the following concentrations: 1 mM, 333 μM, 100 μM, 33 μM, 10 μM and 3 μM. Thymidine **1** (1 mM) was incubated with SNM1A as a control. Digested oligonucleotides were separated on a 15% acrylamide 6.5 M urea gel in 1× TBE at 150 V for 90 minutes alongside bromophenol blue and xylene cyanol as markers for 8 nucleotides and 28 nucleotides respectively and imaged using Typhoon FLA 9500.

#### Experimental protocol for real-time fluorescence assay

Real-time fluorescence assays were performed as previously described^8^ utilising a 20-nucleotide ssDNA substrate, modified to contain a black-hole quench (BHQ) moiety on the 5′ nucleotide, and a fluorescein-conjugated T, eight nucleotides away (Eurofins Genomics). Reactions were carried out in black 384-well microplates in a total volume of 25 μL in nuclease buffer (20 mM HEPES–KOH, pH 7.5, 50 mM KCl, 10 mM MgCl_2_, 0.5 mM DTT, 0.05% (v/v) Triton-X100, 5% (v/v) glycerol), 25 nM DNA substrate, 0.25 nM SNM1A, with increasing concentrations (0–1000 μM) of each inhibitor. SNM1A was incubated with the inhibitor in the above buffer for 10 minutes at rt, before the reaction was started by the addition of DNA substrate. The fluorescence spectra were measured using a PHERAstar FSX (BMG Labtech) (excitation at 495 nm, emission at 525 nm) with 15 readings taken every 150 seconds. The fluorescence intensity for each reaction was plotted against time, and the rate of increase was determined, normalised to the 0 inhibitor control, and plotted against compound concentration. This was fitted to a log[inhibitor]–response curve on Prism software (GraphPad Software, Inc., La Jolla, CA, USA) to calculate the IC_50_ values.

#### General experimental methods

Reagents were obtained from commercial suppliers and were used without further purification. CH_2_Cl_2_ and THF were dried using a PureSolv MD solvent purification system. Petroleum ether (PE) refers to the fraction that boils at 40–60 °C. Oxygen free, anhydrous argon was obtained from BOC gases. Flash column chromatography was performed using flash silica 60 Å (230–400 mesh). Thin-layer chromatography was performed on silica coated aluminium sheets (60 F_254_). Compounds were visualised with UV light and aqueous potassium permanganate stain (3 g KMnO_4_, 20 g K_2_CO_3_, 300 mL H_2_O) followed by heating. Melting points were recorded on a Griffin melting point apparatus and are uncorrected. Infrared (IR) spectra were recorded on a PerkinElmer spectrophotometer. ^1^H and ^13^C NMR spectra were recorded on Bruker 600 MHz and 400 MHz system spectrometers in CDCl_3_, DMSO-*d*_6_ or CD_3_OD. Chemical shifts are quoted in parts per million (ppm) relative to the residual protonated solvent. Coupling constants (*J*) are quoted in hertz accurate to 0.2 Hz. Chemical shift assignments are based on two-dimensional NMR experiments including TOCSY, HSQC and HMBC. High resolution mass spectra were carried out on a Bruker ESI or APCI HRMS analyser. Modified nucleosides are numbered according to standard nucleoside convention.

### Synthetic procedures

#### *N*-Formyl-5′-*O*-amino-3′-*O*-(*tert*-butyldimethylsilyl)thymidine **4**

Under argon, formic acid (126 μL, 3.34 mmol) was dissolved in anhydrous DMF (13 mL), cooled to 0 °C and stirred. HOAt (480 mg, 3.53 mmol) and EDCI·HCl (666 mg, 3.47 mmol) were added and the reaction mixture was stirred for 10 minutes. Oxyamine **2** (950 mg, 2.56 mmol) was added and the reaction mixture was warmed to rt and stirred for 24 hours. After this time, TLC analysis (CH_2_Cl_2_/MeOH; 19 : 1) showed the consumption of starting material (*R*_f_ = 0.5) and the formation of product (*R*_f_ = 0.1). The reaction mixture was diluted with EtOAc (100 mL) and washed with sat. aq. NaHCO_3_ solution (100 mL). The aqueous layer was extracted with EtOAc (2 × 100 mL) and the combined organic extracts were washed with brine (100 mL), dried over Na_2_SO_4_, filtered and solvent was removed *in vacuo* to give the crude product. Purification by column chromatography (CH_2_Cl_2_/MeOH; 98 : 2) gave formyloxyamide **4** as a white foam (300 mg, 29%). IR *ν*_max_ 3198, 2955, 2928, 2856, 1690, 1470, 1370, 1276, 1254, 1199, 1135, 1086, 1032, 952, 836, 780 cm^–1^. ^1^H NMR (400 MHz, CDCl_3_): *δ* = 0.09 (s, 6H, 2 × CH_3_^TBS^), 0.89 (s, 9H, *t*-Bu^TBS^), 1.94 (s, 3H, CH_3_^T^), 2.23–2.33 (m, 2H, H-2′a + H-2′b), 4.01–4.02 (m, 1H, H-4′), 4.13 (dd, *J* = 10.6, 3.3 Hz, 1H, H-5′a), 4.21 (dd, *J* = 10.6, 2.1 Hz, 1H, H-5′b), 4.56–4.63 (m, 1H, H-3′), 6.13 (app. t, *J* = 7.1 Hz, 1H, H-1′), 7.40 (s, 1H, H-6), 7.99 (s, 1H, CHO), 8.32 (s, 1H, O–NH), 8.76 (s, 1H, NH-3) ppm. ^13^C NMR (100 MHz, CDCl_3_): *δ* = –4.7 (CH_3_^TBS^), –4.5 (CH_3_^TBS^), 12.6 (CH_3_^T^), 18.1 (qC, *t*-Bu^TBS^), 25.8 (*t*-Bu^TBS^), 40.5 (C-2′), 71.6 (C-3′), 75.8 (C-5′), 85.6 (C-4′), 86.9 (C-1′), 111.6 (C-5), 137.0 (C-6), 150.4 (C-2), 157.5 (CHO), 163.6 (C-4) ppm. HRMS (ESI^+^): *m*/*z* calc. 422.1718 [M + Na]^+^, found: 422.1719.

#### *N*-Formyl-5′-*O*-aminothymidine **6**

Silyl ether **4** (285 mg, 0.71 mmol) was dissolved in THF (7 mL) and the mixture was stirred. TBAF·3H_2_O (400 mg, 1.27 mmol) was added and the resulting opaque mixture was stirred for 18 hours. After this time, TLC analysis (CH_2_Cl_2_/MeOH; 9 : 1) showed the complete consumption of the starting material (*R*_f_ = 0.4) and the formation of the product (*R*_f_ = 0.2). Silica (∼1 g) was added to the flask and solvent was removed *in vacuo* to give the crude product. Purification by column chromatography (CH_2_Cl_2_/MeOH; 85 : 15) gave alcohol **6** as a white hygroscopic foam (189 mg, 93%). IR *ν*_max_ 3389, 3181, 3054, 2922, 2875, 1718, 1655, 1476, 1272, 1093, 1078 cm^–1^. ^1^H NMR (600 MHz, DMSO-*d*_6_): *δ* = 1.79 (d, *J* = 0.9 Hz, 3H, CH_3_^T^), 2.06–2.09 (m, 2H, H-2′a + H-2′b), 3.92–3.96 (m, 2H, H-4′ + H-5′a), 4.00–4.03 (m, 1H, H-5′b), 4.26–4.28 (m, 1H, H-3′), 5.40 (s (br), 1H, OH), 6.19 (app. t, *J* = 7.0 Hz, 1H, H-1′), 7.45 (s (br), 0.15H, H-6), 7.62 (d, *J* = 0.9 Hz, 0.85H, H-6), 7.88 (s, 0.8H, CHO), 8.42 (s, 0.2H, CHO), 11.13–11.39 (m, 2H, NH-3 + O–NH) ppm. Note: compound **6** exhibits rotamers in NMR spectroscopy.

^13^C NMR (150 MHz, DMSO-*d*_6_): *δ* = 12.1 (CH_3_^T^), 39.0 (C-2′), 70.6 (C-3′), 76.1 (C-5′), 83.9 (C-4′), 84.1 (C-1′), 109.9 (C-5), 136.1 (C-6), 150.5 (C-2), 157.4 (CHO), 163.8 (C-4), 165.0 (CHO) ppm. HRMS (ESI^+^): *m*/*z* calc. 308.0853 [M + Na]^+^, found: 308.0856.

#### 5′-Deoxy-3′-*O*-(*tert*-butyldimethylsilyl)-5′-benzyloxyamino-5′oxo-thymidine **9**

Under argon, carboxylic acid **8** (292 mg, 0.79 mmol) was dissolved in anhydrous DMF (4 mL), cooled to 0 °C and the reaction mixture was stirred. EDCI·HCl (203 mg, 1.06 mmol), followed by HOAt (151 mg, 1.11 mmol), benzyloxyamine hydrochloride (177 mg, 1.11 mmol) and DIPEA (0.18 mL, 1.03 mmol) were added and the reaction mixture was stirred for 24 h warming gradually to rt. After this time, TLC analysis (EtOAc/MeOH; 4 : 1) showed the complete consumption of starting material (*R*_f_ = 0.2) and the formation of product (*R*_f_ = 0.7). The reaction mixture was diluted with EtOAc (20 mL) and washed with H_2_O (20 mL). The aqueous layer was extracted with EtOAc (2 × 20 mL) and the combined organic layers were washed with brine (20 mL), dried over MgSO_4_, filtered and solvent was removed *in vacuo* to give the crude product. Purification by column chromatography (PE/EtOAc; 1 : 2) gave benzyloxyamide **9** as a white foam (286 mg, 76%). IR *ν*_max_ 3187, 2929, 2856, 1664, 1470, 1363, 1277, 1252, 1228, 1134, 1066, 993, 920, 832, 778, 742 cm^–1^. ^1^H NMR (400 MHz, CDCl_3_): *δ* = 0.11 (s, 3H, CH_3_^TBS^), 0.12 (s, 3H, CH_3_^TBS^), 0.89 (s, 9H, *t*-Bu^TBS^), 1.92 (s, 3H, CH_3_^T^), 2.01 (dd, *J* = 12.9, 4.8 Hz, 1H, H-2′a), 2.54 (ddd, *J* = 12.5, 9.9, 5.2 Hz, 1H, H-2′b), 4.23–4.28 (m, 1H, H-4′), 4.53 (app. d, *J* = 4.9 Hz, 1H, H-3′), 4.90 (d, *J* = 11.1 Hz, 1H, Ha^benzyl^), 4.99 (d, *J* = 11.1 Hz, 1H, Hb^benzyl^), 5.94 (dd, *J* = 9.5, 5.1 Hz, 1H, H-1′), 7.24 (s, 1H, H-6), 7.31–7.39 (m, 3H, H^Ar^), 7.40–7.45 (m, 2H, H^Ar^), 8.50 (s, 1H, NH-3), 9.76 (s, 1H, O–NH) ppm. ^13^C NMR (100 MHz, CDCl_3_): *δ* = –4.8 (CH_3_^TBS^), –4.7 (CH_3_^TBS^), 12.5 (CH_3_^T^), 18.1 (qC, *t*-Bu^TBS^), 25.8 (*t*-Bu^TBS^), 37.7 (C-2′), 75.6 (C-3′), 78.4 (CH_2_^benzyl^), 86.4 (C-4′), 90.9 (C-1′), 111.8 (C-5), 128.7 (CH^Ar^), 128.9 (CH^Ar^), 129.4 (CH^Ar^), 135.2 (C^Ar^), 138.5 (C-6), 150.5 (C-2), 163.4 (C-4), 167.3 (C-5′) ppm. HRMS (APCI^–^): *m*/*z* calc. 474.2066 [M – H]^–^, found: 474.2073.

#### 5′-Deoxy-5′-benzyloxyamino-5′-oxo-thymidine **10**

Silyl ether **9** (37 mg, 0.08 mmol) was dissolved in THF (1 mL). TBAF·3H_2_O (45 mg, 0.14 mmol) was added and the reaction mixture was stirred at rt overnight. TLC analysis (CH_2_Cl_2_/MeOH; 9 : 1) indicated complete consumption of the starting material (*R*_f_ = 0.7) and the formation of product (*R*_f_ = 0.4). Solvent was removed *in vacuo* and the residue was directly purified by column chromatography (CH_2_Cl_2_/MeOH; 9 : 1) to give free alcohol **10** as a white solid (21 mg, 75%). M.p. 215–222 °C (decomp.). IR *ν*_max_ 3431, 3227, 3058, 2923, 1709, 1655, 1639, 1513, 1474, 1407, 1385, 1293, 1270, 1218, 1132, 1052, 1015 cm^–1^. ^1^H NMR (400 MHz, CD_3_OD): *δ* = 1.91 (d, *J* = 0.9 Hz, 3H, CH_3_^T^), 2.23 (ddd, *J* = 13.7, 5.8, 2.2 Hz, 1H, H-2′a), 2.33 (ddd, *J* = 13.7, 8.6, 5.4 Hz, 1H, H-2′b), 4.17 (d, *J* = 1.5 Hz, 1H, H-4′), 4.40–4.45 (m, 1H, H-3′), 4.90 (s, 2H, H^benzyl^), 6.37 (dd, *J* = 8.6, 5.8 Hz, 1H, H-1′), 7.32–7.41 (m, 3H, H^Ar^), 7.42–7.48 (H^Ar^), 8.00 (d, *J* = 0.9 Hz, 1H, H-6) ppm. ^13^C NMR (100 MHz, CD_3_OD): *δ* = 12.6 (CH_3_^T^), 39.8 (C-2′), 75.4 (C-3′), 79.1 (CH_2_^benzyl^), 85.4 (C-4′), 88.1 (C-1′), 111.8 (C-5), 129.5 (CH^Ar^), 129.8 (CH^Ar^), 130.5 (CH^Ar^), 136.7 (C^Ar^), 138.9 (C-6), 152.5 (C-2), 166.3 (C-4), 169.5 (C-5′) ppm. HRMS (ESI^–^): *m*/*z* calc. 360.1201 [M – H]^–^, found: 360.1199.

#### 5′-Deoxy-5′-hydroxyamino-5′-oxo-thymidine **11**

Benzyloxyamide **10** (18 mg, 0.05 mmol) was dissolved in MeOH (5 mL) and the mixture was purged with argon. 10% Pd/C (8 mg, ∼40% w/w) was added and the reaction mixture was stirred vigorously. The reaction mixture was placed under an atmosphere of hydrogen for 2 hours at rt. TLC analysis (CH_2_Cl_2_/MeOH; 9 : 1) indicated complete consumption of the starting material (*R*_f_ = 0.4) and the formation of product (*R*_f_ = 0.1). The reaction mixture was filtered through Celite® and the filter cake was washed with MeOH (2 × 5 mL). The filtrate was concentrated *in vacuo* to give hydroxamic acid **11** as a white solid (13 mg, 96%). M.p. 168–172 °C (decomp.). IR *ν*_max_ 3490, 3279, 3203, 3061, 2930, 1678, 1652, 1637, 1568, 1478, 1413, 1277, 1238, 1086, 1046, 961, 796 cm^–1^. ^1^H NMR (600 MHz, DMSO-*d*_6_): *δ* = 1.77 (s, 3H, CH_3_^T^), 2.13 (ddd, *J* = 13.3, 5.6, 1.8 Hz, 1H, H-2′a), 2.22 (ddd, *J* = 13.3, 8.6, 5.1 Hz, 1H, H-2′b), 4.07–4.11 (m, 1H, H-4′), 4.27–4.31 (m, 1H, H-3′), 5.68 (s, 1H, OH-3′), 6.32 (dd, *J* = 8.6, 5.6 Hz, 1H, H-1′), 8.16 (s, 1H, H-6), 9.15 (s (br), 1H, O–NH), 11.08 (s (br), 1H, N–OH), 11.29 (s, 1H, NH-3) ppm. ^13^C NMR (150 MHz, DMSO-*d*_6_): *δ* = 12.4 (CH_3_^T^), 39.1 (C-2′), 73.4 (C-3′), 83.5 (C-4′), 84.9 (C-1′), 109.5 (C-5), 136.6 (C-6), 150.6 (C-2), 163.7 (C-4), 166.8 (C-5′) ppm. HRMS (APCI^–^): *m*/*z* calc. 270.0732 [M – H]^–^, found: 270.0723.

#### 3′-*O*-(*tert*-Butyldimethylsilyl)-5′-(trichloromethyl)thymidine **13**

Under argon, aldehyde **12** (346 mg, 0.98 mmol) was dissolved in anhydrous DMF (1.4 mL) and stirred. Trichloroacetic acid (249 mg, 1.52 mmol) followed by sodium trichloroacetate (268 mg, 1.45 mmol) were added and the reaction mixture was stirred at rt for 6 hours. After this time, TLC analysis (PE/EtOAc; 1 : 1) indicated the formation of two diastereomeric products (*R*_f_ = 0.4, 0.3). The reaction mixture was diluted with Et_2_O (10 mL) and washed with sat. aq. NaHCO_3_ solution (3 × 10 mL), brine (10 mL), dried over Na_2_SO_4_, filtered and solvent was removed *in vacuo* to give the crude product. Purification by column chromatography (PE/EtOAc; 1 : 1) gave trichloromethyl carbinol **13** (1 : 1.7 mixture of diastereomers) as a white foam (157 mg, 34%). A small amount of the less polar diastereoisomer [PE/EtOAc; 1 : 1 (*R*_f_ = 0.4)] was isolated by column chromatography which was used for characterisation. IR *ν*_max_ 3352, 2955, 2930, 2886, 2858, 1655, 1472, 1278, 1254, 1201, 1125, 1054, 1004, 834, 816, 777 cm^–1^. ^1^H NMR (400 MHz, CDCl_3_): *δ* = 0.11 (s, 3H, CH_3_^TBS^), 0.12 (s, 3H, CH_3_^TBS^), 0.90 (s, 9H, *t*-Bu^TBS^), 1.92 (s, 3H, CH_3_^T^), 2.11–2.18 (m, 1H, H-2′a), 2.59–2.68 (m, 1H, H-2′b), 4.16–4.23 (m, 1H, H-5′), 4.49–4.56 (m, 2H, H-4′ + H-3′), 4.87 (d, *J* = 7.4 Hz, 1H, OH-5′), 5.93 (app. t, *J* = 7.1 Hz, 1H, H-1′), 7.24 (s, 1H, H-6), 8.86 (s, 1H, NH-3) ppm. ^13^C NMR (100 MHz, CDCl_3_): *δ* = –4.6 (CH_3_^TBS^), –4.5 (CH_3_^TBS^), 12.5 (5-CH_3_), 18.1 (qC, *t*-Bu^TBS^), 25.8 (*t*-Bu^TBS^), 38.5 (C-2′), 75.3 (C-3′), 82.0 (C-5′), 84.8 (C-4′), 91.1 (C-1′), 101.5 (Cl_3_C), 111.5 (C-5), 138.6 (C-6), 150.5 (C-2), 163.7 (C-4) ppm. HRMS (ESI^+^): *m*/*z* calc. 495.0647 [M + Na]^+^, found: 495.0657.

#### 3′-*O*-(*tert*-Butyldimethylsilyl)-5′-(1,3-dithian-2-ylidene)-5′-deoxythymidine **16**

Under argon, 2-(trimethylsilyl)-1,3-dithiane **15** (0.78 mL, 4.11 mmol) was dissolved in anhydrous THF (8 mL) and the reaction mixture was stirred. The reaction mixture was cooled to –78 °C and 1.6 M *n*-BuLi in hexanes (2.4 mL, 3.84 mmol) was added in a dropwise fashion over 5 minutes. The reaction mixture was warmed to 0 °C and stirred for 30 minutes. The reaction mixture was then re-cooled to –78 °C and aldehyde **6** (630 mg, 1.78 mmol) was added as a solution in anhydrous THF (10 mL) in a dropwise fashion after which the reaction turned red. The reaction mixture was warmed to 0 °C and stirred overnight warming gradually to rt. After this time, TLC analysis (PE/EtOAc; 1 : 1) indicated the formation of product (*R*_f_ = 0.3). The reaction mixture was quenched with half-saturated NH_4_Cl solution (10 mL) and extracted with Et_2_O (2 × 10 mL). The combined ethereal layers were washed with brine (10 mL), dried over MgSO_4_, filtered and solvent was removed *in vacuo* to give the crude product as an orange oil. Purification by column chromatography (PE/EtOAc; 1 : 1) gave ketene dithioacetal **16** (mixture of 4′-epimers; dr 2 : 1) as an unstable yellow foam (135 mg, 17%). This material was used immediately for the next step. IR *ν*_max_ 3178, 2955, 2928, 2855, 1685, 1583, 1470, 1421, 1362, 1271, 1251, 1190, 1108, 1046, 915, 833, 776 cm^–1^. ^1^H NMR (400 MHz, CDCl_3_): *δ* = 0.06 (s, 2H, CH_3_^TBS^), 0.07 (s, 2H, CH_3_^TBS^), 0.09 (s, 2H, CH_3_^TBS^), 0.89 (s, 6H, *t*-Bu^TBS^), 0.89 (s, 3H, *t*-Bu^TBS^), 1.94 (d, *J* = 1.2 Hz, 3H, CH_3_^T^), 2.05 (dd, *J* = 13.5, 6.6 Hz, 0.67H, H-2′a), 2.09–2.23 (m, 2.33H, H-2′a + CH_2_^dithiane^), 2.37 (ddd, *J* = 13.5, 6.3, 3.9 Hz, 0.67H, H-2′b), 2.51 (ddd, *J* = 13.7, 6.0, 1.1 Hz, 0.33H, H-2′b), 2.81–3.06 (m, 4H, CH_2_S^dithiane^), 4.15 (app. dt, *J* = 6.3, 3.9 Hz, 0.67H, H-3′), 4.36–4.40 (m, 0.33H, H-3′), 4.83 (dd, *J* = 8.9, 3.6 Hz, 0.67H, H-4′), 5.14 (dd, *J* = 8.0, 3.1 Hz, 0.33H, H-4′), 5.77 (d, *J* = 8.9 Hz, 0.67H, H-5′), 5.97 (d, *J* = 8.0 Hz, 0.33H, H-5′), 6.17 (app. t, *J* = 6.6 Hz, 0.67H, H-1′), 6.19–6.23 (m, 0.33H, H-1′), 7.18 (d, *J* = 1.2 Hz, 0.67H, H-6), 7.23 (d, *J* = 1.2 Hz, 0.33H, H-6), 8.56 (s, 1H, NH-3) ppm. Note: ^1^H NMR spectrum complicated due to mixture of 4′-epimers. HRMS (ESI^+^): *m*/*z* calc. 479.1465 [M + Na]^+^, found: 479.1457.

#### 5′-Deoxy-3′-*O*-(*tert*-butyldimethylsilyl)-5′-methoxycarbonyl-thymidine **17**

Ketene-dithioacetal **16** (91 mg, 0.20 mmol) was dissolved in MeOH (1 mL). AgNO_3_ (117 mg, 0.69 mmol) was added and the reaction mixture was stirred at rt overnight in the dark. After this time, TLC analysis (PE/EtOAc; 1 : 1) indicated complete consumption of starting material (*R*_f_ = 0.35) and the formation of product (*R*_f_ = 0.3). The reaction mixture was filtered through Celite® and the cake was washed with EtOAc (5 mL). The filtrate was azeotropically evaporated with toluene (10 mL). The residue was re-dissolved in EtOAc (10 mL) and the organic layer was washed with brine (5 mL), dried over MgSO_4_, filtered and solvent was removed *in vacuo* to give the crude product as a complex mixture. Purification by column chromatography (PE/EtOAc; 1 : 1) gave methyl ester **17** as a colourless wax (11 mg, 14%). IR *ν*_max_ 2954, 2929, 2856, 1686, 1464, 1438, 1362, 1306, 1271, 1192, 1174, 1049, 984, 834, 777 cm^–1^. ^1^H NMR (400 MHz, CDCl_3_): *δ* = 0.05 (s, 3H, CH_3_^TBS^), 0.10 (s, 3H, CH_3_^TBS^), 0.90 (s, 9H, *t*-Bu^TBS^), 1.94 (d, *J* = 1.0 Hz, 3H, CH_3_^T^), 2.15 (ddd, *J* = 14.0, 7.4, 4.7 Hz, 1H, H-2′a), 2.49 (ddd, *J* = 14.0, 6.1, 1.3 Hz, 1H, H-2′b), 2.70 (dd, *J* = 16.0, 5.9 Hz, 1H, H-5′a), 2.76 (dd, *J* = 16.0, 6.4 Hz, 1H, H-5′b), 3.71 (s, 3H, OCH_3_), 4.48–4.52 (m, 1H, H-3′), 4.60 (app. td, *J* = 6.4, 3.2 Hz, 1H, H-4′), 6.15 (dd, *J* = 7.4, 6.1 Hz, 1H, H-1′), 7.18 (d, *J* = 1.0 Hz, 1H, H-6), 8.33 (s, 1H, NH-3) ppm. ^13^C NMR (100 MHz, CDCl_3_): *δ* = –5.1 (CH_3_^TBS^), –4.5 (CH_3_^TBS^), 12.8 (CH_3_^T^), 18.1 (qC, *t*-Bu^TBS^), 25.8 (*t*-Bu^TBS^), 34.2 (C-5′), 42.7 (C-2′), 51.9 (OCH_3_), 72.5 (C-3′), 81.0 (C-4′), 86.5 (C-1′), 111.0 (C-5), 135.3 (C-6), 150.1 (C-2), 163.7 (C-4), 171.6 (CO^ester^) ppm. HRMS (ESI^+^): *m*/*z* calc. 421.1765 [M + Na]^+^, found: 421.1770.

#### 3′-*O*-(*tert*-Butyldimethylsilyl)-5′-benzyloxycarbonylmethylene-5′-deoxythymidine **19**

Under argon, aldehyde **12** (683 mg, 1.93 mmol) was dissolved in anhydrous CH_2_Cl_2_ (20 mL) and the mixture was stirred. Ylide **18** (1.02 g, 2.49 mmol) was added and the reaction mixture was stirred at rt overnight. After this time, TLC analysis (PE/EtOAc; 1 : 1) showed the formation of product (*R*_f_ = 0.4). Solvent was removed *in vacuo* to give the crude product. Purification by column chromatography (PE/EtOAc; 1 : 1) gave the α,β-unsaturated ester **19** as a white foam (761 mg, 81%). IR *ν*_max_ 3348, 3204, 2954, 2929, 2887, 2857, 1719, 1688, 1652, 1473, 1361, 1267, 1241, 1176, 1028, 833 cm^–1^. ^1^H NMR (400 MHz, CDCl_3_) *δ* = 0.08 (s, 6H, 2 × CH_3_^TBS^), 0.89 (s, 9H, *t*-Bu^TBS^), 1.92 (d, *J* = 1.2 Hz, 3H, CH_3_^T^), 2.12 (app. dt, *J* = 13.6, 6.7 Hz, 1H, H-2′a), 2.31 (ddd, *J* = 13.6, 6.5, 4.3 Hz, 1H, H-2′b), 4.24 (app. dt, *J* = 6.7, 4.3 Hz, 1H, H-3′), 4.39 (app. td, *J* = 5.2, 1.7 Hz, 1H, H-4′), 5.22 (s, 2H, H^benzyl^), 6.16 (dd, *J* = 15.7, 1.7 Hz, 1H, H-α), 6.29 (t, *J* = 6.7 Hz, 1H, H-1′), 7.00 (dd, *J* = 15.7, 5.2 Hz, 1H, H-β), 7.09 (d, *J* = 1.2 Hz, 1H, H-6), 7.31–7.40 (m, 5H, H^Ar^), 8.74 (s, 1H, NH-3) ppm. ^13^C NMR (100 MHz, CDCl_3_) *δ* = –4.7 (CH_3_^TBS^), –4.5 (CH_3_^TBS^), 12.8 (CH_3_^T^), 18.1 (qC, *t*-Bu^TBS^), 25.8 (*t*-Bu^TBS^), 40.3 (C-2′), 66.7 (CH_2_^benzyl^), 75.1 (C-3′), 85.27 (C-1′), 85.33 (C-4′), 111.6 (C-5), 122.4 (CH-α), 128.4 (CH^Ar^), 128.5 (CH^Ar^), 128.7 (CH^Ar^), 135.2 (C-6), 135.8 (C^Ar^), 144.1 (CH-β), 150.2 (C-2), 163.6 (C-4), 165.7 (CO^ester^). HRMS (ESI^+^): *m*/*z* calc. 509.2078 [M + Na]^+^, found: 509.2074.

#### 5′-Deoxy-3′-*O*-(*tert*-butyldimethylsilyl)-thymidin-yl-acetic acid **20**

Under argon, α,β-unsaturated ester **19** (378 mg, 0.78 mmol) was dissolved in MeOH (8 mL) and the mixture was stirred. 10% Pd/C (52 mg, ∼15% w/w) was added and the reaction mixture was placed under an atmosphere of hydrogen at rt for 4 hours. After this time, TLC analysis (CH_2_Cl_2_/MeOH; 19 : 1) showed complete consumption of the starting material (*R*_f_ = 0.8) and the formation of product (*R*_f_ = 0.2). The reaction mixture was filtered through Celite® and the filter cake was washed with methanol (3 × 10 mL). The filtrate was concentrated *in vacuo* to give carboxylic acid **20** as a white foam (300 mg, 97%). IR *ν*_max_ 3179, 2954, 2930, 1686, 1472, 1273, 1259, 1095, 1062, 834, 777 cm^–1^. ^1^H NMR (400 MHz, DMSO-*d*_6_): *δ* = 0.08 (s, 3H, CH_3_^TBS^), 0.08 (s, 3H, CH_3_^TBS^), 0.87 (s, 9H, *t*-Bu^TBS^), 1.71–1.94 (m, 5H, H-5′ + CH_3_^T^), 2.02 (ddd, *J* = 13.0, 6.5, 4.1 Hz, 1H, H-2′a), 2.21–2.36 (m, 3H, H-2′b + CH_2_-α), 3.61–3.69 (m, 1H, H-4′), 4.20–4.27 (m, 1H, H-3′), 6.09 (app. t, *J* = 6.5 Hz, 1H, H-1′), 7.42 (s, 1H, H-6), 11.29 (s, 1H, NH-3) ppm. ^13^C NMR (100 MHz, DMSO-*d*_6_): *δ* = –4.9 (CH_3_^TBS^), –4.7 (CH_3_^TBS^), 12.1 (CH_3_^T^), 17.6 (qC, *t*-Bu^TBS^), 25.7 (*t*-Bu^TBS^), 28.1 (C-5′), 30.2 (CH_2_-α), 38.7 (C-2′), 74.4 (C-3′), 83.4 (C-1′), 84.9 (C-4′), 109.8 (C-5), 136.2 (C-6), 150.4 (C-2), 163.7 (C-4), 174.1 (CO_2_H) ppm. HRMS (ESI^+^): *m*/*z* calc. 421.1765 [M + Na]^+^, found: 421.1767.

#### *N*-(Benzyloxy)-5′-deoxy-3′-*O*-(*tert*-butyldimethylsilyl)-thymidin-yl-acetamide **21**

Under argon, carboxylic acid **20** (257 mg, 0.64 mmol) was dissolved in anhydrous DMF (3 mL) and the mixture was stirred at rt. *O*-Benzylhydroxylamine hydrochloride (157 mg, 0.98 mmol), EDCI·HCl (186 mg, 0.97 mmol), HOAt (132 mg, 0.97 mmol) and DIPEA (0.34 mL, 1.95 mmol) were added and the reaction mixture was stirred overnight at rt. TLC analysis (CH_2_Cl_2_/MeOH; 19 : 1) indicated consumption of starting material (*R*_f_ = 0.2) and the formation of product (*R*_f_ = 0.7). The reaction mixture was diluted with EtOAc (20 mL) and washed successively with NaHCO_3_ sat. aq. solution (10 mL) and brine (10 mL). The layers were separated and the organic layer was dried over MgSO_4_, filtered and solvent was removed *in vacuo* to give the crude product. Purification by column chromatography (PE/EtOAc; 1 : 2 → EtOAc) gave hydroxamate **21** as a white foam (175 mg, 54%). IR *ν*_max_ 3188, 2953, 2929, 2856, 1655, 1471, 1362, 1273, 1252, 1195, 833, 777 cm^–1^. ^1^H NMR (600 MHz, DMSO-*d*_6_): *δ* = 0.08 (s, 3H, CH_3_^TBS^), 0.09 (s, 3H, CH_3_^TBS^), 0.87 (s, 9H, *t*-Bu^TBS^), 1.71–1.78 (m, 1H, H-5′a), 1.80 (s, 3H, CH_3_^T^), 1.86–1.93 (m, 1H, H-5′b), 1.99–2.14 (m, 3H, H-2′a + CH_2_-α), 2.25 (app. dt, *J* = 13.6, 6.9 Hz, 1H, H-2′b), 3.62 (app. dt, *J* = 8.4, 3.8 Hz, 1H, H-4′), 4.21 (app. dt, *J* = 6.9, 3.8 Hz, 1H, H-3′), 4.75 (d, *J* = 11.1 Hz, 1H, Ha^benzyl^), 4.77 (d, *J* = 11.1 Hz, 1H, Hb^benzyl^), 6.10 (app. t, *J* = 6.9 Hz, 1H, H-1′), 7.31–7.39 (m, 5H, H^Ar^), 7.42 (s, 1H, H-6), 10.99 (s, 1H, NH-3), 11.28 (s, 1H, O–NH) ppm. ^13^C NMR (150 MHz, DMSO-*d*_6_): *δ* = –4.8 (Si–CH_3_), –4.7 (Si–CH_3_), 12.1 (CH_3_^T^), 17.6 (qC, *t*-Bu^TBS^), 25.7 (*t*-Bu^TBS^), 28.4 (C-5′), 28.9 (CH_2_-α), 38.7 (C-2′), 74.5 (C-3′), 76.7 (CH_2_^benzyl^), 83.4 (C-1′), 85.1 (C-4′), 109.8 (C-5), 128.2 (CH^Ar^), 128.3 (CH^Ar^), 128.7 (CH^Ar^), 136.0 (C^Ar^), 136.2 (C-6), 150.4 (C-2), 163.7 (C-4), 168.9 (CO–NH–O) ppm. HRMS (ESI^–^): *m*/*z* calc. 502.2379 [M – H]^–^, found: 502.2379.

#### *N*-(Benzyloxy)-5′-deoxythymidin-yl-acetamide **22**

Silyl ether **21** (170 mg, 0.34 mmol) was dissolved in THF (3.5 mL). TBAF·3H_2_O (192 mg, 0.61 mmol) was added and the reaction mixture was stirred overnight at rt. TLC analysis (CH_2_Cl_2_/MeOH; 9 : 1) indicated consumption of starting material (*R*_f_ = 0.6) and the formation of product (*R*_f_ = 0.2). Solvent was removed *in vacuo* to give the crude product. Purification by column chromatography (EtOAc/MeOH; 9 : 1) gave alcohol **22** as a white solid (105 mg, 80%). M.p. 176–180 °C (decomp.). IR *ν*_max_ 3443, 3212, 2932, 1711, 1647, 1473, 1262, 1048 cm^–1^. ^1^H NMR (600 MHz, DMSO-*d*_6_): *δ* = 1.71–1.78 (m, 1H, H-5′a), 1.80 (s, 3H, CH_3_^T^), 1.86–1.94 (m, 1H, H-5′b), 2.00–2.12 (m, 3H, H-2′a + CH_2_-α), 2.16 (app. dt, *J* = 13.7, 6.9 Hz, 1H, H-2′b), 3.61 (app. dt, *J* = 8.4, 3.8 Hz, 1H, H-4′), 4.04 (app. dt, *J* = 7.0, 3.8 Hz, 1H, H-3′), 4.76 (s, 2H, CH_2_^benzyl^), 5.27 (s (br), 1H, OH-3′), 6.12 (app. t, *J* = 6.9 Hz, 1H, H-1′), 7.32–7.39 (m, 5H, H^Ar^), 7.41 (s, 1H, H-6), 11.00 (s (br), 1H, NH-3), 11.26 (s (br), 1H, O–NH) ppm. ^13^C NMR (150 MHz, DMSO-*d*_6_): *δ* = 12.1 (CH_3_^T^), 28.7 (C-5′), 28.9 (CH_2_-α), 38.4 (C-2′), 73.0 (C-3′), 76.8 (CH_2_^benzyl^), 83.3 (C-1′), 85.2 (C-4′), 109.8 (C-5), 128.2 (CH^Ar^), 128.3 (CH^Ar^), 128.8 (CH^Ar^), 136.08 (C^Ar^), 136.1 (C-6), 150.5 (C-2), 163.7 (C-4), 169.0 (CO–NH–O) ppm. HRMS (APCI^–^): *m*/*z* calc. 388.1514 [M – H]^–^, found: 388.1502.

#### *N*-(Hydroxy)-5′-deoxythymidin-yl-acetamide **23**

Under argon, hydroxamate **22** (28 mg, 0.07 mmol) was dissolved in MeOH (12 mL) and the solution was stirred. Pd/C (4 mg, ∼15% w/w) was added and the reaction mixture was placed under an atmosphere of hydrogen for 1 hour. After this time, TLC analysis (CH_2_Cl_2_/MeOH; 4 : 1) indicated complete consumption of starting material (*R*_f_ = 0.4) and the formation of product (*R*_f_ = 0.2). The reaction mixture was filtered and the filtrate was concentrated *in vacuo* to give hydroxamic acid **23** as white solid (19 mg, 88%). M.p. 169–174 °C (decomp.). IR *ν*_max_ 3394, 3210, 3049, 1654, 1476, 1420, 1262, 1078 cm^–1^. ^1^H NMR (600 MHz, DMSO-*d*_6_): *δ* = 1.68–1.77 (m, 1H, H-5′a), 1.80 (s, 3H, CH_3_^T^), 1.85–1.93 (m, 1H, H-5′b), 1.98–2.12 (m, 3H, CH_2_-α + H-2′a), 2.17 (app. dt, *J* = 13.7, 7.0 Hz, 1H, H-2′b), 3.60 (app. dt, *J* = 8.8, 4.4 Hz, 1H, H-4′), 4.01–4.07 (m, 1H, H-3′), 5.27 (d, *J* = 4.4 Hz, 1H, OH-3′), 6.12 (app. t, *J* = 7.0 Hz, 1H, H-1′), 7.40 (s, 1H, H-6), 8.69 (s, 0.9H, N–OH), 9.04 (s, 0.1H, N–OH), 9.81 (s, 0.1H, O–NH), 10.38 (s, 0.9H, O–NH), 11.28 (s, 1H, NH-3) ppm. Note: compound **3** exhibits rotamers in NMR spectroscopy. ^13^C NMR (150 MHz, DMSO-*d*_6_): *δ* = 12.1 (CH_3_^T^), 28.9 (CH_2_-α), 29.0 (C-5′), 38.4 (C-2′), 73.0 (C-3′), 83.3 (C-1′), 85.1 (C-4′), 109.9 (C-5), 136.1 (C-6), 150.5 (C-2), 163.7 (C-4), 168.6 (CO–NH–O) ppm. HRMS (ESI^+^): *m*/*z* calc. 322.1010 [M + Na]^+^, found: 322.0996.

#### 5′-Deoxy-3′-*O*-(*tert*-butyldimethylsilyl)-5′-hydroxyamino-5′-oxo-thymidine **24**

Benzyloxyamide **9** (733 mg, 1.54 mmol) was dissolved in MeOH (116 mL) and the mixture was purged with argon. 10% Pd/C (118 mg, ∼15% w/w) was added and the reaction mixture was stirred vigorously. The reaction mixture was placed under an atmosphere of hydrogen for 3 hours at rt. TLC analysis (EtOAc) indicated complete consumption of the starting material (*R*_f_ = 0.6) and the formation of product (*R*_f_ = 0.4). The reaction mixture was filtered through Celite® and the filter cake was washed with methanol (2 × 30 mL). The filtrate was concentrated *in vacuo* and the residue was purified by column chromatography (EtOAc) to give hydroxamic acid **24** as a white foam (522 mg, 88%). IR *ν*_max_ 3188, 3056, 2927, 2855, 1690, 1648, 1473, 1427, 1382, 1275, 1255, 1167, 1093, 1045, 1014, 987, 900, 866, 835, 776, 670 cm^–1^. ^1^H NMR (600 MHz, DMSO-*d*_6_): *δ* = 0.09 (s, 3H, CH_3_^TBS^), 0.10 (s, 3H, CH_3_^TBS^), 0.87 (s, 9H, *t*-Bu^TBS^), 1.77 (d, *J* = 1.0 Hz, 3H, CH_3_^T^), 2.11 (ddd, *J* = 13.1, 5.6, 2.0 Hz, 1H, H-2′a), 2.33 (ddd, *J* = 13.1, 8.6, 5.2 Hz, 1H, H-2′b), 4.06 (d, *J* = 2.0 Hz, 1H, H-4′), 4.44–4.47 (m, 1H, H-3′), 6.29 (dd, *J* = 8.6, 5.6 Hz, 1H, H-1′), 8.07 (d, *J* = 1.0 Hz, 1H, H-6), 9.19 (s, 1H, N–OH), 11.08 (s, 1H, O–NH), 11.33 (s, 1H, NH-3) ppm. ^13^C NMR (150 MHz, DMSO-*d*_6_): *δ* = –5.0 (2 × CH_3_^TBS^), 12.4 (CH_3_^T^), 17.7 (qC, *t*-Bu^TBS^), 25.6 (*t*-Bu^TBS^), 39.2 (C-2′), 74.9 (C-3′), 83.6 (C-4′), 84.9 (C-1′), 109.6 (C-5), 136.5 (C-6), 150.6 (C-2), 163.7 (C-4), 166.1 (C-5′) ppm. HRMS (APCI^–^): *m*/*z* calc. 384.1596 [M – H]^–^, found: 384.1603.

#### 5′-Deoxy-3′-*O*-(*tert*-butyldimethylsilyl)-5′-acetoxyamino-5′-oxo-thymidine **25**

Hydroxamic acid **24** (391 mg, 1.01 mmol) was dissolved in anhydrous THF (2.5 mL) and the reaction mixture was stirred. Pyridine (114 μL, 1.42 mmol) was added, followed by acetic anhydride (105 μL, 1.12 mmol) and the reaction mixture was stirred for 20 minutes at rt. TLC analysis (EtOAc) indicated complete consumption of the starting material (*R*_f_ = 0.4) and the formation of product (*R*_f_ = 0.5). The reaction mixture was diluted with EtOAc (10 mL) and the mixture was washed with brine (10 mL). The layers were separated and the aqueous layer was extracted with EtOAc (10 mL). The organic layers were combined and dried over MgSO_4_, filtered and solvent was removed *in vacuo* to give crude product. Purification by column chromatography gave acetate **25** as a white foam (388 mg, 89%). IR *ν*_max_ 3184, 2956, 2931, 2857, 1793, 1676, 1466, 1367, 1277, 1177, 1067, 993, 921, 833, 777 cm^–1^. ^1^H NMR (600 MHz, CDCl_3_): *δ* = 0.13 (s, 3H, CH_3_^TBS^), 0.15 (s, 3H, CH_3_^TBS^), 0.91 (s, 9H, *t*-Bu^TBS^), 1.92 (d, *J* = 1.0 Hz, 3H, CH_3_^T^), 2.08 (dd, *J* = 13.1, 5.1 Hz, 1H, H-2′a), 2.24 (s, 3H, CH_3_^acetate^), 2.63 (ddd, *J* = 13.1, 10.1, 5.1 Hz, 1H, H-2′b), 4.45–4.47 (m, 1H, H-4′), 4.74 (d, *J* = 5.1 Hz, 1H, H-3′), 6.12 (dd, *J* = 10.1, 5.1 Hz, 1H, H-1′), 7.29 (app. s, 1H, H-6), 8.66 (s, 1H, NH-3), 10.56 (s, 1H, O–NH) ppm. ^13^C NMR (150 MHz, CDCl_3_): *δ* = –4.8 (CH_3_^TBS^), –4.7 (CH_3_^TBS^), 12.4 (CH_3_^T^), 18.1 (qC, *t*-Bu^TBS^), 18.4 (CH_3_^acetate^), 25.9 (*t*-Bu^TBS^), 37.4 (C-2′), 75.8 (C-3′), 86.7 (C-4′), 90.7 (C-1′), 112.2 (C-5), 138.3 (C-6), 150.8 (C-2), 163.5 (C-4), 167.5 (C-5′), 168.8 (CO^acetate^) ppm. HRMS (ESI^+^): *m*/*z* calc. 450.1667 [M + Na]^+^, found: 450.1667.

#### 5′-Deoxy-5′-acetoxyamino-5′-oxo-thymidine **26**

Silyl ether **25** (345 mg, 0.81 mmol) was dissolved in THF (8 mL), cooled to 0 °C and stirred. Acetic acid (166 μL, 2.90 mmol) followed by TBAF·3H_2_O (461 mg, 1.46 mmol) were added and the reaction mixture was stirred at rt overnight. TLC analysis (EtOAc) indicated complete consumption of the starting material (*R*_f_ = 0.5) and the formation of product (*R*_f_ = 0.1). Methanol (15 mL) was added to the flask, followed by silica (∼1 g) and solvent was removed *in vacuo*. Purification by column chromatography (CH_2_Cl_2_/MeOH; 19 : 1 → 4 : 1) gave alcohol **26** as a white powder (94 mg, 37%). M.p. 122–125 °C (decomp.). IR *ν*_max_ 3456, 3230, 3060, 1799, 1706, 1654, 1636, 1476, 1408, 1366, 1334, 1293, 1274, 1216, 1177, 1132, 1110, 955, 778 cm^–1^. ^1^H NMR (400 MHz, DMSO-*d*_6_): *δ* = 1.76 (s, 3H, CH_3_^T^), 2.14 (dd, *J* = 7.2, 3.2 Hz, 2H, H-2′a + H-2′b), 2.19 (s, 3H, CH_3_^acetate^), 4.27–4.30 (m, 1H, H-4′), 4.39–4.44 (m, 1H, H-3′), 5.76 (d, *J* = 4.4 Hz, 1H, OH-3′), 6.37 (t, *J* = 7.3 Hz, 1H, H-1′), 7.91 (s, 1H, H-6), 11.34 (s, 1H, NH-3), 12.21 (s (br), 1H, O–NH) ppm. ^13^C NMR (100 MHz, DMSO-*d*_6_): *δ* = 12.3 (CH_3_^T^), 18.1 (CH_3_^acetate^), 38.3 (C-2′), 73.6 (C-3′), 83.3 (C-4′), 85.2 (C-1′), 109.7 (C-5), 136.4 (C-6), 150.7 (C-2), 163.7 (C-4), 167.4 (C-5′), 168.3 (CO^acetate^) ppm. HRMS (APCI^–^): *m*/*z* calc. 312.0837 [M – H]^–^, found: 312.0829.

#### 5′-Deoxy-3′-*O*-(*tert*-butyldimethylsilyl)-5′-methoxyamino-5′-oxo-thymidine **27**

Under argon, carboxylic acid **8** (402 mg, 1.09 mmol) was dissolved in anhydrous DMF (5 mL), cooled to 0 °C and stirred. EDCI·HCl (267 mg, 1.39 mmol) followed by HOAt (194 mg, 1.43 mmol), methoxyamine hydrochloride (119 mg, 1.42 mmol) and DIPEA (0.25 mL, 1.40 mmol) were added and the reaction mixture was stirred overnight warming gradually to rt. After this time, TLC analysis (EtOAc/MeOH; 4 : 1) indicated complete consumption of the starting material (*R*_f_ = 0.2) and the formation of product (*R*_f_ = 0.6). The reaction mixture was diluted with EtOAc (30 mL) and washed with H_2_O (30 mL). The aqueous layer was extracted with EtOAc (2 × 30 mL) and the combined organic layers were washed with brine (30 mL), dried over MgSO_4_, filtered and solvent was removed *in vacuo* to give the crude product. Purification by column chromatography (CH_2_Cl_2_/MeOH; 199 : 1 → 19 : 1) gave methoxyamide **27** as a white foam (331 mg, 76%). IR *ν*_max_ 3184, 2930, 2857, 1664, 1471, 1277, 1252, 1228, 1191, 1134, 1066, 994, 919, 832, 777 cm^–1^. ^1^H NMR (400 MHz, CDCl_3_): *δ* = 0.13 (s, 3H, CH_3_^TBS^), 0.14 (s, 3H, CH_3_^TBS^), 0.90 (s, 9H, *t*-Bu^TBS^), 1.95 (s, 3H, CH_3_^T^), 2.05 (dd, *J* = 12.9, 4.8 Hz, 1H, H-2′a), 2.67 (ddd, *J* = 12.9, 9.8, 5.1 Hz, 1H, H-2′b), 3.79 (s, 3H, OCH_3_), 4.28–4.33 (m, 1H, H-4′), 4.64 (d, *J* = 4.8 Hz, 1H, H-3′), 5.95 (dd, *J* = 9.8, 5.1 Hz, 1H, H-1′), 7.26 (s, 1H, H-6), 8.69 (s, 1H, O–NH), 10.03 (s, 1H, NH-3) ppm. Note: H-6 coincident with residual CHCl_3_ in the ^1^H NMR spectrum. ^13^C NMR (100 MHz, CDCl_3_): *δ* = –4.8 (CH_3_^TBS^), –4.7 (CH_3_^TBS^), 12.5 (CH_3_^T^), 18.1 (qC, *t*-Bu^TBS^), 25.8 (*t*-Bu^TBS^), 37.6 (C-2′), 64.5 (OCH_3_), 75.6 (C-3′), 86.5 (C-4′), 91.4 (C-1′), 111.9 (C-5), 138.8 (C-6), 150.7 (C-2), 163.5 (C-4), 167.3 (C-5′) ppm. HRMS (APCI^–^): *m*/*z* calc. 398.1753 [M – H]^–^, found: 398.1756.

#### 5′-Deoxy-5′-methoxyamino-5′-oxo-thymidine **28**

Silyl ether **27** (309 mg, 0.77 mmol) was dissolved in THF (8 mL) and the mixture was stirred. TBAF·3H_2_O (448 mg, 1.42 mmol) was added and the reaction mixture was stirred overnight at rt. TLC analysis (CH_2_Cl_2_/MeOH; 9 : 1) indicated complete consumption of the starting material (*R*_f_ = 0.5) and the formation of product (*R*_f_ = 0.1). Methanol (5 mL), followed by silica (∼3 g) were added to the flask. Solvent was removed *in vacuo* to give the crude product which was purified by column chromatography (CH_2_Cl_2_/MeOH; 19 : 1 → 9 : 1) to give alcohol **28** as a white foam (211 mg, 96%). IR *ν*_max_ 3469, 3424, 3234, 3061, 2937, 1655, 1637, 1512, 1475, 1405, 1335, 1293, 1270, 1222, 1134, 1051, 967, 920, 795, 774 cm^–1^. ^1^H NMR (400 MHz, DMSO-*d*_6_): *δ* = 1.78 (s, 3H, CH_3_^T^), 2.12 (ddd, *J* = 13.3, 5.7, 1.7 Hz, 1H, H-2′a), 2.22 (ddd, *J* = 13.3, 8.6, 5.2 Hz, 1H, H-2′b), 3.62 (s, 3H, OCH_3_), 4.05 (d, *J* = 1.1 Hz, 1H, H-4′), 4.33–4.38 (m, 1H, H-3′), 5.67 (d, *J* = 4.1 Hz, 1H, OH-3′), 6.32 (dd, *J* = 8.6, 5.7 Hz, 1H, H-1′), 8.00 (s, 1H, H-6), 11.33 (s (br), 1H, NH-3), 11.54 (s (br), 1H, O–NH) ppm. ^13^C NMR (100 MHz, DMSO-*d*_6_): *δ* = 12.4 (CH_3_^T^), 38.6 (C-2′), 63.3 (O–CH_3_), 73.3 (C-3′), 83.4 (C-4′), 85.1 (C-1′), 109.6 (C-5), 136.6 (C-6), 150.6 (C-2), 163.7 (C-4), 166.8 (C-5′) ppm. HRMS (APCI^–^): *m*/*z* calc. 284.0888 [M – H]^–^, found: 284.0892.

#### 5′-Deoxy-3′-*O*-(*tert*-butyldimethylsilyl)-5′-(Fmoc-hydrazino)-5′-oxo-thymidine **29**

Under argon, carboxylic acid **8** (360 mg, 0.97 mmol) was dissolved in anhydrous DMF (5 mL), cooled to 0 °C and stirred. EDCI·HCl (243 mg, 1.27 mmol) followed by HOAt (176 mg, 1.29 mmol) and Fmoc-hydrazide **S5** (307 mg, 1.21 mmol) were added and the reaction mixture was stirred overnight warming gradually to rt. TLC analysis (EtOAc/MeOH; 4 : 1) indicated complete consumption of the starting material (*R*_f_ = 0.2) and the formation of product (*R*_f_ = 0.7). The reaction mixture was diluted with EtOAc (20 mL) and washed with H_2_O (20 mL). The aqueous layer was extracted with EtOAc (2 × 20 mL) and the combined organic layers were washed with brine (20 mL), dried over MgSO_4_, filtered and solvent was removed *in vacuo* to give the crude product. Purification by column chromatography (PE/EtOAc; 1 : 2) gave hydrazide **29** as a white foam (414 mg, 70%). IR *ν*_max_ 3244, 2952, 2929, 2857, 1677, 1470, 1450, 1247, 1101, 1070, 995, 834, 778, 758, 739 cm^–1^. ^1^H NMR (600 MHz, CDCl_3_): *δ* = 0.13 (s, 3H, CH_3_^TBS^), 0.15 (s, 3H, CH_3_^TBS^), 0.91 (s, 9H, *t*-Bu^TBS^), 1.89 (s, 3H, CH_3_^T^), 2.07 (d, *J* = 9.6 Hz, 1H, H-2′a), 2.43–2.58 (m, 1H, H-2′b), 4.24 (t, *J* = 7.2 Hz, 1H, CH^Fmoc^), 4.40–4.48 (m, 3H, CH_2_^Fmoc^ + H-4′), 4.75–4.86 (m, 1H, H-3′), 6.22–6.39 (m, 1H, H-1′), 7.05 (s, 1H, NH^Fmoc^), 7.29 (app. t, *J* = 7.5 Hz, 2H, H^Ar^), 7.39 (app. t, *J* = 7.5 Hz, 2H, H^Ar^), 7.45 (s, 1H, H-6), 7.57 (d, *J* = 7.5 Hz, 1H, H^Ar^), 7.59 (d, *J* = 7.5 Hz, 1H, H^Ar^), 7.75 (d, *J* = 7.5 Hz, 2H, H^Ar^), 8.82 (s (br), 1H, CO–NH), 8.97 (s (br), 1H, NH-3) ppm. ^13^C NMR (150 MHz, CDCl_3_): *δ* = –4.8 (CH_3_^TBS^), –4.7 (CH_3_^TBS^), 12.3 (CH_3_^T^), 18.1 (qC, *t*-Bu^TBS^), 25.9 (*t*-Bu^TBS^), 37.9 (C-2′), 47.0 (CH^Fmoc^), 68.3 (CH_2_^Fmoc^), 75.6 (C-3′), 86.6 (C-4′), 89.4 (C-1′), 112.4 (C-5), 120.2 (CH^Ar^), 125.2 (CH^Ar^), 125.3 (CH^Ar^), 127.3 (CH^Ar^), 128.0 (CH^Ar^), 137.5 (C-6), 141.39 (C^Ar^), 141.43 (C^Ar^), 143.5 (C^Ar^), 143.6 (C^Ar^), 151.0 (C-2), 156.4 (CO^Fmoc^), 163.6 (C-4), 169.9 (C-5′) ppm. HRMS (ESI^+^): *m*/*z* calc. 629.2402 [M + Na]^+^, found: 629.2410.

#### 5′-Deoxy-5′-(Fmoc-hydrazino)-5′-oxo-thymidine **30**

Silyl ether **29** (325 mg, 0.54 mmol) was dissolved in THF (4.5 mL), cooled to 0 °C and stirred. Acetic acid (74 μL, 1.29 mmol) followed by TBAF·3H_2_O (202 mg, 0.64 mmol) were added and the reaction mixture was stirred for 4 hours at rt with monitoring by TLC. TLC analysis (EtOAc) indicated partial consumption of the starting material (*R*_f_ = 0.6) and the formation of product (*R*_f_ = 0.2). A blue fluorescent spot at the top of the TLC plate indicated the beginning of Fmoc-deprotection and the reaction was halted. Methanol (10 mL) followed by silica (∼1 g) were added to the flask. Solvent was removed *in vacuo* and the residue was purified by column chromatography (EtOAc) to give alcohol **30** as a white powder (69 mg, 26%). M.p. 215–218 °C (decomp.). IR *ν*_max_ 3569, 3258, 3036, 2647, 1750, 1696, 1656, 1478, 1436, 1281, 1234, 1101, 1076, 739 cm^–1^. ^1^H NMR (600 MHz, DMSO-*d*_6_): *δ* = 1.76 (s, 3H, CH_3_^T^), 2.07–2.17 (m, 2H, H-2′a + H-2′b), 4.24–4.31 (m, 2H, CH^Fmoc^ + H-4′), 4.35–4.41 (m, 3H, H-3′ + CH_2_^Fmoc^), 5.73 (d, *J* = 3.3 Hz, 1H, OH-3′), 6.38 (dd, *J* = 8.5, 6.2 Hz, 1H, H-1′), 7.34 (app. t, *J* = 6.9 Hz, 2H, H^Ar^), 7.43 (app. t, *J* = 7.5 Hz, 2H, H^Ar^), 7.73 (d, *J* = 6.9 Hz, 2H, H^Ar^), 7.90 (d, *J* = 7.5 Hz, 2H, H^Ar^), 8.04 (s, 1H, H-6), 9.42 (s, 1H, CO–NH), 10.19 (s, 1H, CO–NH), 11.33 (s, 1H, NH-3) ppm. ^13^C NMR (150 MHz, DMSO-*d*_6_): *δ* = 12.3 (CH_3_^T^), 38.5 (C-2′), 46.5 (CH^Fmoc^), 66.2 (CH_2_^Fmoc^), 73.6 (C-3′), 83.9 (C-4′), 85.2 (C-1′), 109.7 (C-5), 120.2 (CH^Ar^), 125.2 (CH^Ar^), 127.1 (CH^Ar^), 127.7 (CH^Ar^), 136.5 (C-6), 140.8 (C^Ar^), 143.6 (C^Ar^), 150.7 (C-2), 156.0 (CO^Fmoc^), 163.7 (C-4), 170.3 (C-5′) ppm. HRMS (APCI^+^): *m*/*z* calc. 493.1718 [M + H]^+^, found: 493.1717.

#### 5′-Deoxy-5′-hydrazino-5′-oxo-thymidine **31**

Fmoc-protected hydrazide **30** (135 mg, 0.27 mmol) was dissolved in DMF (0.8 mL). The reaction mixture was stirred at rt and piperidine (0.2 mL) was added. The reaction mixture was stirred at rt for 20 minutes. TLC analysis (CH_2_Cl_2_/MeOH; 4 : 1) indicated complete consumption of the starting material (*R*_f_ = 0.7) and the formation of product (*R*_f_ = 0.3). Toluene (5 mL) was added to the flask, followed by silica (∼0.5 g) and solvent was removed *in vacuo*. Purification by column chromatography (CH_2_Cl_2_/MeOH; 9 : 1) gave hydrazide **31** as a white powder (56 mg, 76%). M.p. 188–192 °C (decomp.). IR *ν*_max_ 3476, 3320, 3205, 3053, 2927, 1650, 1635, 1535, 1475, 1411, 1341, 1275, 1197, 1132, 1077, 1006, 960, 797 cm^–1^. ^1^H NMR (400 MHz, DMSO-*d*_6_): *δ* = 1.77 (d, *J* = 0.9 Hz, 3H, CH_3_^T^), 2.11 (ddd, *J* = 13.4, 5.6, 2.1 Hz, 1H, H-2′a), 2.21 (ddd, *J* = 13.4, 8.6, 5.0 Hz, 1H, H-2′b), 4.16 (d, *J* = 1.2 Hz, 1H, H-4′), 4.25–4.29 (m, 1H, H-3′), 4.39 (s (br), 2H, NH_2_), 5.65 (d, *J* = 4.3 Hz, 1H, OH-3′), 6.31 (dd, *J* = 8.6, 5.6 Hz, 1H, H-1′), 8.21 (d, *J* = 0.9 Hz, 1H, H-6), 9.54 (s, 1H, CO–NH), 11.28 (s, 1H, NH-3) ppm. ^13^C NMR (100 MHz, DMSO-*d*_6_): *δ* = 12.5 (CH_3_^T^), 38.9 (C-2′), 73.4 (C-3′), 84.3 (C-4′), 85.1 (C-1′), 109.5 (C-5), 136.9 (C-6), 150.6 (C-2), 163.8 (C-4), 169.3 (C-5′) ppm. HRMS (ESI^+^): *m*/*z* calc. 271.1037 [M + H]^+^, found: 271.1034.

#### 5′-Amino-5′-*N*-(2-diethylamino-3,4-dioxocyclobuten-1-yl)-5′-deoxythymidine **34**

Squaryl monoamide **33** (100 mg, 0.27 mmol) was dissolved in a solution of Et_2_NH (0.5 mL, 4.8 mmol) in MeCN (2.0 mL). The solution was heated to 55 °C and stirred for 1 hour. After this time, TLC analysis (EtOAc/MeOH; 9 : 1) showed complete consumption of the starting material (*R*_f_ = 0.3) and formation of the product (*R*_f_ = 0.1). The orange solution was concentrated to give the desired product **34** as a red solid (79 mg, 74%). M.p. 207–209 °C. IR *ν*_max_ 3438, 3213, 2978, 1790, 1712, 1651, 1559, 1517, 1442, 1275, 1227, 1083, 1063, 567 cm^–1^. ^1^H NMR (600 MHz, DMSO-*d*_6_): *δ* = 1.13 (t, *J* = 7.2 Hz, 6H, CH_3_^Et^), 1.78 (d, *J* = 1.0 Hz, 3H, CH_3_^T^), 2.04–2.11 (m, 2H, H-2′a + H-2′b), 3.45–3.60 (m, 4H, CH_2_^Et^), 3.73 (app. dt, *J* = 13.6, 5.0 Hz, 1H, H-5′a), 3.87 (ddd, *J* = 7.1, 5.0, 3.3 Hz, 1H, H-4′), 3.93 (app. dt, *J* = 13.6, 7.1 Hz, 1H, H-5′b), 4.19–4.22 (m, 1H, H-3′), 5.35 (d, *J* = 3.3 Hz, 1H, OH-3′), 6.14 (app. t, *J* = 7.1 Hz, 1H, H-1′), 7.42 (d, *J* = 1.0 Hz, 1H, H-6), 7.68–7.72 (m, 1H, NH^Sq^), 11.30 (s, 1H, NH-3) ppm. ^13^C NMR (150 MHz, DMSO-*d*_6_): *δ* = 12.0 (CH_3_^T^), 15.0 (CH_3_^Et^), 38.5 (C-2′), 43.5 (CH_2_^Et^), 45.5 (C-5′), 70.6 (C-3′), 83.9 (C-1′), 85.3 (C-4′), 109.7 (C-5), 135.8 (C-6), 150.4 (C-2), 163.7 (C-4), 166.9 (C^Sq1^), 167.0 (C^Sq2^), 181.8 (C^Sq4^), 182.5 (C^Sq3^) ppm. HRMS (ESI^+^): *m*/*z* calc. 393.1769 [M + H]^+^, found: 393.1756.

## Conflicts of interest

There are no conflicts to declare.

## Supplementary Material

Supplementary information
