# Supplementary material for: A hydroxamic-acid-containing nucleoside inhibits DNA repair nuclease SNM1A
Source: Org Biomol Chem. 2019 Aug 5;17(35):8094–105. doi: 10.1039/c9ob01133a (PMC6984127; doi:10.1039/c9ob01133a)
Supplement: Supplementary file 1 [file OB-017-C9OB01133A-s001.pdf]

Electronic Supplementary Material (ESI) for Organic & Biomolecular Chemistry.
This journal is © The Royal Society of Chemistry 2019

# Supporting Information

## A Hydroxamic-acid-containing nucleoside functions as an inhibitor of the DNA repair nuclease SNM1A

William Doherty,[a] Eva-Maria Dürr,[a] Hannah Baddock,[b] Sook Y. Lee,[b,c] Peter J. McHugh,[b] Tom Brown,[c] Mathias O. Senge,[d] Eoin M. Scanlan,[a] and Joanna F. McGouran *[a]

[a]*School of Chemistry and Trinity Biomedical Sciences Institute, Trinity College Dublin, 152-160 Pearse St., Dublin 2, Ireland. E-mail: jmcgoura@tcd.ie*

[b]*Department of Oncology, Weatherall Institute of Molecular Medicine, University of Oxford, John Radcliffe Hospital, Oxford OX3 9DS, UK.*

[c]*Department of Chemistry, University of Oxford, 12 Mansfield Road, Oxford OX1 3TA, UK.*

[d]*Molecular Medicine, Trinity Translational Medicine Institute, Trinity Centre for Health Sciences, Trinity College Dublin, The University of Dublin, St. James's Hospital, Dublin 8, Ireland.*

*Table of Contents*

**Experimental Details for Known Compounds ................................ S2**

**$^{1}$H and $^{13}$C NMR Spectra of Novel Compounds ................................ S15**

**Parallel Artificial Membrane Permeability Assay (PAMPA) .................... S37**

**References ............................................................ S39**

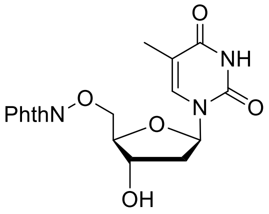

**S1**

**5'-*O*-Phthalimidothymidine[1] S1:** Under argon, thymidine **1** (5.09 g, 21.0 mmol) was dissolved in anhydrous DMF (55 mL), cooled to 0 °C and stirred. $PPh_3$ (7.04 g, 26.8 mmol) and *N*-hydroxyphthalimide (4.49 g, 27.5 mmol) were added followed by the dropwise addition of DIAD (6.1 mL, 31.1 mmol) in anhydrous DMF (10 mL). The reaction mixture was warmed to rt and stirred for 18 hours. After this time, TLC analysis ($CH_2Cl_2$/MeOH; 9:1) showed the complete consumption of the starting material ($R_f$ = 0.3) and the formation of the product ($R_f$ = 0.7). The solution was concentrated and the resulting oil dissolved in $CH_2Cl_2$ (50 mL). The solution was poured into a mixture of ice-$H_2O$ (250 mL) and stirred for 30 minutes. The resulting precipitate was collected by vacuum filtration and washed with cold EtOH (3 × 50 mL) to afford phthalimide **S1** as a white powder (6.11 g, 75%).

IR $\upsilon_{max}$ 3449, 3234, 3082, 2929, 1786, 1728, 1680, 1648 $cm^{-1}$.

$^1$H NMR (400 MHz, DMSO-$d_6$): δ = 1.80 (s, 3H, $CH_3^T$), 2.10-2.14 (m, 2H, H-2'a + H-2'b), 4.07-4.10 (m, 1H, H-4'), 4.36 (d, *J* = 4.4 Hz, 2H, H-5'a + H-5'b), 4.34-4.38 (m, 1H, H-3'), 5.46 (d, *J* = 3.8 Hz, 1H, OH), 6.19-6.23 (m, 1H, H-1'), 7.58 (s, 1H, H-6), 7.84-7.87 (m, 4H, Phth), 11.29 (s, 1 H, NH-3) ppm.

$^{13}$C NMR (100 MHz, DMSO-$d_6$): δ = 12.1 ($CH_3^T$), 38.8 (C-2'), 70.7 (C-3'), 77.7 (C-5'), 84.1 (C-4'), 84.3 (C-1'), 109.9 (C-5), 123.3 (Phth), 128.6 (qC, Phth), 134.8 (Phth), 135.8 (C-6), 150.4 (C-2), 163.0 (CO Phth), 163.7 (C-4) ppm.

HRMS (ESI$^+$): *m/z* calc. 410.0959 $[M + Na]^+$, found: 410.0958.

*Data are consistent with the literature.*[1]

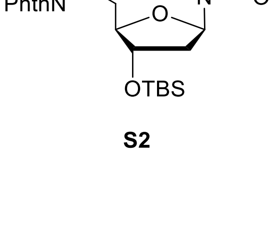

**S2**

**3'-*O*-(*tert*-Butyldimethylsilyl)-5'-*O*-phthalimidothymidine[1] S2:** Under argon, 5'-*O*-phthalimidothymidine **S1** (2.96 g, 7.7 mmol) was dissolved in anhydrous DMF (15 mL) and the reaction mixture was stirred. Imidazole (1.37 g, 20.1 mmol) was added followed by the dropwise addition of a solution of TBSCl (1.52 g, 10.1 mmol) in anhydrous DMF (15 mL) and the reaction mixture was stirred at rt for 18 hours. After this time, TLC analysis (EtOAc) showed the consumption of the starting material ($R_f = 0.3$) and the formation of the product ($R_f = 0.8$). The solution was diluted with brine (150 mL) and extracted with EtOAc (200 mL). The organic layer was washed with brine (2 × 50 mL), dried over $Na_2SO_4$, filtered and solvent was removed *in vacuo* to give the crude product. Purification by flash column chromatography (PE/EtOAc; 1:1) afforded silyl ether **S2** as a white powder (3.31 g, 86%).

IR $\upsilon_{max}$ 2930, 2856, 1790, 1701, 1665 $cm^{-1}$.

$^1H$ NMR (400 MHz, DMSO-$d_6$): δ = 0.12 (s, 6H, 2 × $CH_3^{TBS}$), 0.88 (s, 9H, $t$-$Bu^{TBS}$), 1.80 (d, $J$ = 0.9 Hz, 3H, $CH_3^T$), 2.09 (ddd, $J$ = 13.6, 6.1, 2.6 Hz, 1H, H-2'a), 2.25 (ddd, $J$ = 13.6, 8.0, 6.0 Hz, 1H, H-2'b), 4.07-4.10 (m, 1H, H-4'), 4.35-4.37 (m, 2H, H-5'a + H-5'b), 4.60-4.63 (m, 1H, H-3'), 6.19 (dd, $J$ = 8.0, 6.1 Hz, 1H, H-1'), 7.56 (d, $J$ = 0.9 Hz, 1H, H-6), 7.84-7.89 (m, 4H, Phth), 11.30 (s, 1 H, NH-3) ppm.

$^{13}C$ NMR (100 MHz, DMSO-$d_6$): δ = –4.94 ($CH_3^{TBS}$), –4.88 ($CH_3^{TBS}$), 12.1 ($CH_3^T$), 17.7 (qC, $t$-$Bu^{TBS}$), 25.7 ($t$-$Bu^{TBS}$), 39.0 (C-2'), 72.3 (C-3'), 77.1 (C-5'), 84.1 (C-4'), 84.3 (C-1'), 109.8 (C-5), 123.3 (Phth), 128.5 (qC, Phth), 134.8 (Phth), 135.8 (C-6), 150.4 (C-2), 162.9 (CO Phth), 163.6 (C-4) ppm.

HRMS ($ESI^+$): *m/z* calc. 524.1823 $[M + Na]^+$, found: 524.1825.

*Data are consistent with the literature.*[1]

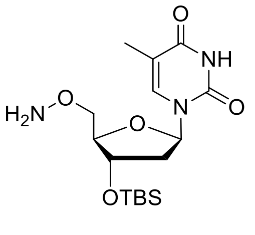


**2**

**5'-*O*-Amino-3'-*O*-(*tert*-butyldimethylsilyl)thymidine[1] 2:** Compound **S2** (2.00 g, 4.0 mmol) was suspended in MeOH (16 mL) and 80% hydrazine hydrate solution (0.75 mL, 12.3 mmol) was added. After 5 minutes, the reaction mixture became homogeneous, followed by the formation of a precipitate. After 3.5 h stirring at rt, TLC analysis (PE/EtOAc; 1:1) showed the complete consumption of the starting material ($R_f = 0.4$) and the formation of the product ($R_f = 0.1$). The suspension was

diluted with $Et_2O$ (80 mL) and washed with sat. aq. $NaHCO_3$ solution (80 mL). The aqueous layer was extracted with $Et_2O$ (3 x 150 mL), dried over $Na_2SO_4$, filtered and solvent was removed *in vacuo* to afford oxyamine **2** as a white solid (1.37 g, 90%). The crude product was carried forward without further purification and was used immediately in the next step.

IR $\upsilon_{max}$ 3222, 2930, 2858, 1662 $cm^{-1}$.

$^1H$ NMR (400 MHz, $CDCl_3$): δ = 0.08 (s, 6H, 2 × $CH_3^{TBS}$), 0.89 (s, 9H, *t*-$Bu^{TBS}$) 1.93 (d, *J* = 1.0 Hz, 3H, $CH_3^{T}$), 2.08 (app. dt, *J* = 13.4, 6.5 Hz, 1H, H-2'a), 2.26 (ddd, *J* = 13.4, 6.5, 3.7 Hz, 1H, H-2'b), 3.85 (dd, *J* = 11.0, 4.4 Hz, 1H, H-5'a), 3.96 (dd, *J* = 11.0, 3.1 Hz, 1H, H-5'b), 4.00-4.03 (m, 1H, H-4'), 4.37 (app. dt, *J* = 6.5, 3.7 Hz, 1H, H-3'), 6.24 (app. t, *J* = 6.5 Hz, 1H, H-1'), 7.38 (d, 1H, *J* = 1.0 Hz, H-6), 8.53 (s (br), 1 H, NH-3) ppm.

$^{13}C$ NMR (100 MHz, $CDCl_3$): δ = –4.7 ($CH_3^{TBS}$), –4.6 ($CH_3^{TBS}$), 12.9 ($CH_3^{T}$), 18.1 (qC, *t*-$Bu^{TBS}$), 25.8 (*t*-$Bu^{TBS}$), 41.1 (C-2'), 72.0 (C-3'), 75.5 (C-5'), 85.5 (C-1'), 85.6 (C-4'), 111.0 (C-5), 135.9 (C-6), 150.3 (C-2), 163.7 (C-4) ppm.

HRMS ($ESI^+$): *m/z* calc. 372.1949 $[M + H]^+$, found: 372.1940.

*Data are consistent with the literature.*[1]

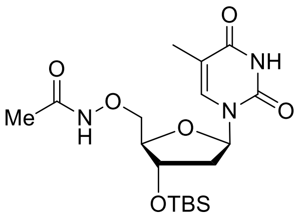


**3**

***N*-acetyl-5'-*O*-amino-3'-*O*-(*tert*-butyldimethylsilyl)thymidine[2] 3:** Under argon, AcOH (57 µl, 1.00 mmol) was dissolved in anhydrous DMF (10 mL), cooled to 0 °C and stirred. HOAt (166 mg, 1.22 mmol) and EDCI·HCl (213 mg, 1.11 mmol) were added and the reaction mixture was stirred for 10 minutes. Oxyamine **2** (350 mg, 0.94 mmol) was added and the reaction mixture was warmed to rt and stirred for 24 hours. After this time, TLC analysis ($CH_2Cl_2$/MeOH; 19:1) showed the consumption of the starting material ($R_f$ = 0.4) and the formation of the product ($R_f$ = 0.3). The reaction mixture was diluted with EtOAc (100 mL) and washed with sat. aq. $NaHCO_3$ solution (100 mL). The aqueous layer was extracted with EtOAc (2 × 100 mL) and the combined organic extracts were washed with brine (100 mL), dried over $Na_2SO_4$, filtered and solvent was removed *in vacuo* to give the crude product. Flash column chromatography ($CH_2Cl_2$/MeOH; 19:1) afforded the desired product **3** as a white foam (277 mg, 71%).

IR $\upsilon_{max}$ 3222, 2930, 2878, 1662 $cm^{-1}$.

$^{1}H$ NMR (400 MHz, $CDCl_3$): δ = 0.09 (s, 6H, 2 × $CH_3^{TBS}$), 0.89 (s, 9H, *t*-$Bu^{TBS}$), 1.92 (s, 3H, $CH_3^{Ac}$), 1.93 (s, 3H, $CH_3^{T}$), 2.20-2.30 (m, 2H, H-2'a + H-2'b), 4.01 (dd, *J* = 3.5, 2.7 Hz, 1H, H-4'), 4.07 (dd, *J* = 10.9, 3.5 Hz, 1H, H-5'a), 4.16 (app. d, *J* = 10.9 Hz, 1H, H-5'b), 4.59 (m, 1H, H-3'), 6.15 (app. t, *J* = 6.8 Hz, 1H, H-1'), 7.45 (s, 1H, H-6), 8.58-8.72 (m, 2H, NH-3 + O-NH) ppm.
$^{13}C$ NMR (100 MHz, $CDCl_3$): δ = –4.8 ($CH_3^{TBS}$), –4.6 ($CH_3^{TBS}$), 12.5 ($CH_3^{T}$), 18.0 (qC, *t*-$Bu^{TBS}$), 19.9 ($CH_3^{Ac}$), 25.8 (*t*-$Bu^{TBS}$), 40.5 (C-2'), 71.9 (C-3'), 75.3 (C-5'), 85.6 (C-4'), 86.5 (C-1'), 111.3 (C-5), 137.1 (C-6), 150.7 (C-2), 164.5 (C-4), 168.0 (CO-NH-O) ppm.

HRMS ($ESI^+$): *m/z* calc. 436.1874 $[M + Na]^+$, found: 436.1864.

*Data are consistent with the literature.*[2]

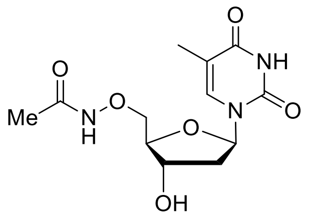


**5**

***N*-Acetyl-5'-*O*-aminothymidine**[2] **5:** Silyl ether **3** (270 mg, 0.65 mmol) was dissolved in THF (6.5 mL) and the mixture was stirred. TBAF·$3H_2O$ (370 mg, 1.17 mmol) was added and the resulting opaque mixture was stirred for 18 hours. After this time, TLC analysis ($CH_2Cl_2$/MeOH; 9:1) showed the complete consumption of the starting material ($R_f$ = 0.4) and the formation of the product ($R_f$ = 0.2). Silica (~ 1 g) was added to the flask and solvent was removed *in vacuo* to give the crude product. Purification by column chromatography ($CH_2Cl_2$/MeOH; 85:15) gave alcohol **5** as a white foam (180 mg, 92%).

IR $\upsilon_{max}$ 3263, 3191, 3022, 1647 $cm^{-1}$.

$^{1}H$ NMR (600 MHz, DMSO-$d_6$): δ = 1.73 (s, 3 H, $CH_3^{Ac}$), 1.80 (s, 3H, $CH_3^{T}$), 2.06-2.08 (m, 2H, H-2'a + H-2'b), 3.89 (dd, *J* = 10.0, 5.4 Hz, 1H, H-5'a), 3.92-3.94 (m, 1H, H-4'), 3.98 (dd, *J* = 10.0, 2.9 Hz, 1H, H-5'b), 4.26-4.28 (m, 1H, H-3'), 5.37 (s (br), 1H, OH), 6.19 (app. t, *J* = 7.0 Hz, 1H, H-1'), 7.65 (s, 1H, H-6), 10.86-11.54 (m, 2H, NH-3 + O-NH) ppm.

$^{13}C$ NMR (150 MHz, DMSO-$d_6$): δ = 12.1 ($CH_3^{T}$), 19.6 ($CH_3^{Ac}$), 39.1 (C-2'), 70.7 (C-3'), 75.7 (C-5'), 84.0 (C-4'), 84.1 (C-1'), 109.8 (C-5), 136.1 (C-6), 150.5 (C-2), 163.8 (C-4), 166.6 (CO-NH-O) ppm.

HRMS ($ESI^+$): *m/z* calc. 322.1010 $[M + Na]^+$, found: 322.1011.

*Data are consistent with the literature.*[2]

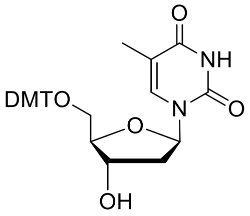


**S3**

**5'-*O*-(4,4'-Dimethoxytrityl)thymidine[3] S3:** Under argon, thymidine **1** (10.0 g, 41.3 mmol) was dissolved in pyridine. 4,4'-dimethoxytrityl chloride (14.9 g, 44.0 mmol) was added in four portions over 1 h and the reaction mixture was stirred at rt for 20 hours. After this time, TLC analysis (EtOAc) showed the consumption of starting material ($R_f$ = 0.1) and the formation of product ($R_f$ = 0.5). The mixture was diluted with sat. aq. $NaHCO_3$ solution (250 mL), $CH_2Cl_2$ (500 mL) and $H_2O$ (500 mL). The layers were separated and the aqueous layer was extracted with $CH_2Cl_2$ (2 × 250 mL). The combined organic extracts were washed with $H_2O$ (2 × 500 mL) and brine (500 mL), dried over $MgSO_4$, filtered and concentrated. The residue was dissolved in EtOAc (50 mL) and poured into petroleum ether (1 L). The precipitate was collected by vacuum filtration and recrystallised from $CH_2Cl_2$ to afford the desired product **S3** as a white crystalline solid (17.0 g, 76%).

M.p. ($CH_2Cl_2$) 138-140 °C; lit.[3] 122-124 °C.

IR $\upsilon_{max}$ 3346, 2970, 2837, 1693, 1669, 1607, 1508, 1469, 1250, 1175, 1109, 1094, 1033, 962, 826, 735, 700 $cm^{-1}$.

$^1$H NMR (400 MHz, DMSO-$d_6$): δ = 1.45 (d, *J* = 0.8 Hz, 3H, $CH_3^T$), 2.15 (ddd, *J* = 13.4, 6.3, 3.7 Hz, 1H, H-2'a), 2.25 (app. dt, *J* = 13.4, 6.8 Hz, 1H, H-2'b), 3.17 (dd, *J* = 10.5, 3.1 Hz, 1H, H-5'a), 3.21 (dd, *J* = 10.5, 4.2 Hz, 1 H, H-5'b), 3.79 (s, 6H, 2 × $OCH_3$), 3.88 (ddd, *J* = 4.2, 3.5, 3.1 Hz, 1H, H-4'), 4.32 (m, 1H, H-3'), 5.32 (d, *J* = 2.9 Hz, 1H, OH), 6.20 (app. t, *J* = 6.8 Hz, 1H, H-1'), 6.82-6.84 (m, 4H, $H^{Ar}$), 7.22-7.26 (m, 1H, $H^{Ar}$), 7.29-7.32 (m, 6H, $H^{Ar}$), 7.40-7.42 (m, 2H, $H^{Ar}$), 7.50 (d, *J* = 0.9 Hz, 1H, H-6), 11.33 (s, 1H, NH-3) ppm.

$^{13}$C NMR (150 MHz, DMSO-$d_6$): δ = 11.7 ($CH_3^T$), 39.5 (C-2'), 55.1 ($OCH_3$), 63.8 (C-5'), 70.6 (C-3'), 83.8 (C-1'), 85.5 (C-4'), 85.9 (DMT), 109.6 (C-5), 113.3 (DMT), 126.8 (DMT), 127.7 (DMT), 127.9 (DMT), 129.8 (DMT), 135.3 (DMT), 135.5 (DMT), 135.7 (C-6), 144.7 (DMT), 150.4 (C-2), 158.16 (DMT), 158.18 (DMTr), 163.7 (C-4) ppm.

HRMS (ESI$^+$): *m/z* calc. 567.2102 $[M + Na]^+$, found: 567.2093.

*Data are consistent with the literature.*[4]

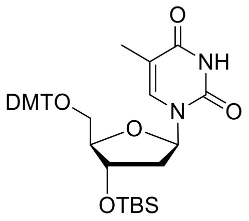


**S4**

**5'-*O*-(4,4'-Dimethoxytrityl)-3'-*O*-(*tert*-butyldimethylsilyl)thymidine[5] S4:** Under argon, protected thymidine **S3** (17.0 g, 31.2 mmol) and imidazole (6.38 g, 93.7 mmol) were dissolved in anhydrous DMF (100 mL) and the mixture was stirred. A solution of TBSCl (6.12 g, 40.6 mmol) in anhydrous DMF (60 mL) was added dropwise and the reaction mixture was stirred at rt for 16 hours. After this time, TLC analysis (EtOAc) showed the complete consumption of starting material ($R_f = 0.5$) and the formation of product ($R_f = 0.8$). $H_2O$ (200 mL) was added and the aqueous layer was extracted with $Et_2O$ (2 × 200 mL). The combined organic extracts were washed with brine (100 mL), dried over $Na_2SO_4$, filtered and solvent was removed *in vacuo* to give the crude product. Purification by flash column chromatography (PE/EtOAc; 2:1 → EtOAc) afforded the desired product **S4** as a white foam (17.6 g, 85%).

$^1$H NMR (400 MHz, $CDCl_3$): δ = –0.03 (s, 3H, $CH_3^{TBS}$), 0.02 (s, 3H, $CH_3^{TBS}$), 0.83 (s, 9H, *t*-Bu$^{TBS}$), 1.49 (d, *J* = 1.0 Hz, 3H, $CH_3^{T}$), 2.22 (app. dt, *J* = 13.3, 6.7 Hz, 1H, H-2'a), 2.33 (ddd, *J* = 13.3, 6.1, 3.5 Hz, 1H, H-2'b), 3.26 (dd, *J* = 10.6, 2.8 Hz, 1H, H-5'a), 3.47 (dd, *J* = 10.6, 2.8 Hz, 1H, H-5'b), 3.79 (s, 6H, 2 × $OCH_3$), 3.96 (app. q, *J* = 2.8 Hz, 1H, H-4'), 4.52 (app. dt, *J* = 6.4, 3.5 Hz, 1H, H-3'), 6.35 (app. t, *J* = 6.6 Hz, 1H, H-1'), 6.82-6.84 (m, 4H, $H^{Ar}$), 7.22-7.26 (m, 1H, $H^{Ar}$), 7.29-7.32 (m, 6H, $H^{Ar}$), 7.40-7.42 (m, 2H, $H^{Ar}$), 7.65 (d, *J* = 1.0 Hz, 1H, H-6), 8.52 (s, 1H, NH-3) ppm.

$^{13}$C NMR (100 MHz, $CDCl_3$): δ = –4.7 ($CH_3^{TBS}$), –4.5 ($CH_3^{TBS}$), 12.0 ($CH_3^{T}$), 18.1 (qC, *t*-Bu$^{TBS}$), 25.9 (*t*-Bu$^{TBS}$), 41.7 (C-2'), 55.4 ($OCH_3$), 63.0 (C-5'), 72.2 (C-3'), 85.0 (C-1'), 86.9 (C-4'), 87.0 (DMT), 111.2 (C-5), 113.38 (DMT), 113.41 (DMT), 127.3 (DMT), 128.1 (DMT), 128.3 (DMT), 130.17 (DMT), 130.20 (DMT), 135.5 (DMT), 135.6 (DMT), 135.8 (C-6), 144.5 (DMT), 150.3 (C-2), 158.9 (DMT), 163.8 (C-4) ppm.

HRMS (ESI$^+$): *m/z* calc. 681.2966 $[M + Na]^+$, found: 681.2969

*Data are consistent with the literature.*[5]

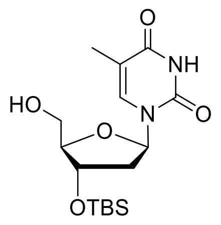

**7**

**3'-*O*-(*tert*-Butyldimethylsilyl)thymidine[6] 7:** Dimethyoxytrityl ether **S4** (5.00 g, 7.59 mmol) was dissolved in 80% AcOH in $H_2O$ (100 mL) and the resulting clear orange reaction mixture stirred at rt for 45 minutes. After this time, TLC analysis ($CH_2Cl_2$/MeOH; 19:1) showed the complete consumption of starting material ($R_f$ = 0.5) and the formation of product ($R_f$ = 0.1). The reaction mixture was carefully quenched with sat. aq. $K_2CO_3$ solution (~ 150 mL) until the pH was ~7. The mixture was extracted with EtOAc (4 × 100 mL) and the combined organic layers were dried over $MgSO_4$, filtered and solvent was removed *in vacuo* to give the crude product as a yellow oil. Purification by column chromatography ($CH_2Cl_2$/MeOH; 98:2) gave alcohol **7** as a white foam (2.26 g, 84%).

$^1$H NMR (400 MHz, $CDCl_3$): δ = 0.08 (s, 6H, 2 × $CH_3^{TBS}$), 0.89 (s, 9H, *t*-Bu$^{TBS}$), 1.91 (d, *J* = 1.2 Hz, 3H, $CH_3^{T}$), 2.21 (ddd, *J* = 13.5, 6.5, 3.7 Hz, 1H, H-2'a), 2.36 (app. dt, *J* = 13.5, 6.8 Hz, 1H, H-2'b), 3.75 (dd, *J* = 12.6, 3.6 Hz, 1H, H-5'a), 3.89-3.95 (m, 2H, H-5'b + H-4'), 4.49 (app. dt, *J* = 6.5, 3.7 Hz, 1H, H-3'), 6.13 (app. t, *J* = 6.8 Hz, 1H, H-1'), 7.35 (d, *J* = 1.2 Hz, 1H, H-6), 8.56 (s, 1H, NH-3) ppm.

*Data are consistent with the literature.[6]*

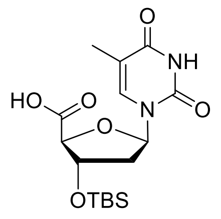

**8**

**5'-Oxo-3'-*O*-(*tert*-butyldimethylsilyl)thymidine[7] 8:** Alcohol **7** (2.23 g, 6.26 mmol) was suspended in MeCN/$H_2O$; 1:1 (27 mL) and the reaction mixture was stirred. BAIB (4.85 g, 15.06 mmol) followed by TEMPO (286 mg, 1.83 mmol) were added sequentially and the resulting homogeneous orange solution was stirred at rt for 2 hours. After this time, TLC analysis (EtOAc/MeOH; 4:1) showed the complete consumption of starting material ($R_f$ = 0.7) and the formation of product ($R_f$ = 0.2). 0.5 M aqueous KOH solution (100 mL) was gradually added to the flask and the mixture was stirred for 20 minutes. The mixture was extracted with $CH_2Cl_2$ (3 × 25 mL) and the combined organic layers were washed with $H_2O$ (25 mL). The combined aqueous layers were cooled in ice/$H_2O$ and were carefully acidified *via* dropwise addition of 2 M aqueous HCl solution until the pH was ~2.

The resulting precipitate was filtered and the residue was washed with ice-cold $H_2O$ (20 mL). The product was dried further under high vacuum to give carboxylic acid **8** as a white powder (2.00 g, 86%).

$^1$H NMR (400 MHz, DMSO-$d_6$): δ = 0.12 (s, 6H, 2 × $CH_3^{TBS}$), 0.89 (s, 9H, *t*-$Bu^{TBS}$), 1.78 (s, 3H, $CH_3^{T}$), 2.01-2.17 (m, 2H, H-2'a + H-2'b), 4.27-4.31 (m, 1H, H-4'), 4.60-4.69 (m, 1H, H-3'), 6.30 (dd, *J* = 8.4, 6.0 Hz, 1H, H-1'), 8.02 (s, 1H, H-6), 11.34 (s, 1H, NH-3), 13.33 (s, 1H, $CO_2H$) ppm.

M.p. 155-160 °C (decomp.).

*Data are consistent with the literature.*[7]

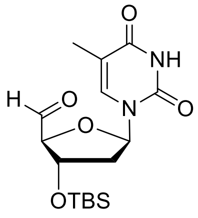


**12**

**5'-Oxo-3'-*O*-(*tert*-butyldimethylsilyl)-5'-deoxythymidine**[8] **12:** Under argon, alcohol **7** (360 mg, 1.01 mmol) was dissolved in anhydrous $CH_2Cl_2$ (10 mL). The mixture was cooled to 0 °C and stirred. The Dess-Martin periodinane (640 mg, 1.51 mmol) was added and the reaction mixture was stirred at 0 °C for 15 minutes and 4 hours at rt. After this time, TLC analysis ($CH_2Cl_2$/MeOH; 19:1) showed the complete consumption of starting material ($R_f$ = 0.5). *The aldehyde product is unstable on silica gel.* The resulting white suspension was diluted with $Et_2O$ (30 mL) and poured onto a mixture of sat. aq. $NaHCO_3$ solution (30 mL) and 25% w/v sodium thiosulfate solution (10 mL) and mixed for 5 minutes. The mixture was transferred to a separating funnel and the layers were separated. The ethereal layer was washed with sat. aq. $NaHCO_3$ solution (10 mL), brine (10 mL), dried over $Na_2SO_4$, filtered and solvent was removed *in vacuo* to give aldehyde **12** as a white foam which was used immediately in the next step without further purification (346 mg, 97%).

$^1$H NMR (400 MHz, $CDCl_3$): δ = 0.09 (s, 3H, $CH_3^{TBS}$), 0.14 (s, 3H, $CH_3^{TBS}$), 0.92 (s, 9H, *t*-$Bu^{TBS}$), 1.97 (d, *J* = 1.1 Hz, 3H, $CH_3^{T}$), 2.01-2.08 (m, 1H, H-2'a), 2.32 (ddd, *J* = 13.2, 5.8, 1.9 Hz, 1H, H-2'b), 4.48 (d, *J* = 1.9 Hz, 1H, H-4'), 4.66-4.69 (m, 1H, H-3'), 6.32 (dd, *J* = 8.1, 5.8 Hz, 1H, H-1'), 7.57 (d, *J* = 1.1 Hz, 1H, H-6), 8.28 (s, 1H, NH-3), 9.76 (s, 1H, CHO) ppm. *Note: aldehyde **12** appears oligomeric in solution by NMR spectroscopy. Therefore, only the major species is reported.*

*Data are consistent with the literature.*[8]

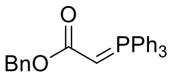


**18**

**Benzyl 2-(triphenylphosphoranylidene)acetate[9] 18:** Triphenylphosphine (2.62 g, 10.0 mmol) was dissolved in toluene (12 mL) and the mixture was stirred. A toluene solution (6 mL) of benzyl bromoacetate (1.58 mL, 10.0 mmol) was added in a dropwise fashion over 2 hours and the reaction mixture was stirred overnight at room temperature. The resulting phosphonium salt was filtered and washed with toluene (10 mL) followed by hexane (10 mL). The salt was dissolved in $CH_2Cl_2$ (9 mL) and the mixture was stirred vigorously. A solution of NaOH (400 mg, 10.0 mmol) in water (3 mL) was added in a dropwise fashion over 1 hour and the biphasic reaction mixture was stirred at room temperature for 1 hour. After this time, TLC analysis ($CH_2Cl_2$/MeOH; 9:1) indicated the formation of product ($R_f$ = 0.2). The layers were separated and the aqueous layer was extracted with $CH_2Cl_2$ (3 × 5 mL). The combined organic layers were dried over $MgSO_4$, filtered and solvent was removed *in vacuo* to give a crude oil. Recrystallisation from toluene/hexane gave ylide **18** as colourless crystals (2.46 g, 60%).

$^1$H NMR (400 MHz, $CDCl_3$): δ = 2.96 (s (br), 1H, P-CH), 5.00 (s, 2H, $H^{benzyl}$), 7.14-7.31 (m, 3H, $H^{Ar}$), 7.38-7.48 (m, 6H, $H^{Ar}$), 7.50-7.57 (m, 3H, $H^{Ar}$), 7.58-7.69 (m, 6, $H^{Ar}$) ppm.

$^{31}$P NMR (162 MHz, $CDCl_3$): δ = 17.9 ppm.

*Data are consistent with the literature.*[9]

FmocHN–$NH_2$

**S5**

**9-Fluorenylmethyl carbazate[10] S5:** Fmoc-OSu (2.01 g, 5.33 mmol) was dissolved in MeCN (30 mL) and the mixture was stirred. 80% hydrazine monohydrate solution in $H_2O$ (325 µL, 5.33 mmol) in MeCN (15 mL) was added to the mixture in a dropwise fashion *via* dropping funnel over 40 minutes. The resulting white suspension was stirred overnight at rt. TLC analysis (EtOAc) indicated substantial consumption of the starting material ($R_f$ = 0.6) and the formation of product ($R_f$ = 0.4). The reaction mixture was filtered and the residue was washed with MeCN/$Et_2O$; 1:1 (30 mL) followed by $Et_2O$ (30 mL) which gave hydrazide **S5** as white powder (618 mg, 46%).

$^1$H NMR (400 MHz, $CDCl_3$): δ = 3.74 (s (br), 2H, $NH_2$), 4.17-4.30 (m, 1H, $CH^{Fmoc}$), 4.38-4.51 (m, 2H, $CH_2^{Fmoc}$), 6.02 (s (br), 1H, CO-NH), 7.32 (app. t, $J$ = 7.5 Hz, 2H, $H^{Ar}$), 7.40 (d, $J$ = 7.5 Hz, 2H, $H^{Ar}$), 7.57 (d, $J$ = 7.5 Hz, 2H, $H^{Ar}$), 7.77 (d, $J$ = 7.5 Hz, 2H, $H^{Ar}$) ppm.

*Data are consistent with the literature.*[10]

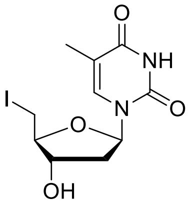

**S6**

**5'-Deoxy-5'-iodothymidine[11] S6:** Under argon, thymidine **1** (20.0 g, 82.6 mmol) was suspended in anhydrous THF (300 mL) and the reaction mixture was stirred. $PPh_3$ (26.0 g, 99.1 mmol) and imidazole (7.0 g, 102.8 mmol) were added and the reaction mixture was cooled to 0 °C. A solution of iodine (23.0 g, 90.6 mmol) in anhydrous THF (100 mL) was added dropwise and the reaction mixture was slowly warmed to rt and stirred for 18 hours. After this time, TLC analysis ($CH_2Cl_2$/MeOH; 9:1) showed the complete consumption of the starting material ($R_f$ = 0.2) and the formation of the product ($R_f$ = 0.4). The reaction was quenched by the addition of $H_2O$ (100 mL). THF was removed *in vacuo* and EtOAc (300 mL) was added. The crude product was collected as a white precipitate by vacuum filtration. The layers of the filtrate were separated and the aqueous layer was extracted with EtOAc (4 x 100 mL). The combined organic extracts were concentrated to approx. 100 mL to afford more product as a white precipitate which was collected by vacuum filtration. The combined precipitates were recrystallised from EtOH to give iodide **S6** as a white crystalline solid (23.0 g, 81%).

M.p. (EtOH) 166-168 °C (decomp.); lit.[11] 180-183 °C.

IR $\upsilon_{max}$ 3462, 3145, 3018, 2816, 1698, 1665 $cm^{-1}$.

$^1$H NMR (600 MHz, DMSO-$d_6$): δ = 1.80 (s, 3H, $CH_3^T$), 2.08 (ddd, *J* = 13.5, 6.4, 3.1 Hz, 1H, H-2'a), 2.29 (ddd, *J* = 13.5, 7.8, 6.4 Hz, 1H, H-2'b), 3.39 (dd, *J* = 10.4, 6.2 Hz, 1H, H-5'a), 3.52 (dd, *J* = 10.4, 6.2 Hz, 1H, H-5'b), 3.81 (app. td, *J* = 6.2, 3.0 Hz, 1H, H-4'), 4.19 (app. dt, *J* = 6.4, 3.0 Hz, 1H, H-3'), 5.49 (s (br), 1H, OH-3'), 6.22 (dd, *J* = 7.8, 6.4 Hz, 1H, H-1'), 7.52 (s, 1H, H-6), 11.33 (s, 1H, NH-3) ppm.

$^{13}$C NMR (150 MHz, DMSO-$d_6$): δ = 7.9 (C-5'), 12.2 ($CH_3^T$), 37.9 (C-2'), 73.0 (C-3'), 84.0 (C-1'), 85.4 (C-4'), 109.9 (C-5), 136.2 (C-6), 150.5 (C-2), 163.7 (C-4) ppm.

HRMS ($APCI^+$): *m/z* calc. 352.9998 $[M + H]^+$, found: 352.9992

*Data are consistent with the literature.*[11]

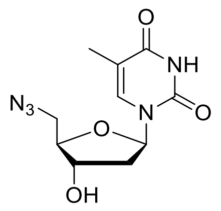

**S7**

**5'-Deoxy-5'-azidothymidine[12] S7:** Under argon, iodide **S6** (3.52 g, 10.0 mmol) was dissolved in anhydrous DMF (50 mL) and the mixture was stirred. After $NaN_3$ (2.04 g, 31.4 mmol) was added, the reaction mixture was heated to 60 °C and stirred for 17 hours. After this time, TLC analysis ($CH_2Cl_2$/MeOH; 9:1) showed the complete consumption of the starting material ($R_f$ = 0.6) and the formation of the product ($R_f$ = 0.5). The reaction mixture was cooled to rt, diluted with $H_2O$ (30 mL) and extracted with EtOAc (12 × 50 mL). The combined organic layers were washed with brine (50 mL), dried over $MgSO_4$, filtered and solvent was removed *in vacuo*. The residue was recrystallised from MeOH to afford azide **S7** as a white crystalline solid (2.39 g, 90%).

M.p. (MeOH) 156-157 °C; lit.[12] 157-159 °C.

IR $\upsilon_{max}$ 3382, 3184, 3049, 2928, 2094 , 1716, 1649 $cm^{-1}$.

$^1$H NMR (600 MHz, DMSO-$d_6$): δ = 1.79 (d, $J$ = 1.2 Hz, 3H, $CH_3^T$), 2.08 (ddd, $J$ = 13.6, 6.6, 3.7 Hz, 1H, H-2'a), 2.25 (app. dt, $J$ = 13.6, 7.0 Hz, 1H, H-2'b), 3.55 (d, $J$ = 5.1 Hz, 2H, H-5'), 3.84 (app. dt, $J$ = 5.1, 3.7 Hz, 1H, H-4'), 4.18-4.21 (m, 1H, H-3'), 5.40 (d, $J$ = 4.4 Hz, 1H, OH-3'), 6.20 (dd, $J$ = 7.0, 6.6 Hz, 1H, H-1'), 7.49 (d, $J$ = 1.2 Hz, 1H, H-6), 11.32 (s, 1H, NH-3) ppm.

$^{13}$C NMR (150 MHz, DMSO-$d_6$): δ = 12.1 ($CH_3^T$), 38.1 (C-2'), 51.7 (C-5'), 70.7 (C-3'), 83.9 (C-1'), 84.6 (C-4'), 109.8 (C-5), 136.1 (C-6), 150.5 (C-2), 163.7 (C-4) ppm.

HRMS (APCI$^-$): *m/z* calc. 266.0894 $[M – H]^-$, found: 266.0904.

*Data are consistent with the literature.*[13]

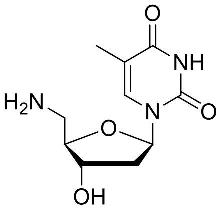

**32**

**5'-Deoxy-5'-aminothymidine[12] 32:** Under argon, azide **S7** (1.68 g, 6.3 mmol) was dissolved in THF (45 mL) and the mixture was stirred. After $PPh_3$ (3.50 g, 13.4 mmol) was added, the reaction mixture was stirred at rt for 10 minutes before $H_2O$ (15 mL) was added. After stirring at rt for 4 h, TLC

analysis showed the complete consumption of the starting material [EtOAc/MeOH; 9:1 ($R_f$ = 0.7)] and the formation of the product [($H_2O$/*i*-PrOH/EtOAc; 1:2:2 ($R_f$ = 0.2)]. THF was removed *in vacuo* and the remaining aqueous mixture was washed with $CH_2Cl_2$ (5 × 30 mL). The combined organic layer was extracted with $H_2O$ (50 mL) and the combined aqueous layers were concentrated *in vacuo* to afford the desired product **32** as a white solid (1.47 g, 97%). The crude product was carried forward without further purification.

IR $\upsilon_{max}$ 3349, 3315, 2930, 1658, 1605 $cm^{-1}$.

$^1H$ NMR (400 MHz, DMSO-$d_6$): δ = 1.78 (d, *J* = 1.0 Hz, 3H, $CH_3^T$), 2.03 (ddd, *J* = 13.4, 6.3, 3.5 Hz, 1H, H-2'a), 2.13 (ddd, *J* = 13.4, 7.5, 6.4 Hz, 1H, H-2'b), 2.68-2.76 (m, 2H, H-5'), 3.64 (app. dt, *J* = 5.2, 3.4 Hz, H, H-4'), 4.19 (app. dt, *J* = 6.4, 3.5 Hz, 1H, H-3'), 5.16 (s (br), 3 H, $NH_2$ + OH-3'), 6.13 (dd, *J* = 7.5, 6.3 Hz, 1H, H-1'), 7.64 (d, *J* = 1.0 Hz, 1H, H-6) ppm.

$^{13}C$ NMR (100 MHz, DMSO-$d_6$): δ = 12.6 ($CH_3^T$), 39.3 (C-2'), 44.1 (C-5'), 71.2 (C-3'), 83.8 (C-1'), 88.4 (C-4'), 110.0 (C-5), 136.7 (C-6), 151.0 (C-2), 164.2 (C-4) ppm.

HRMS (ESI$^+$): *m/z* calc. 242.1135 $[M + H]^+$, found: 242.1137.

*Data are consistent with the literature.*[12]

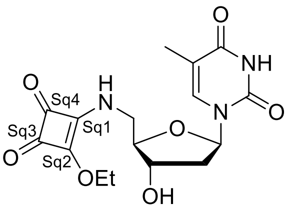


**33**

**5'-Amino-5'-*N*-(2-ethoxy-3,4-dioxocyclobuten-1-yl)-5'-deoxythymidine[2] 33:** Amine **32** (100 mg, 0.41 mmol) was dissolved in DMF (4 mL). DIPEA (36 µl, 0.21 mmol) and diethyl squarate (61 µl, 0.41 mmol) were added and the reaction mixture was stirred at rt for 24 hours. After this time, TLC analysis showed the consumption of the starting material [$H_2O$/*i*-PrOH/EtOAc; 1:2:2 ($R_f$ = 0.2)] and the formation of the product [$CH_2Cl_2$/MeOH; 9:1 ($R_f$ = 0.3)]. The reaction mixture was concentrated and purified by flash column chromatography (EtOAc/MeOH; 9:1) to afford the desired product **20** as a yellow solid (93 mg, 61%).

IR $\upsilon_{max}$ 3450, 3313, 3191, 3053, 2927, 1804, 1685, 1580 $cm^{-1}$.

$^1H$ NMR (400 MHz, DMSO-$d_6$): δ = 1.32 (t, *J* = 7.0 Hz, 1.5H, $CH_3^{Et}$), 1.37 (t, *J* = 7.0 Hz, 1.5H, $CH_3^{Et}$), 1.76 (s, 1.5H, $CH_3^T$), 1.78 (s, 1.5H, $CH_3^T$) 2.04-2.10 (m, 1H, H-2'a), 2.12-2.22 (m, 1H, H-2'b), 3.44-3.48 (m, 0.5H, H-5'a), 3.55-3.58 (m, 0.5H, H-5'a), 3.70-3.73 (m 1H, H-5'b), 3.76-3.82

(m, 1H, H-4'), 4.16-4.20 (m, 0.5H, H-3'), 4.22-4.26 (m, 0.5H, H-3'), 4.56-4.68 (m, 2H, $CH_2^{Et}$), 5.37 (d, *J* = 4.6 Hz, 0.5 H, OH-3'), 5.38 (d, *J* = 4.6 Hz, 0.5H, OH-3'), 6.13-6.16 (m, 1H, H-1'), 7.33 (s, 0.5H, H-6), 7.36 (s, 0.5H, H-6), 8.75 (t, *J* = 5.6 Hz, 0.5 H, $NH^{Sq}$), 8.95 (t, *J* = 5.6 Hz, 0.5H, $NH^{Sq}$), 11.31 (s (br), 1H, NH-3) ppm.

$^{13}C$ NMR (100 MHz, DMSO-$d_6$): δ = 12.0 ($CH_3^T$), 12.1 ($CH_3^T$), 15.6 ($CH_3^{Et}$), 38.1 (C-2'), 38.4 (C-2'), 45.7 (C-5'), 68.86 ($CH_2^{Et}$), 68.90 ($CH_2^{Et}$), 70.5 (C-3'), 70.6 (C-3'), 83.7 (C-1'), 83.8 (C-1'), 84.7 (C-4'), 84.9 (C-4'), 109.7 (C-5), 109.8 (C-5), 135.9 (C-6), 136.0 (C-6), 150.4 (C-2), 163.7 (C-4), 172.5 ($C^{Sq1}$), 173.2 ($C^{Sq1}$), 176.8 ($C^{Sq2}$), 177.3 ($C^{Sq2}$), 182.1 ($C^{Sq3}$), 182.4 ($C^{Sq3}$), 189.1 ($C^{Sq4}$), 189.4 ($C^{Sq4}$), ppm. *Note: compound **33** exhibits rotamers in NMR spectroscopy.*

HRMS (APCI$^+$): *m/z* calc. 366.1296 $[M + H]^+$, found: 366.1290.

*Data are consistent with the literature*.[2]

$^{1}H$ (400 MHz, $CDCl_3$) and $^{13}C$ (100 MHz, $CDCl_3$) NMR spectra of **4**

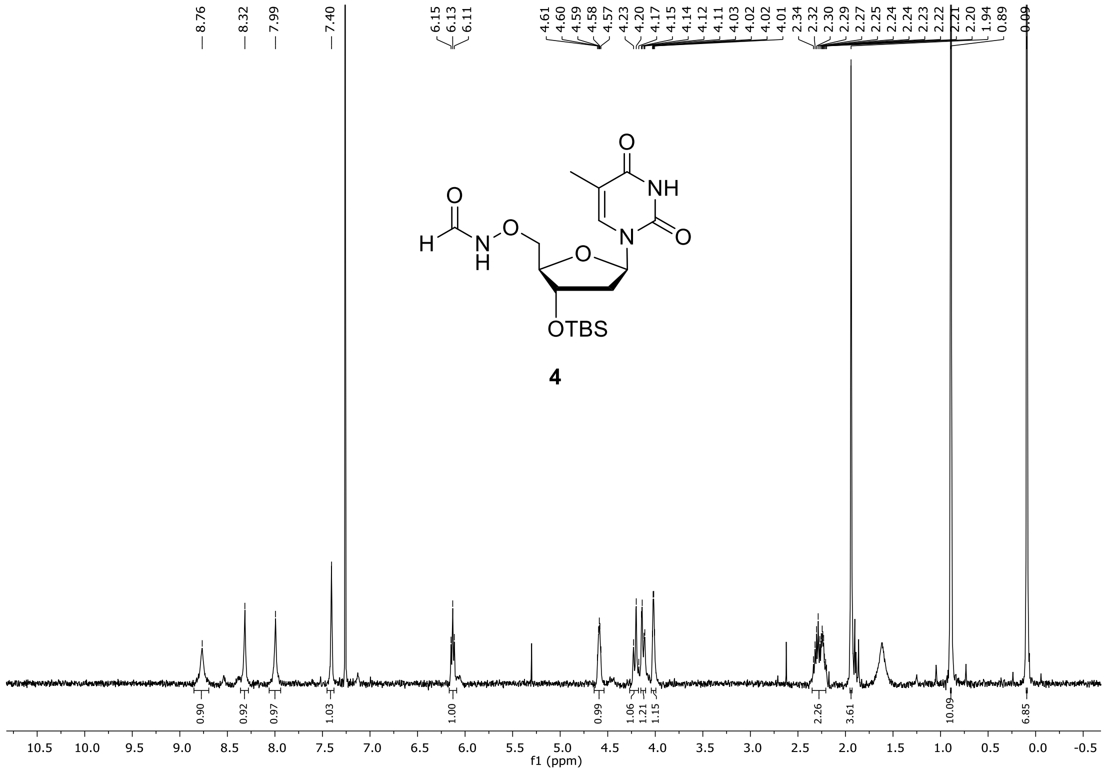


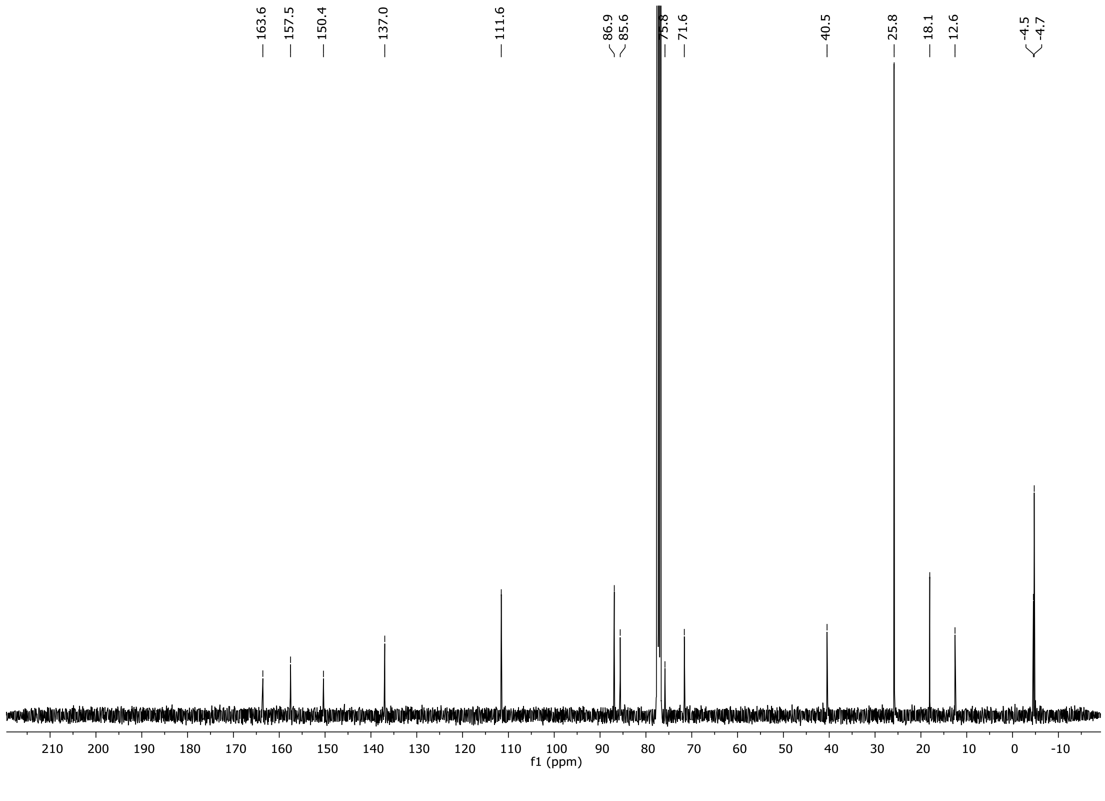

$^{1}$H (600 MHz, DMSO-$d_6$) and $^{13}$C (150 MHz, DMSO-$d_6$) NMR spectra of **6**

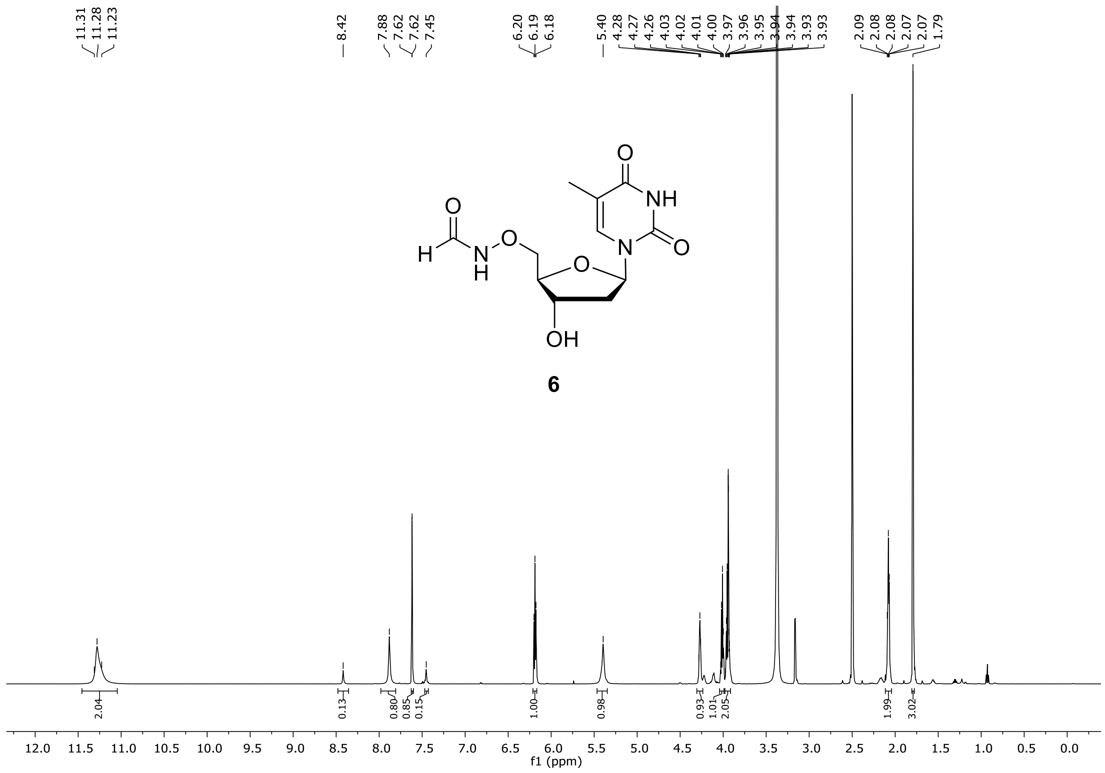


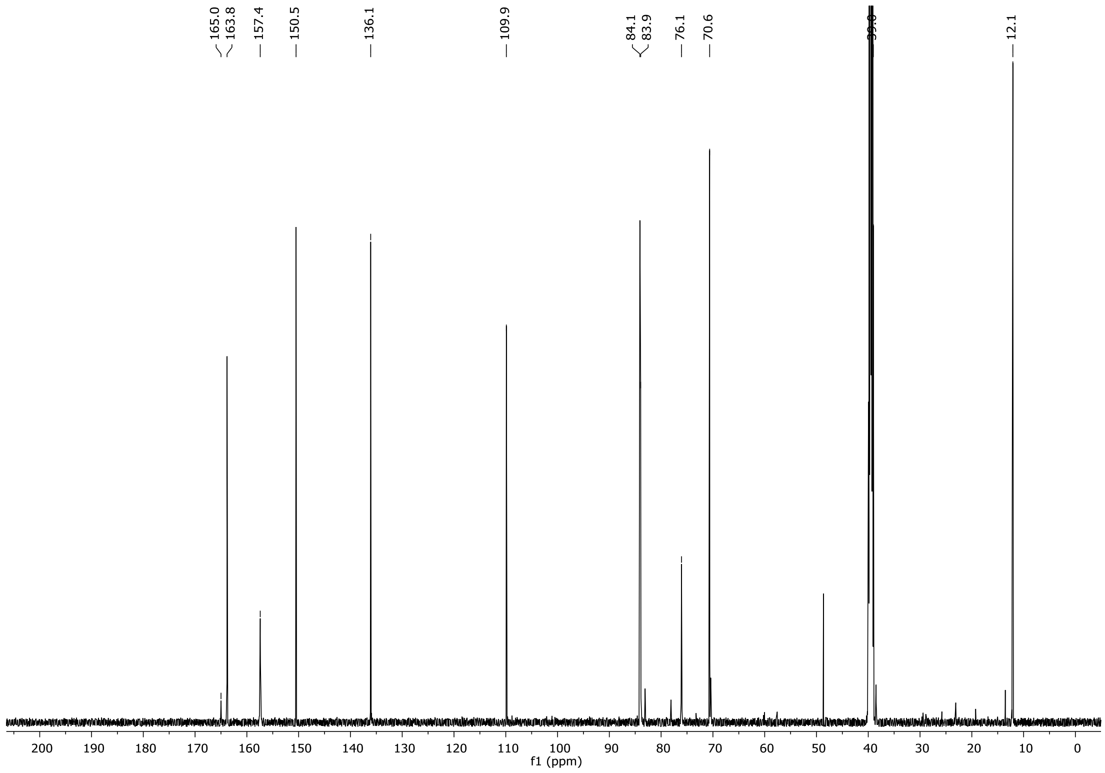

$^{1}$H (400 MHz, $CDCl_3$) and $^{13}$C (100 MHz, $CDCl_3$) NMR spectra of **9**

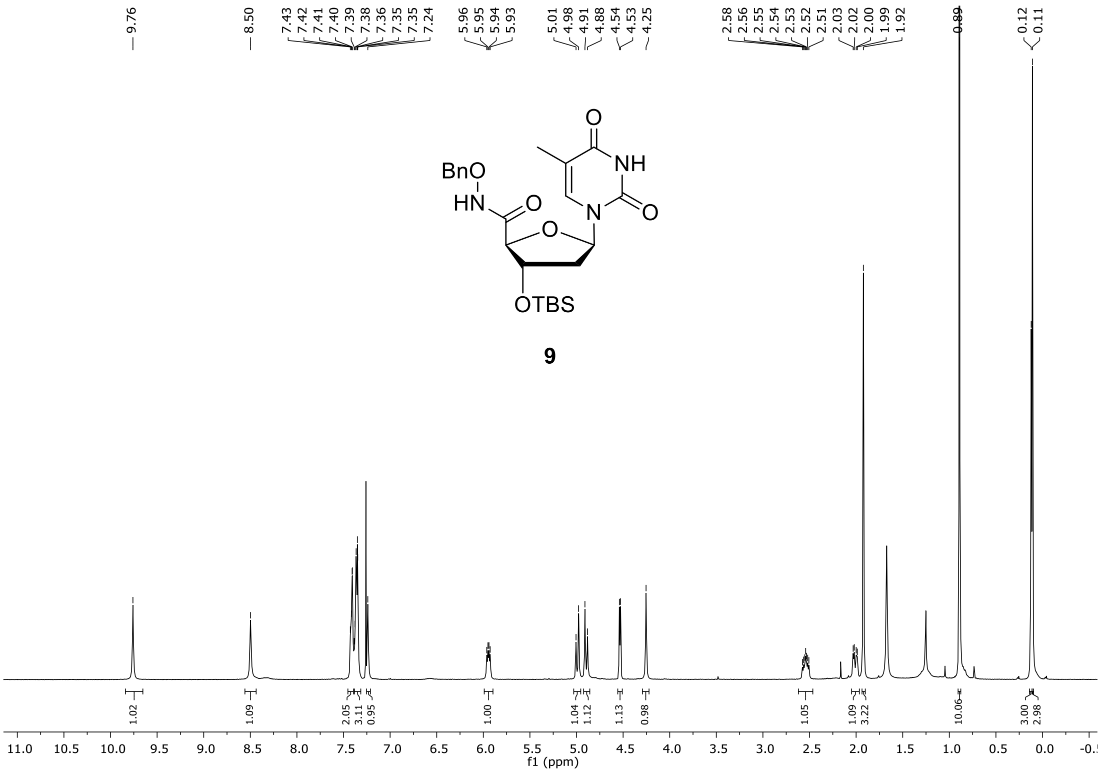


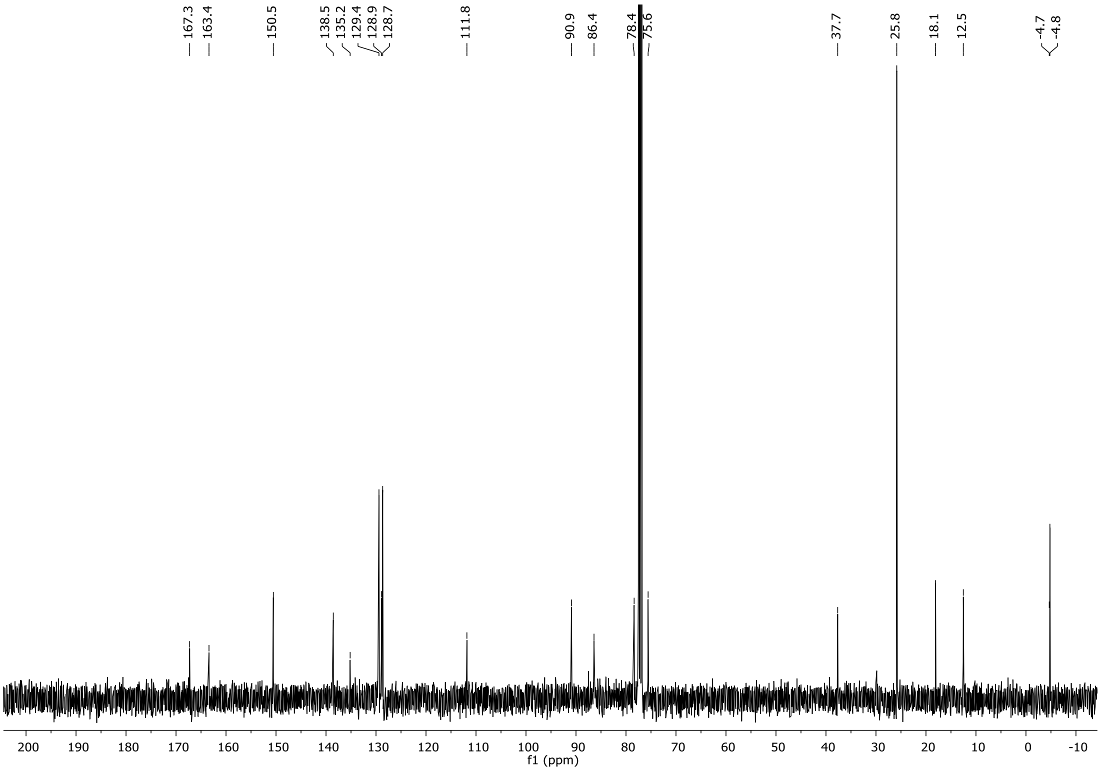

$^{1}H$ (400 MHz, $CD_3OD$) and $^{13}C$ (100 MHz, $CD_3OD$) NMR spectra of **10**

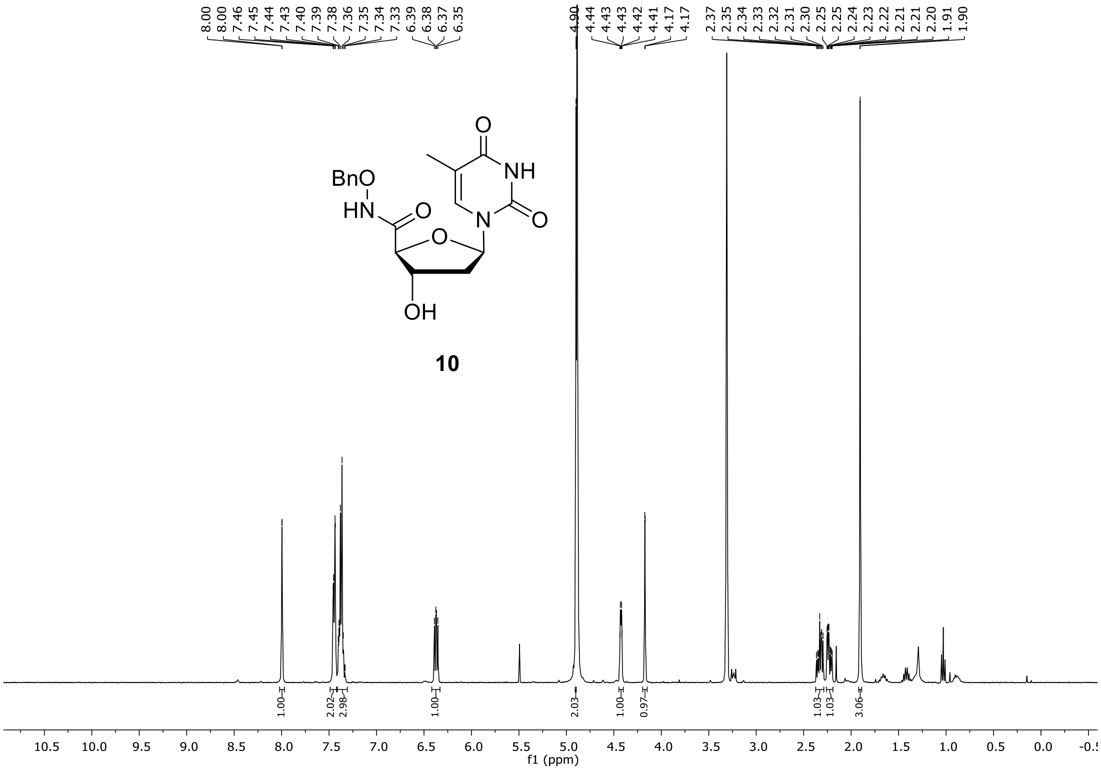


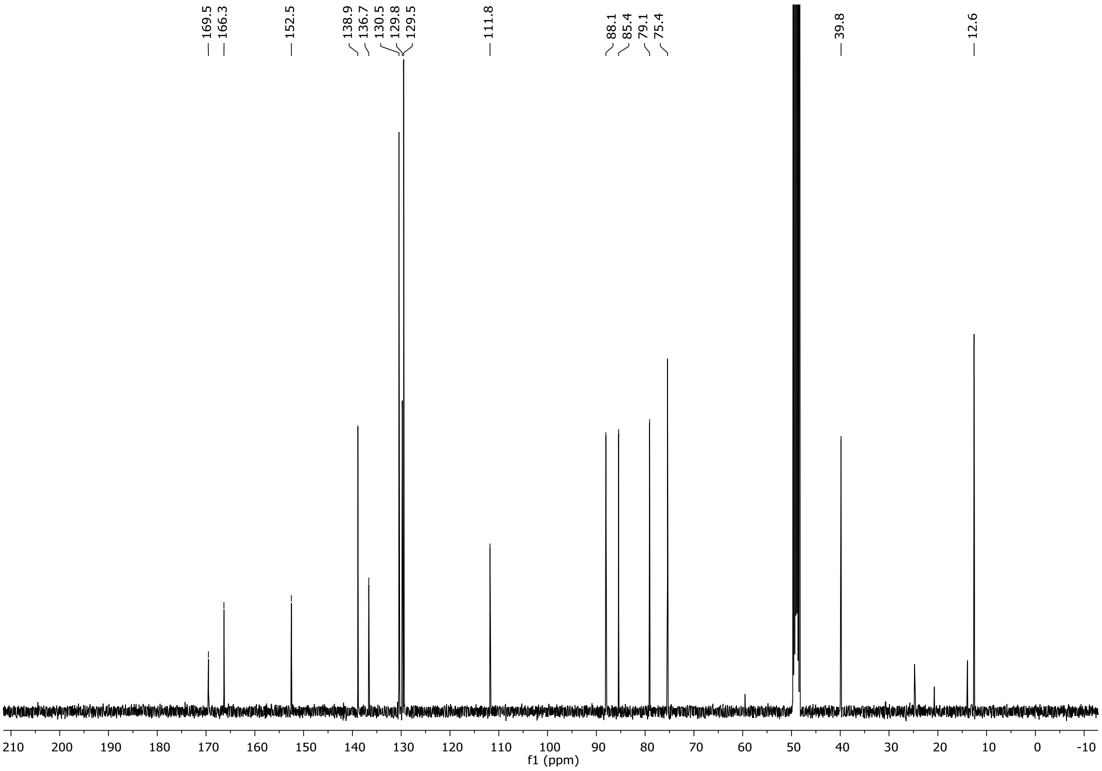

$^{1}H$ (600 MHz, DMSO-$d_6$) and $^{13}C$ (150 MHz, DMSO-$d_6$) NMR spectra of **11**

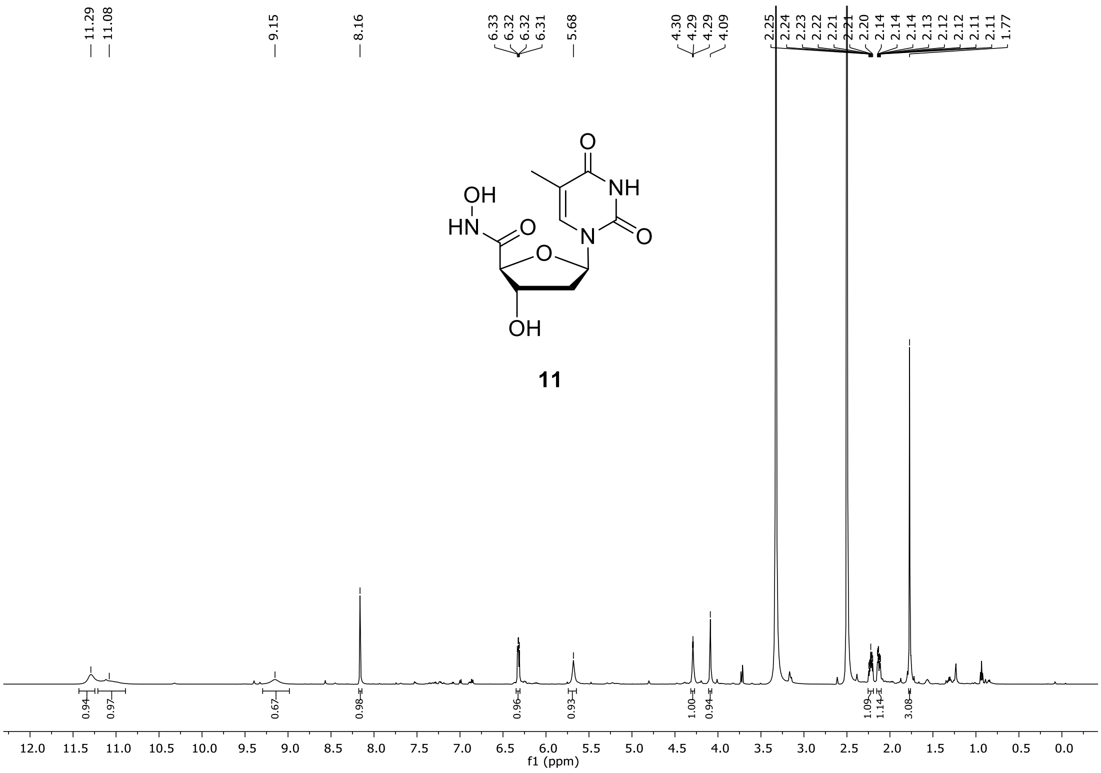


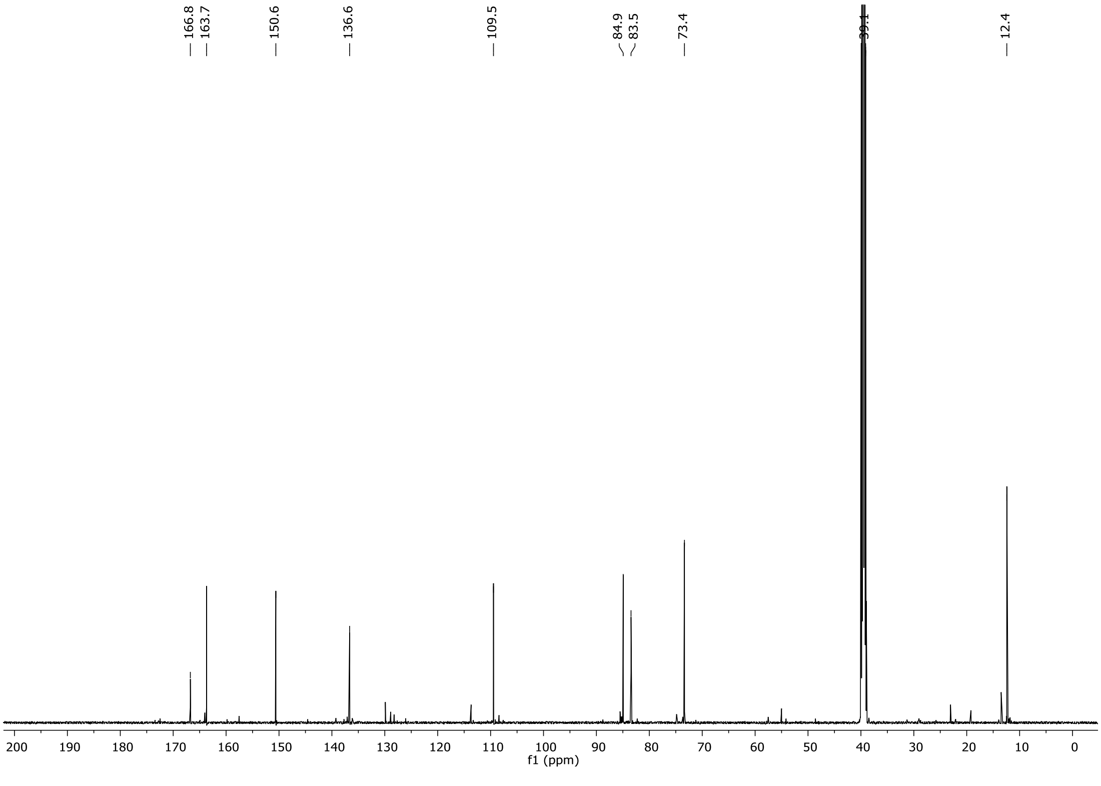

$^{1}H$ (400 MHz, $CDCl_3$) and $^{13}C$ (100 MHz, $CDCl_3$) NMR spectra of **13**

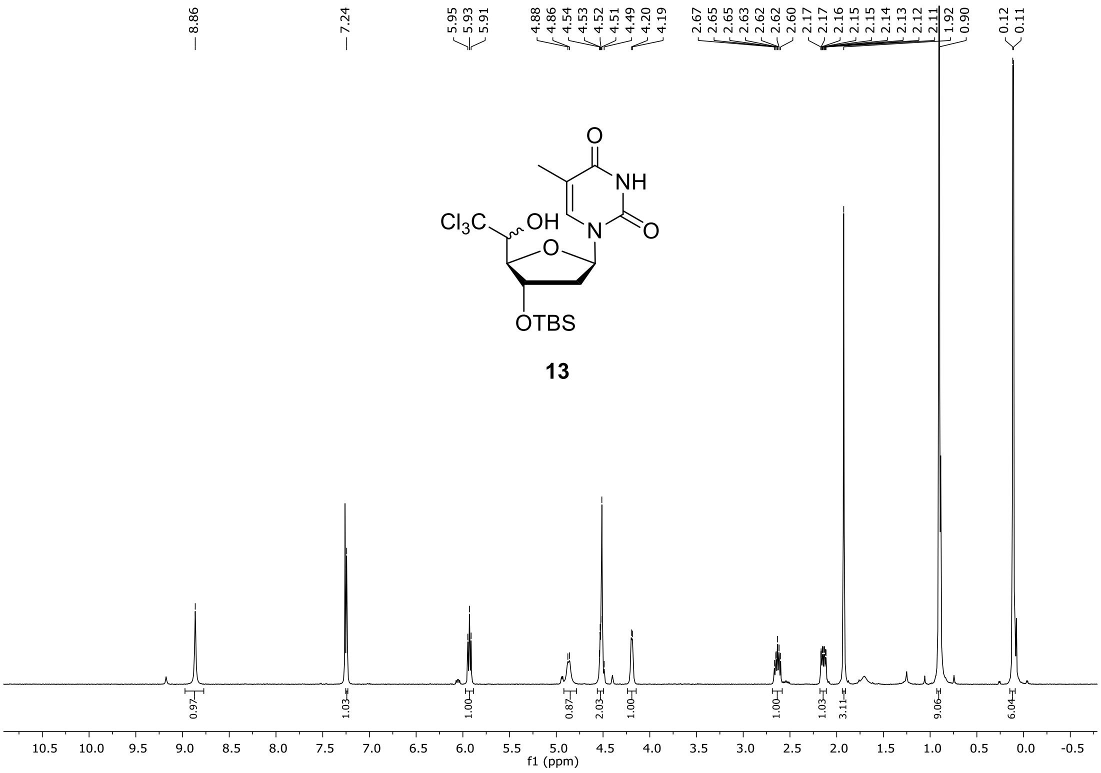


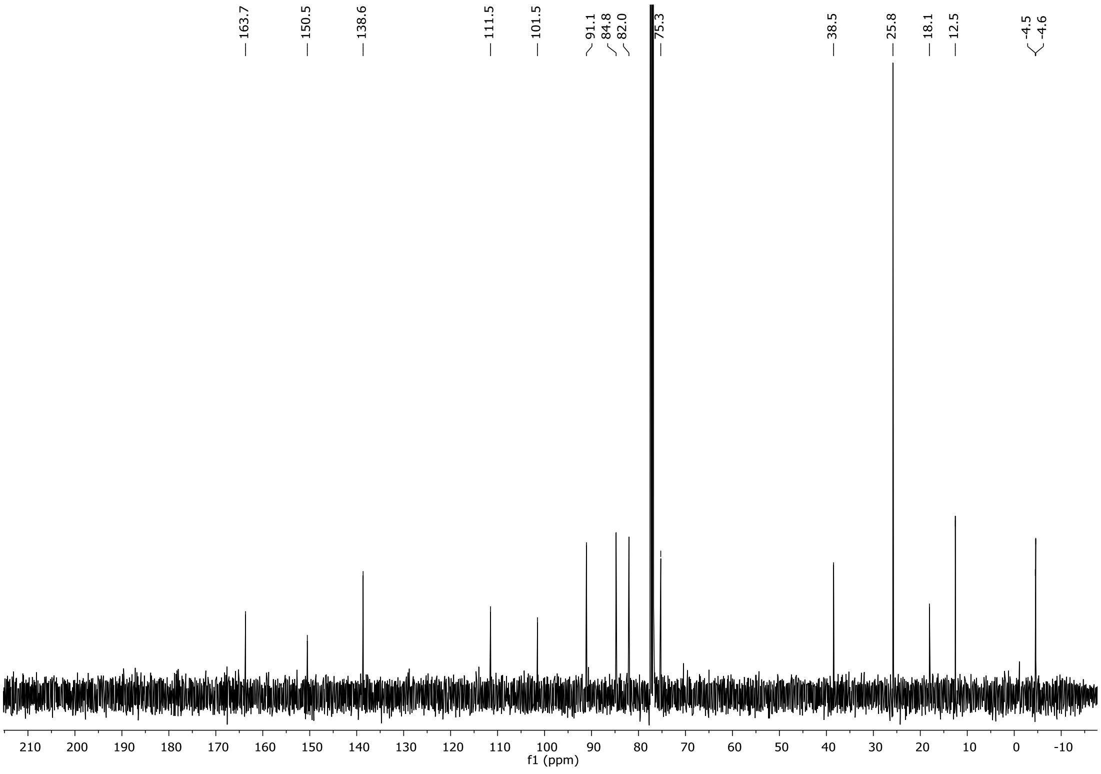

$^{1}H$ (400 MHz, $CDCl_3$) NMR spectrum of **16**

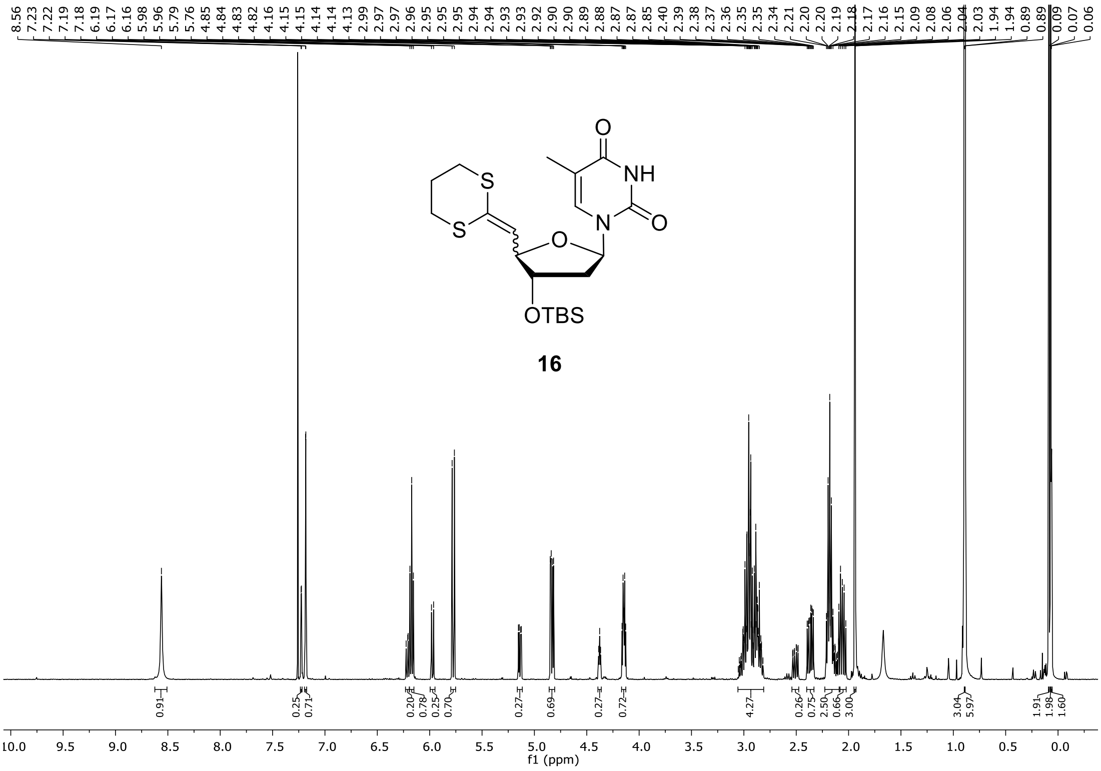

$^{1}H$ (400 MHz, $CDCl_3$) and $^{13}C$ (100 MHz, $CDCl_3$) NMR spectra of **17**

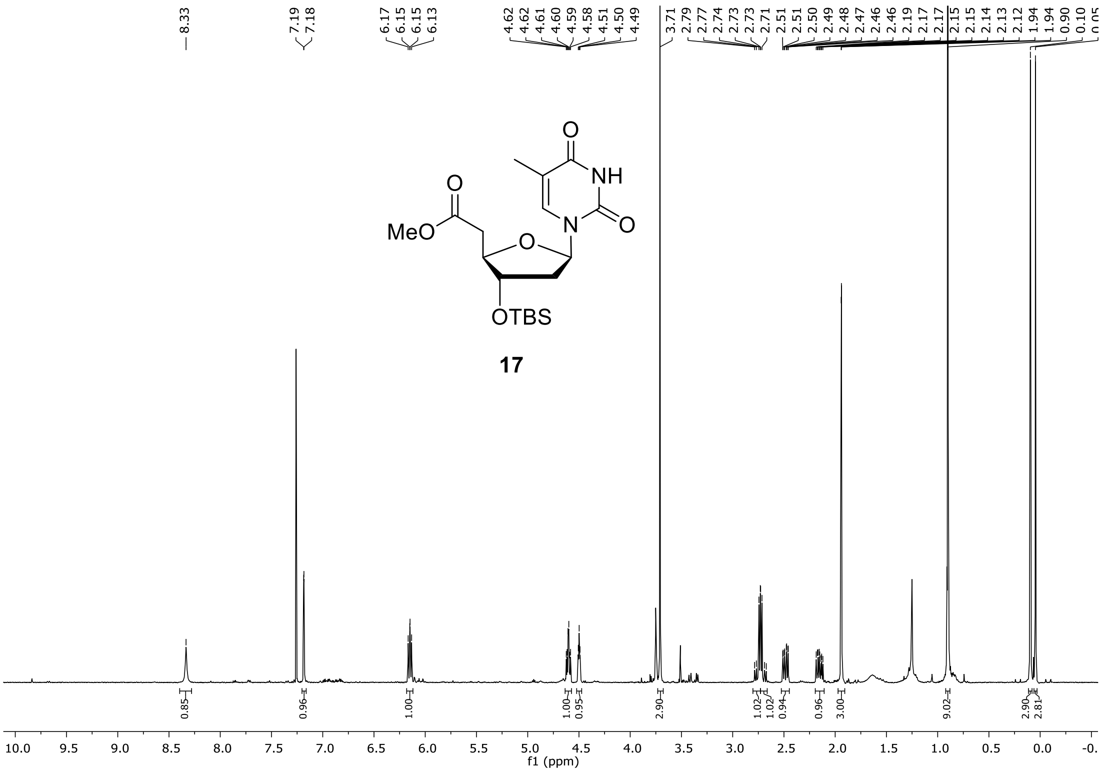


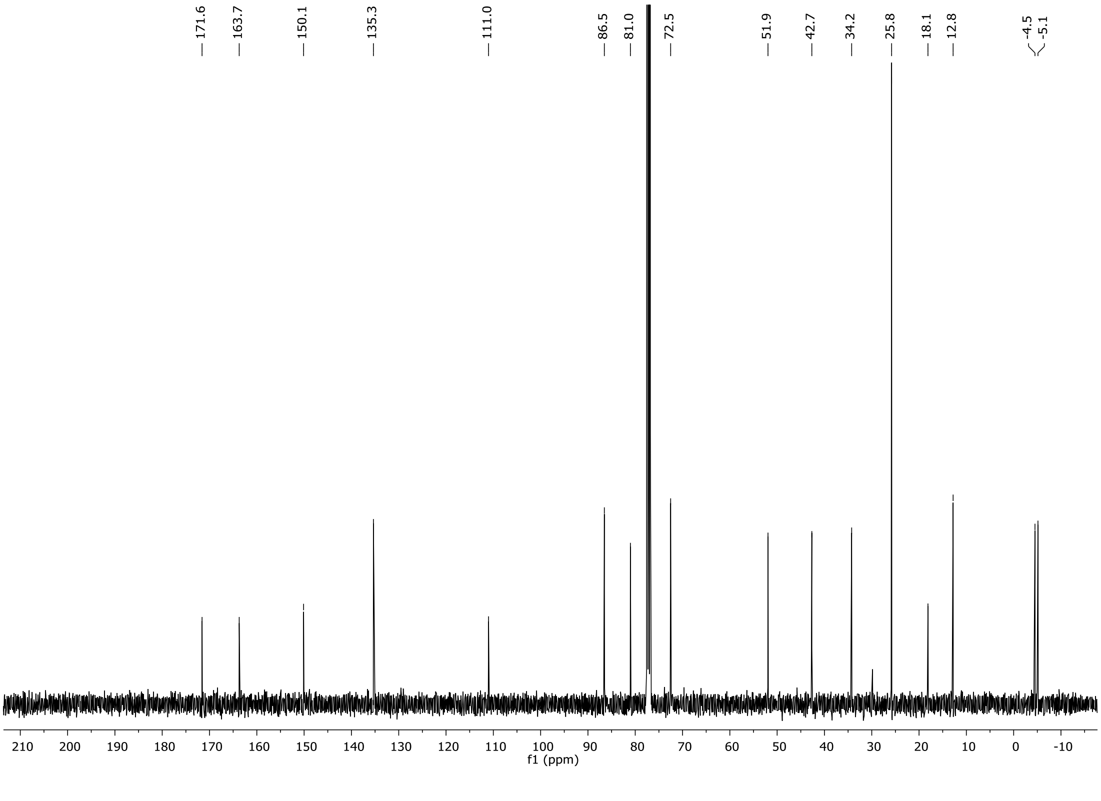

$^{1}$H (400 MHz, $CDCl_3$) and $^{13}$C (100 MHz, $CDCl_3$) NMR spectra of **19**

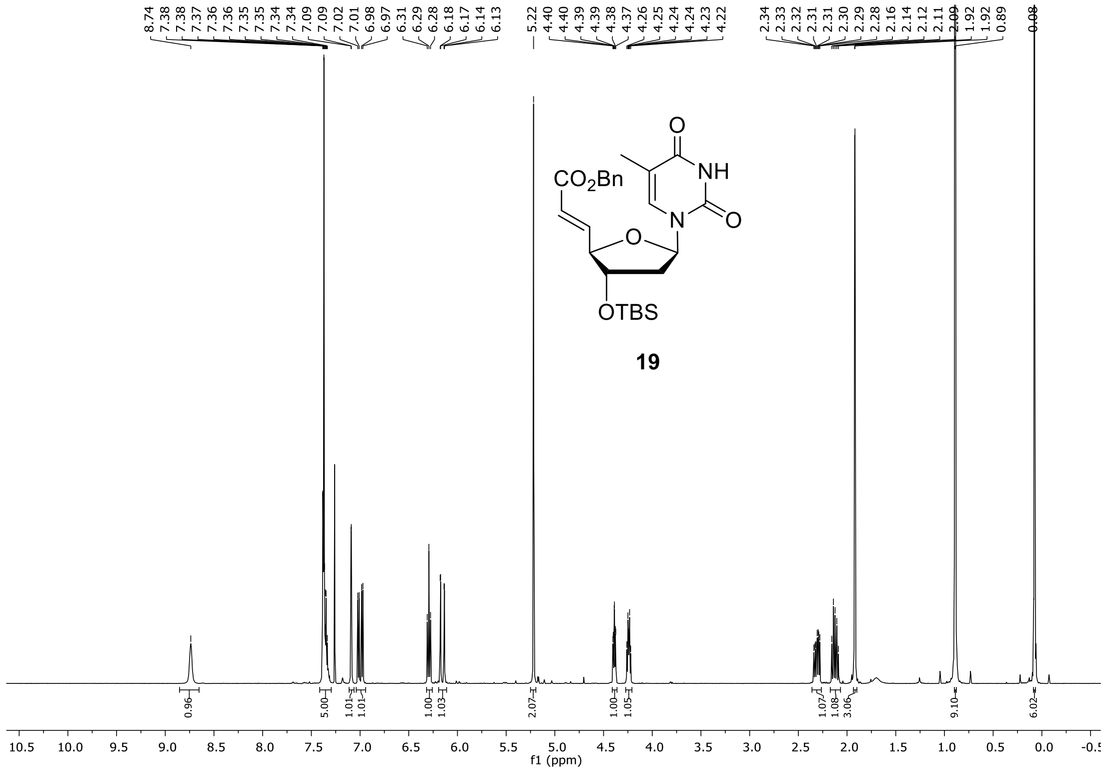


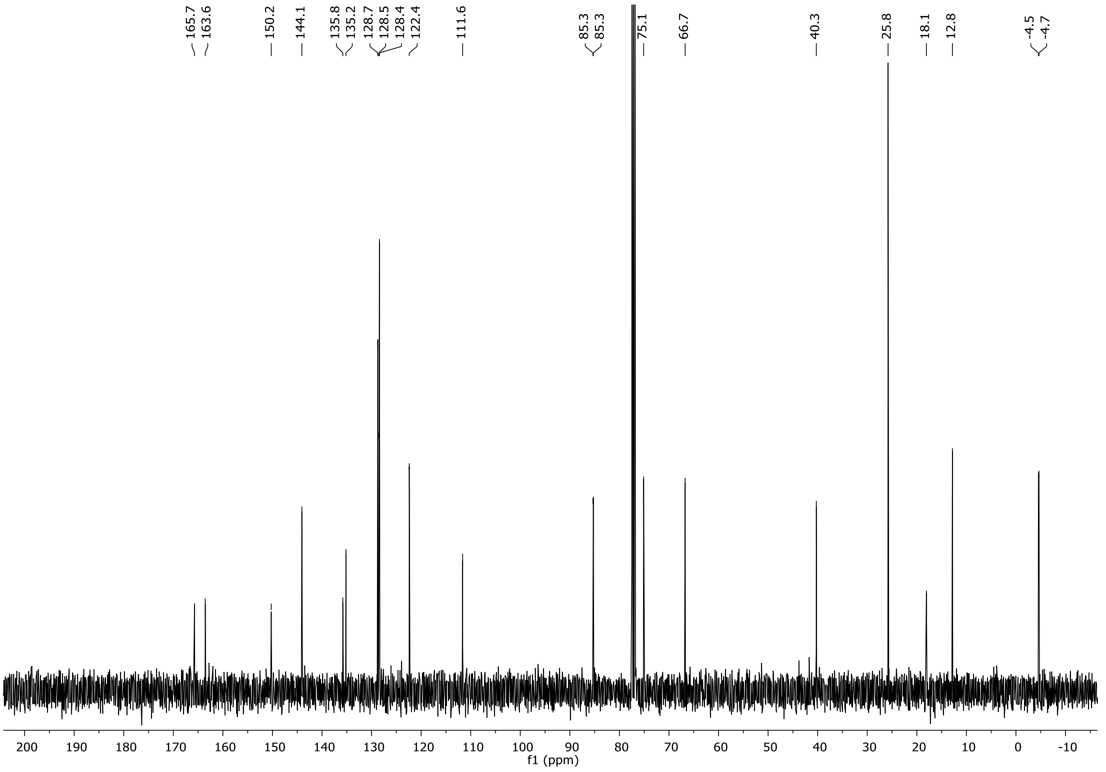

$^{1}H$ (400 MHz, DMSO-$d_6$) and $^{13}C$ (100 MHz, DMSO-$d_6$) NMR spectra of **20**

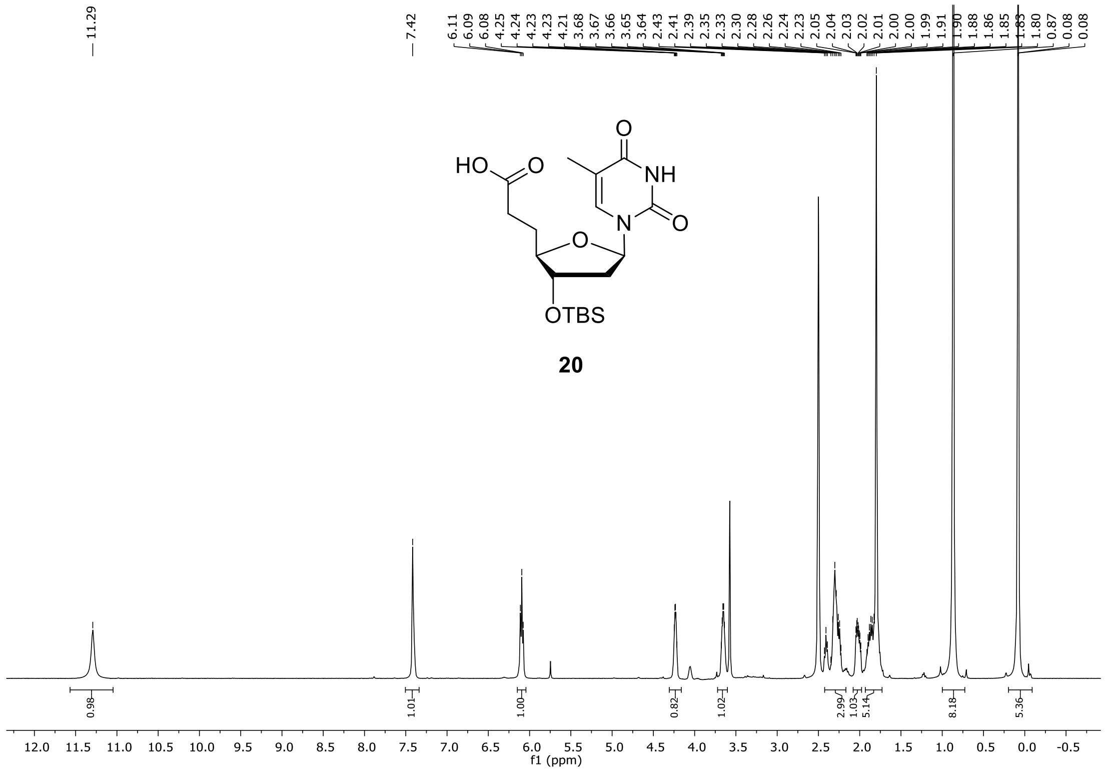


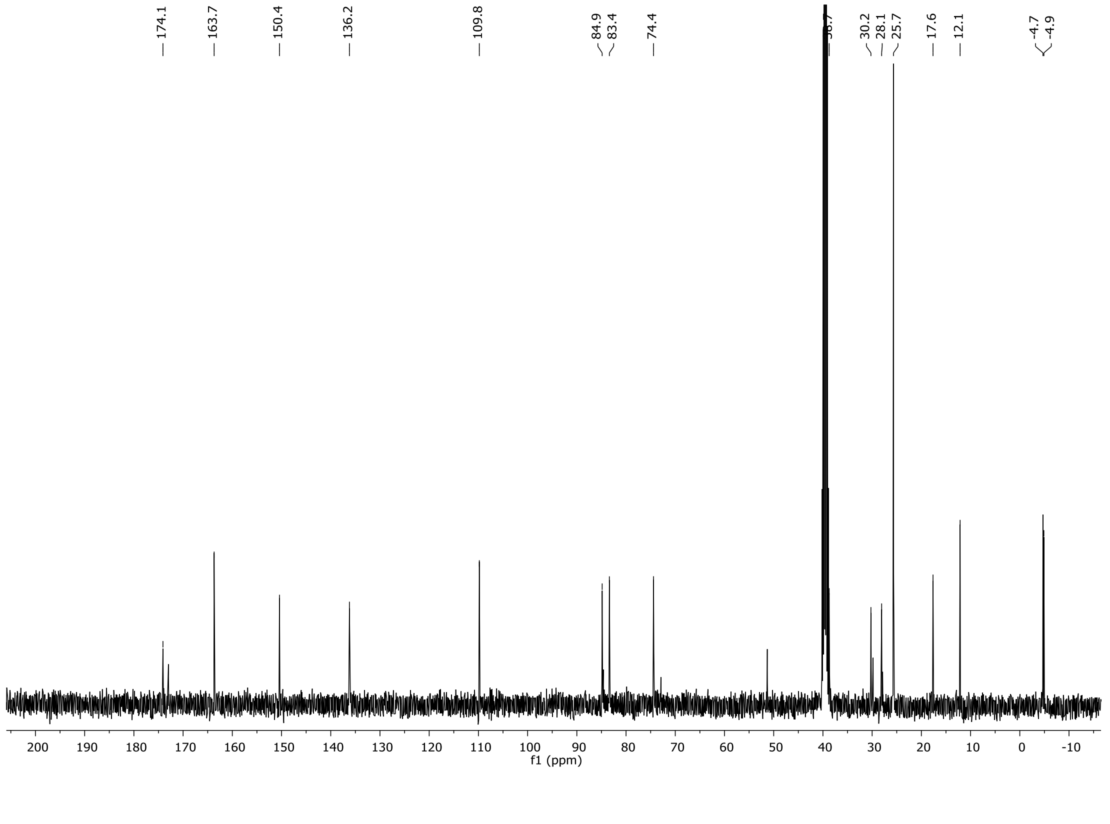

$^{1}$H (600 MHz, DMSO-$d_6$) and $^{13}$C (150 MHz, DMSO-$d_6$) NMR spectra of **21**

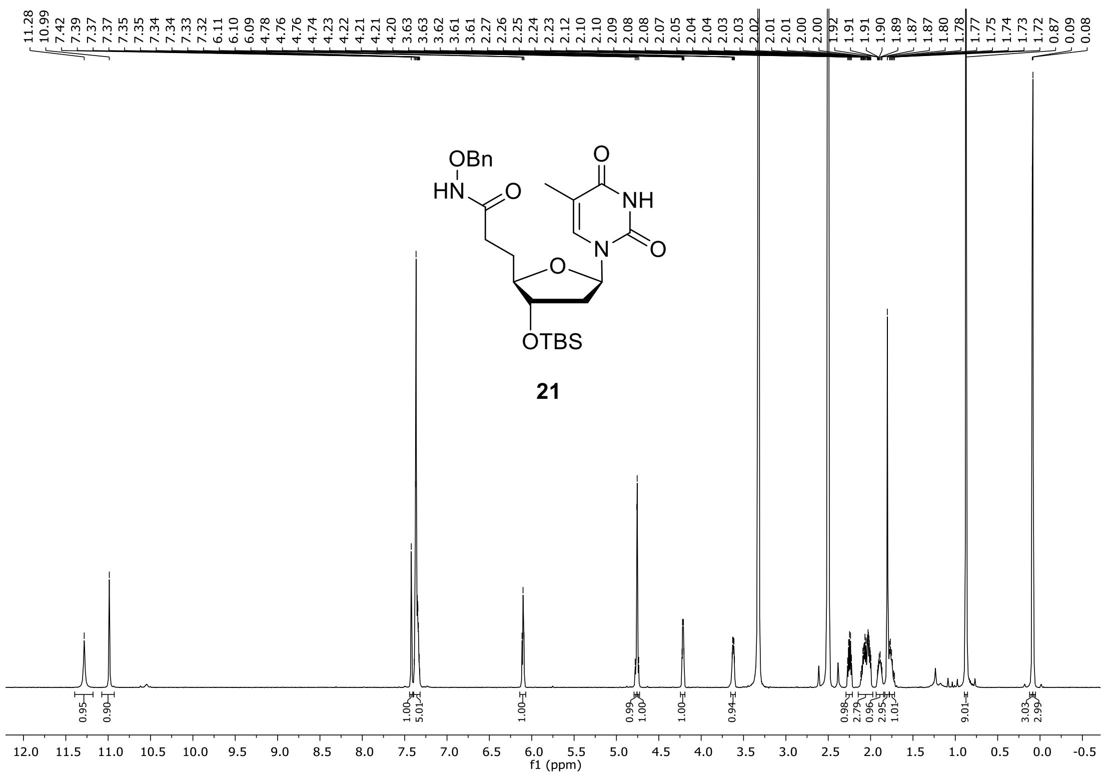


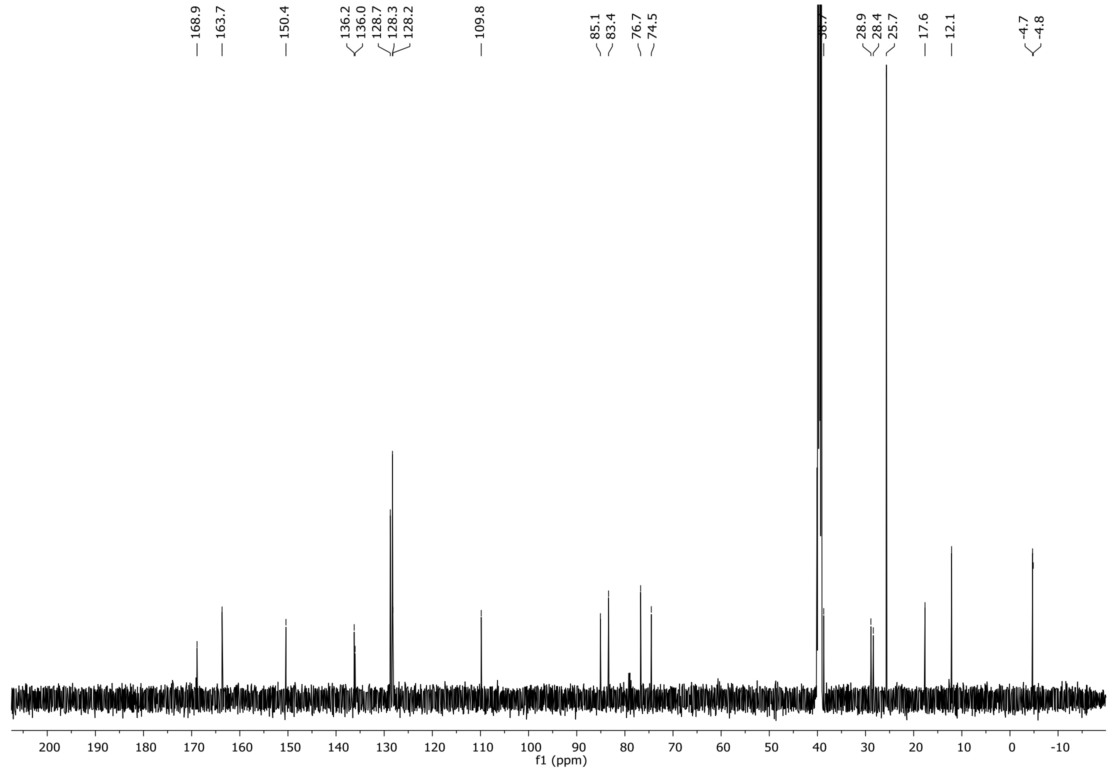

$^{1}$H (600 MHz, DMSO-$d_6$) and $^{13}$C (150 MHz, DMSO-$d_6$) NMR spectra of **22**

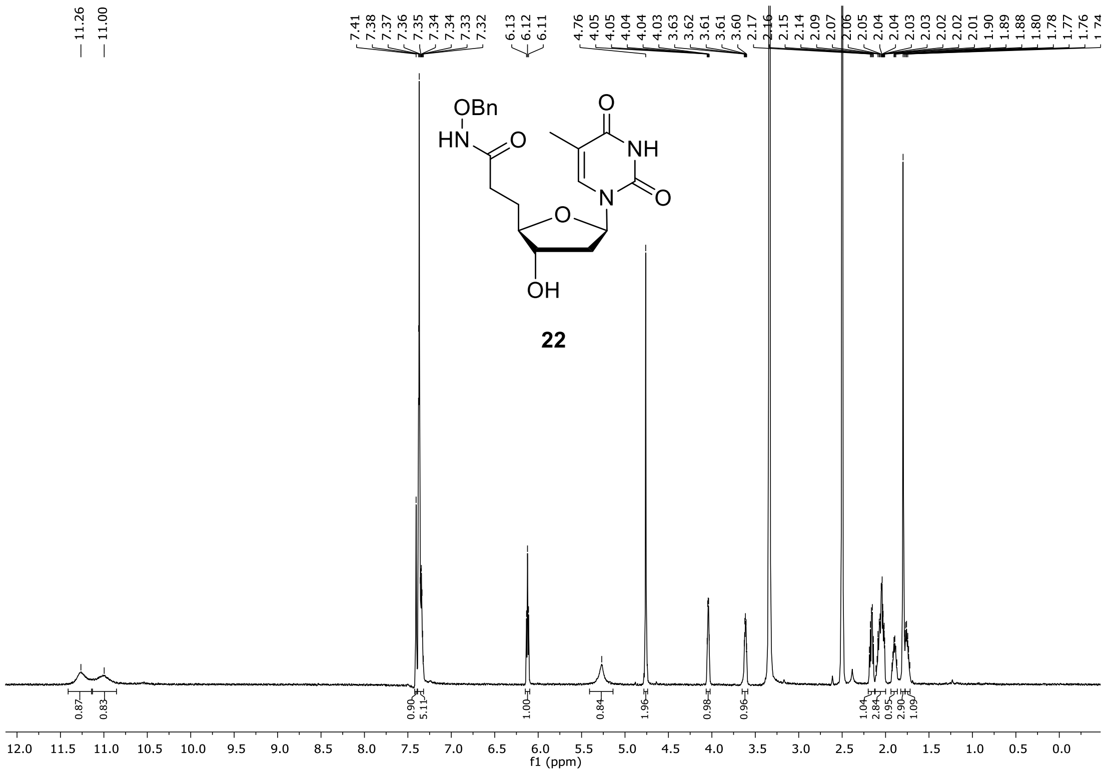

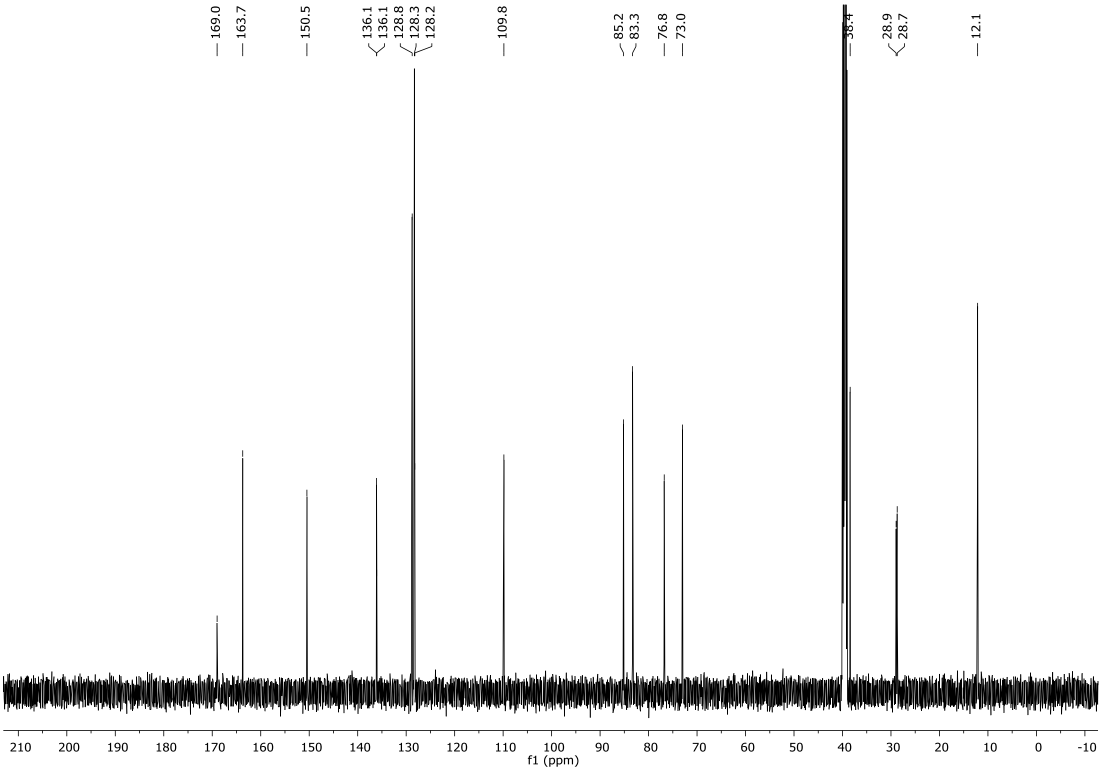

$^{1}$H (600 MHz, DMSO-$d_6$) and $^{13}$C (150 MHz, DMSO-$d_6$) NMR spectra of **23**

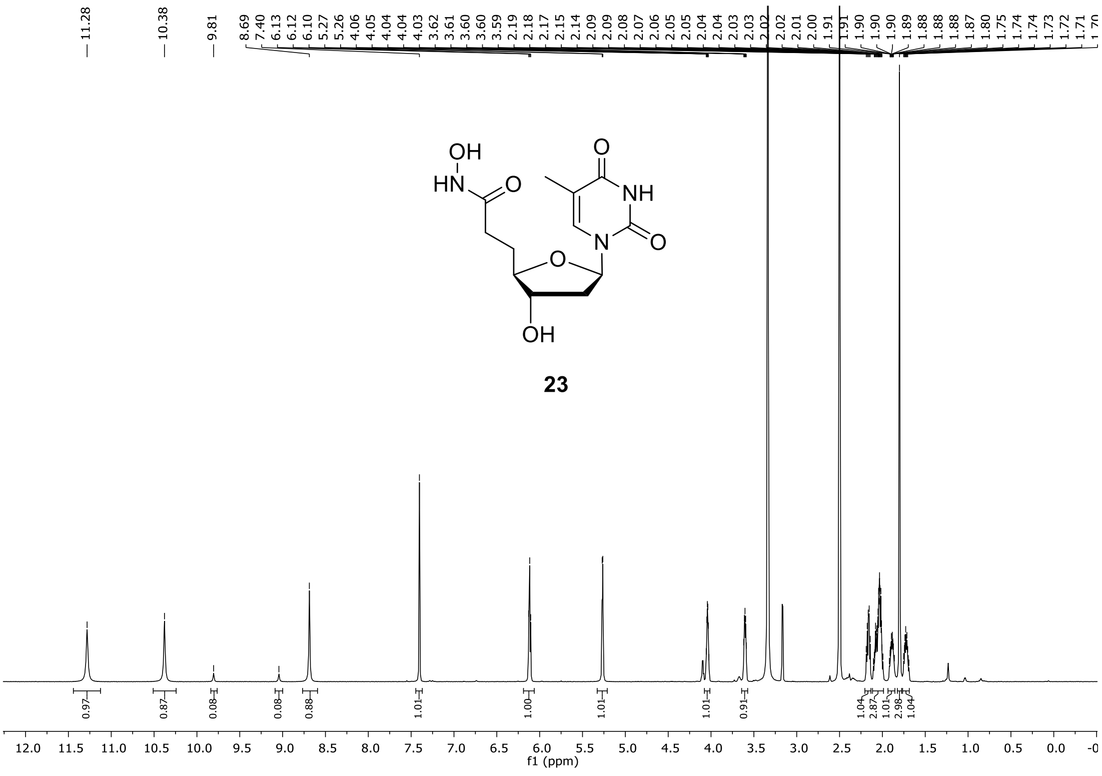


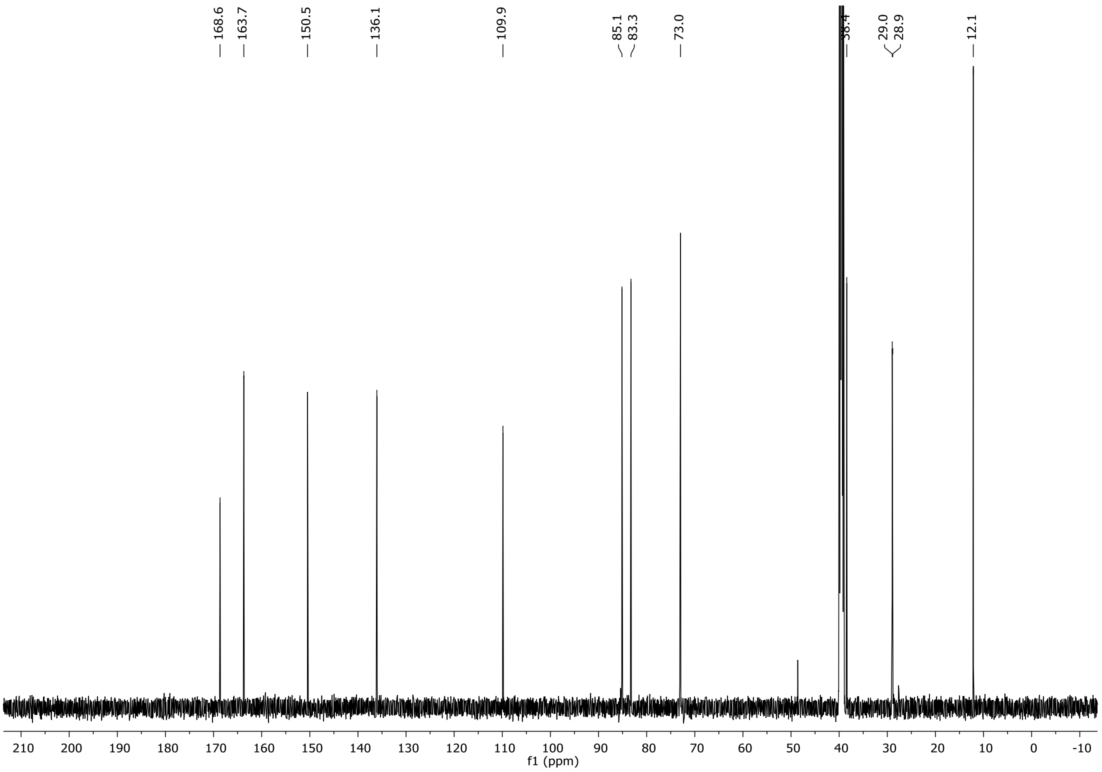

$^{1}$H (600 MHz, DMSO-$d_6$) and $^{13}$C (150 MHz, DMSO-$d_6$) NMR spectra of **24**

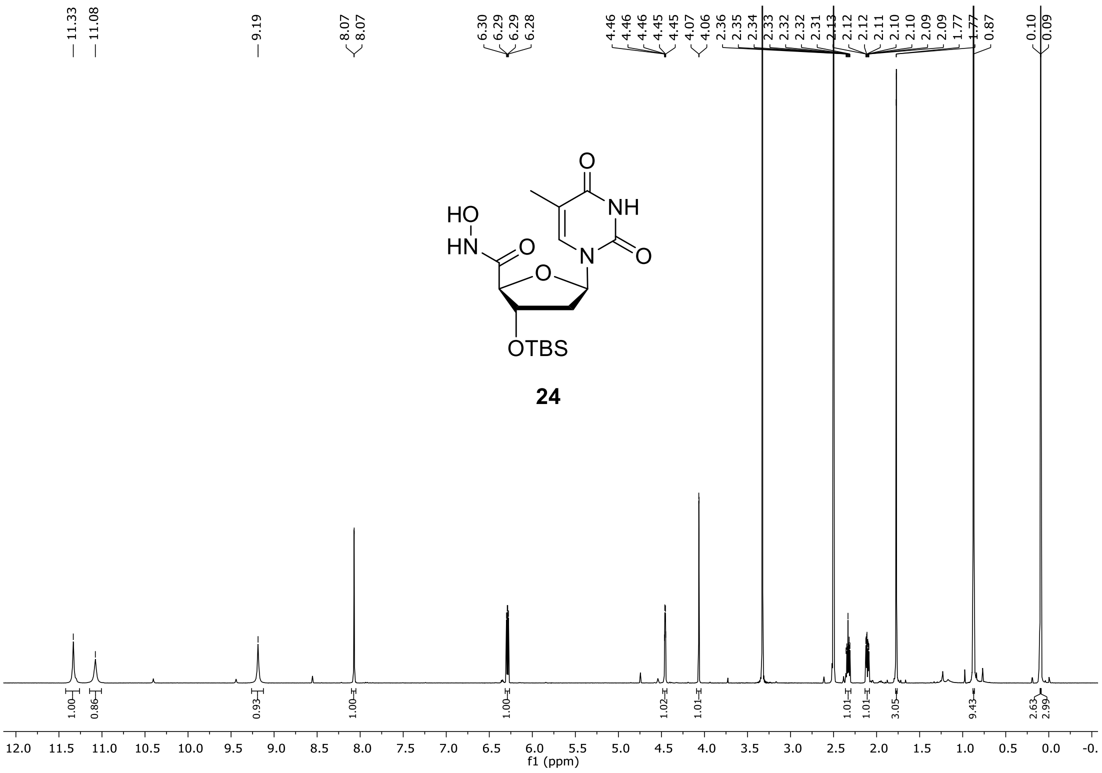


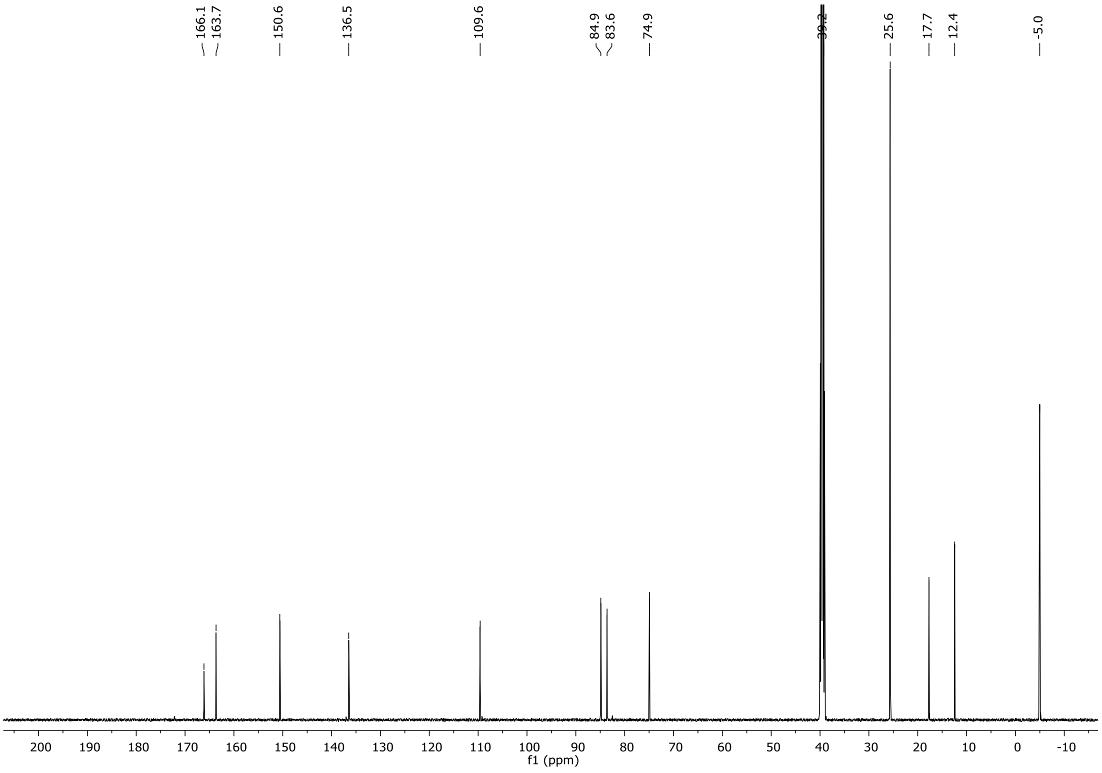

$^{1}H$ (600 MHz, $CDCl_3$) and $^{13}C$ (150 MHz, $CDCl_3$) NMR spectra of **25**

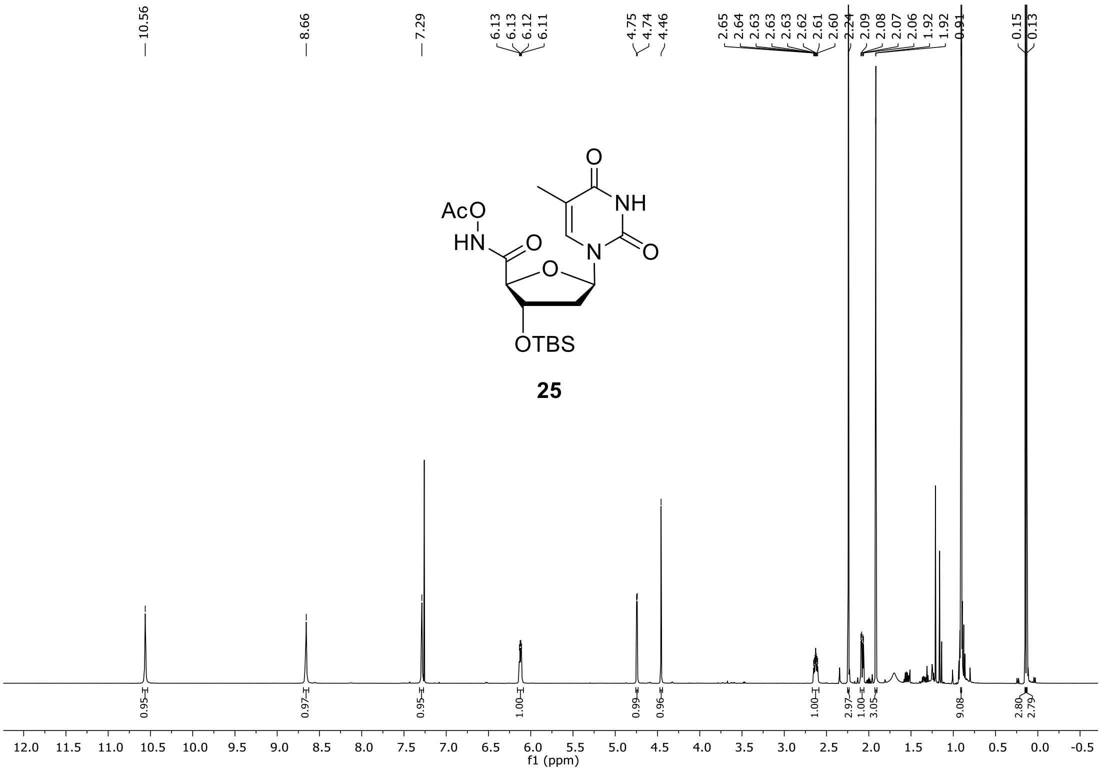


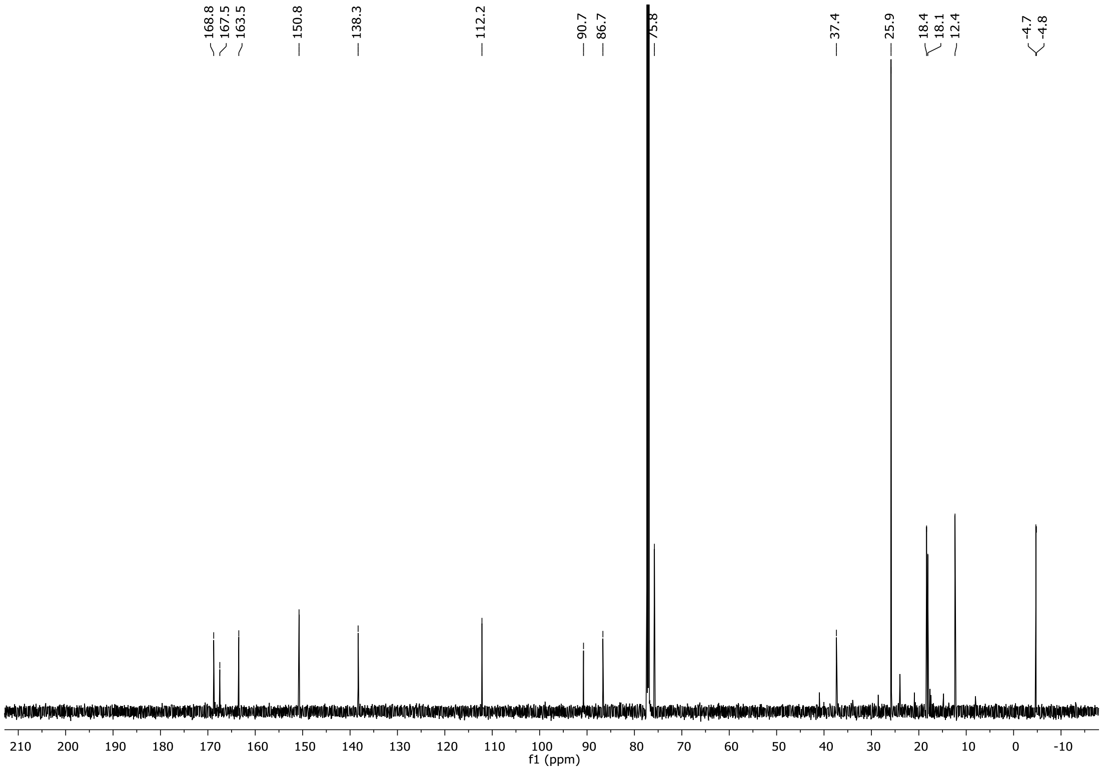

$^{1}$H (400 MHz, DMSO-$d_6$) and $^{13}$C (100 MHz, DMSO-$d_6$) NMR spectra of **26**

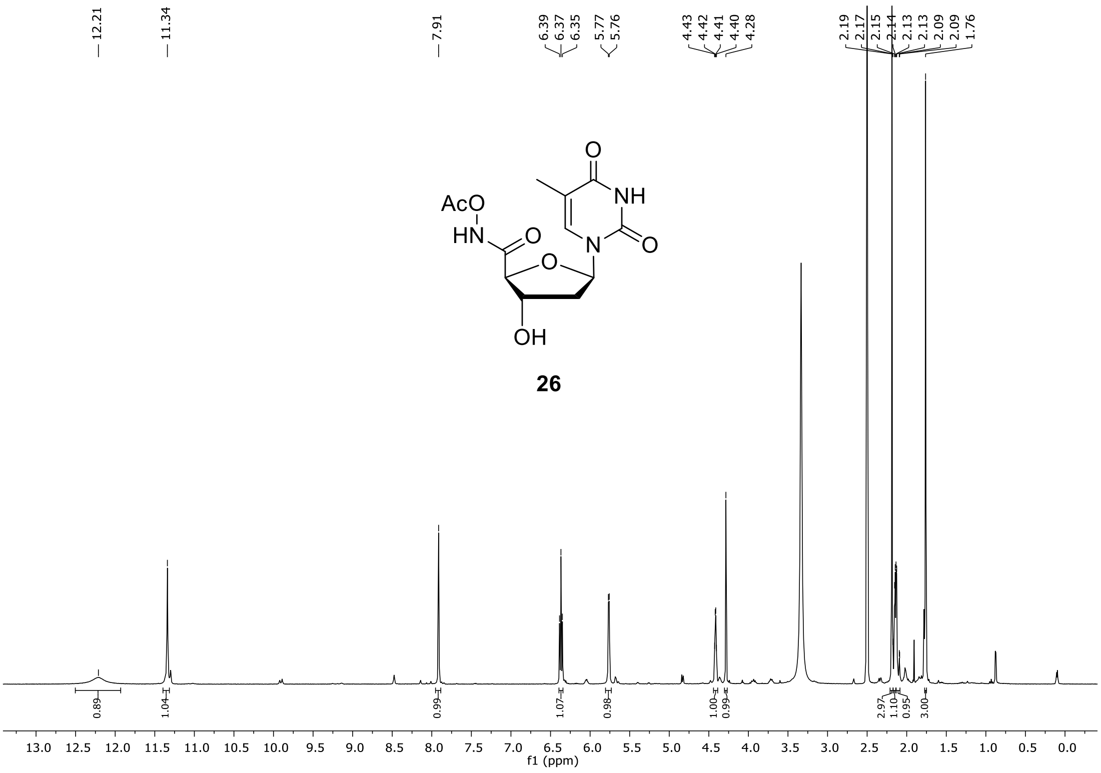


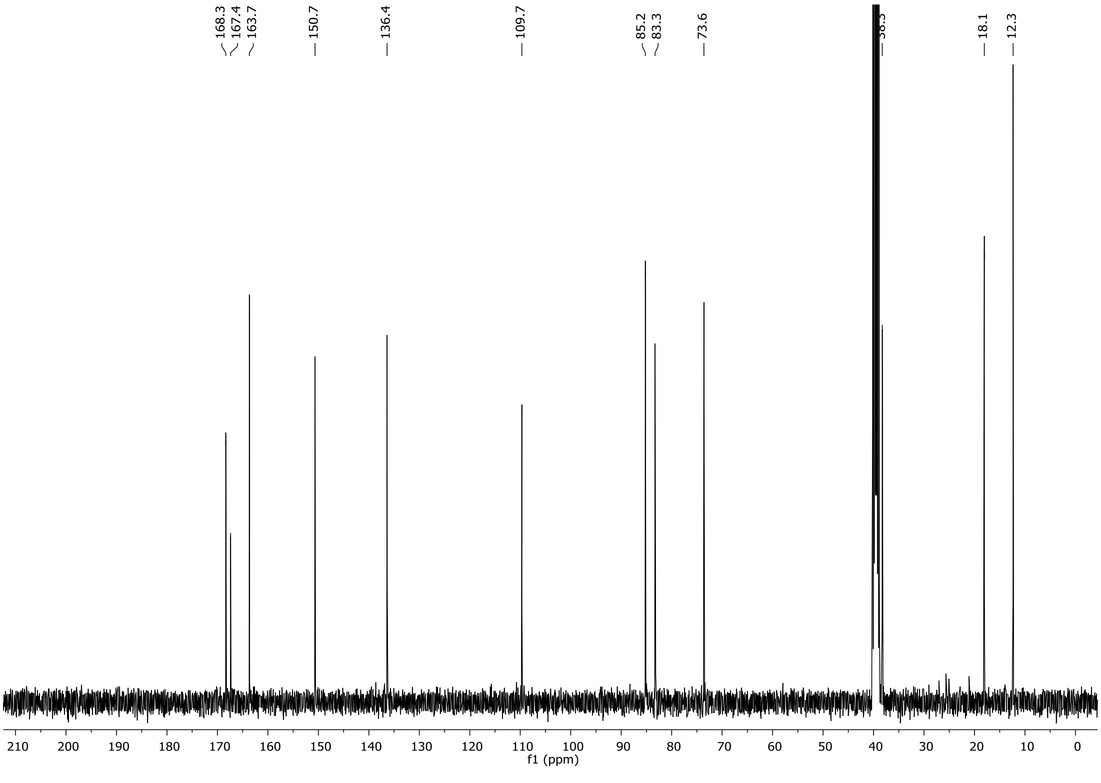

$^{1}H$ (400 MHz, $CDCl_3$) and $^{13}C$ (100 MHz, $CDCl_3$) NMR spectra of **27**

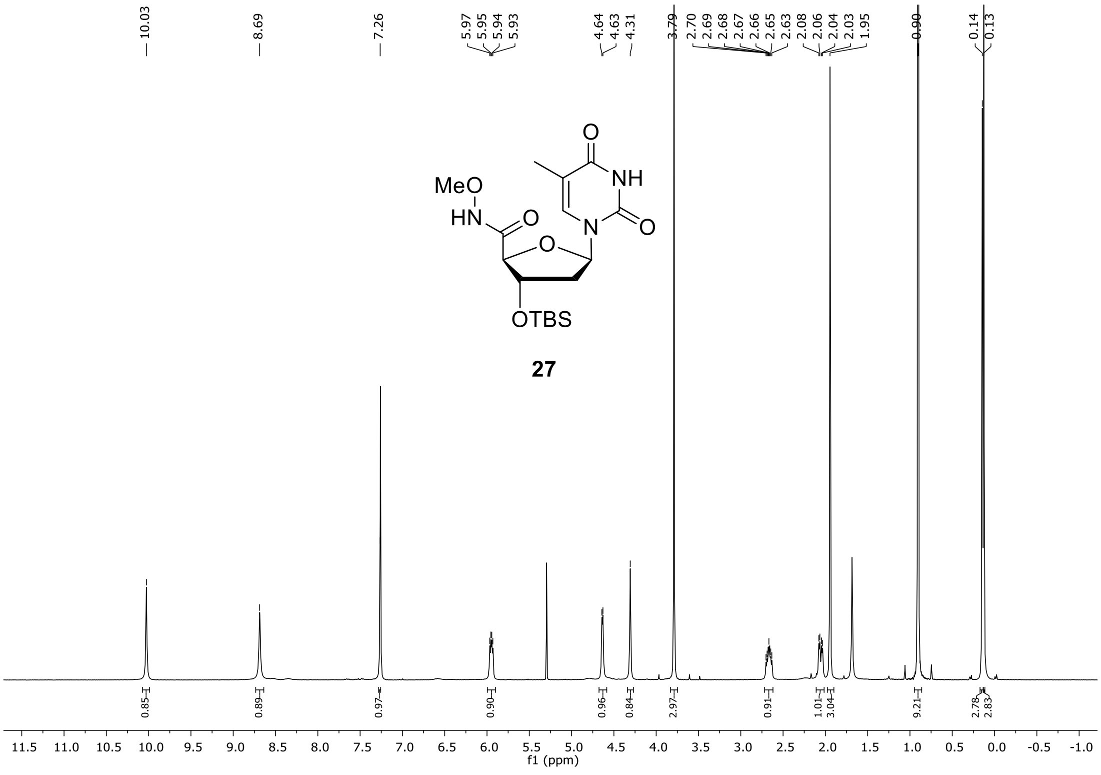


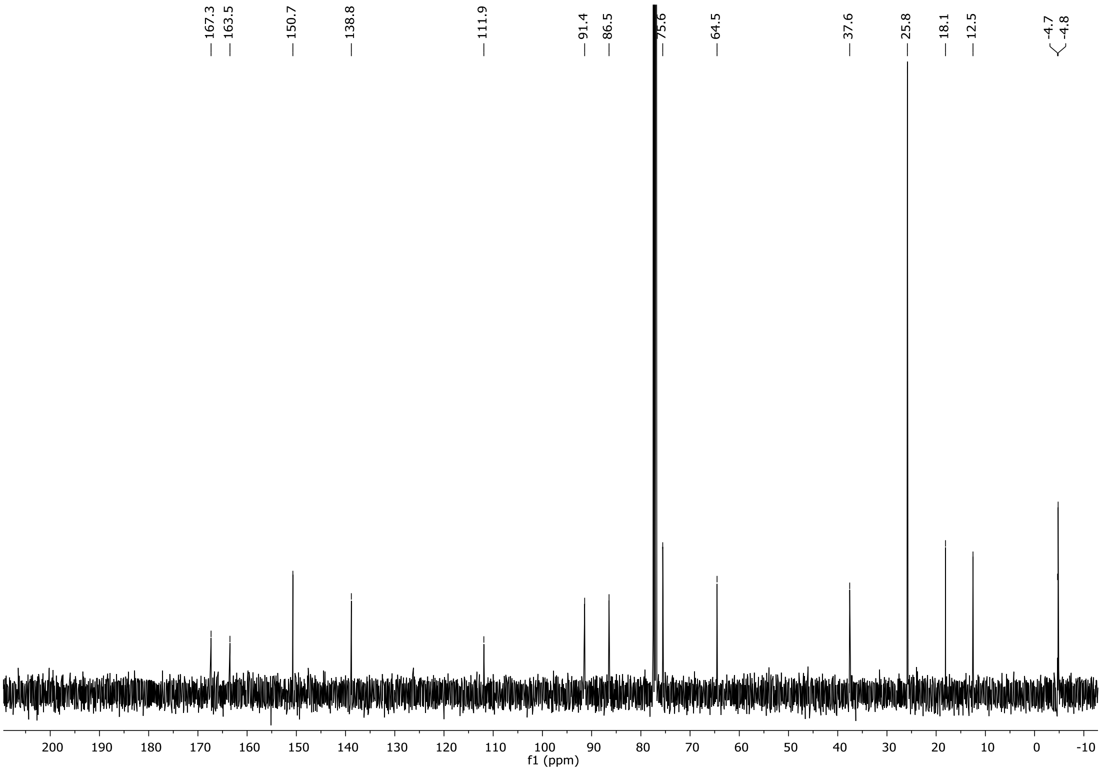

$^{1}H$ (400 MHz, DMSO-$d_6$) and $^{13}C$ (100 MHz, DMSO-$d_6$) NMR spectra of **28**

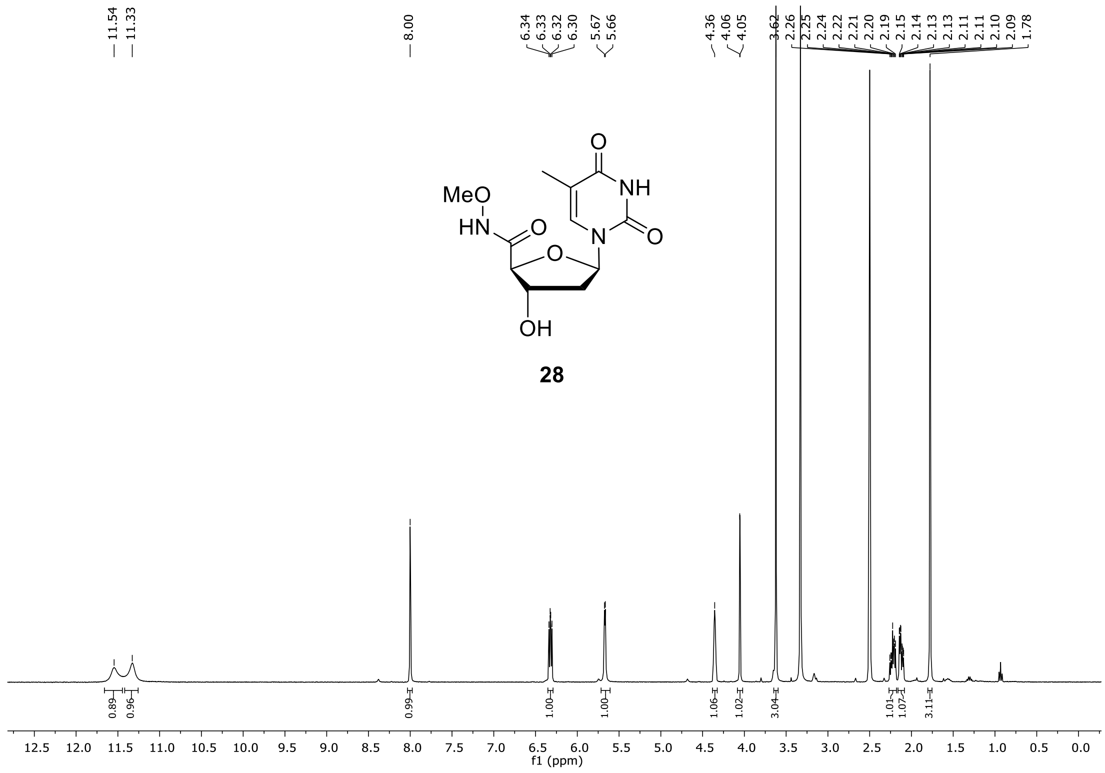


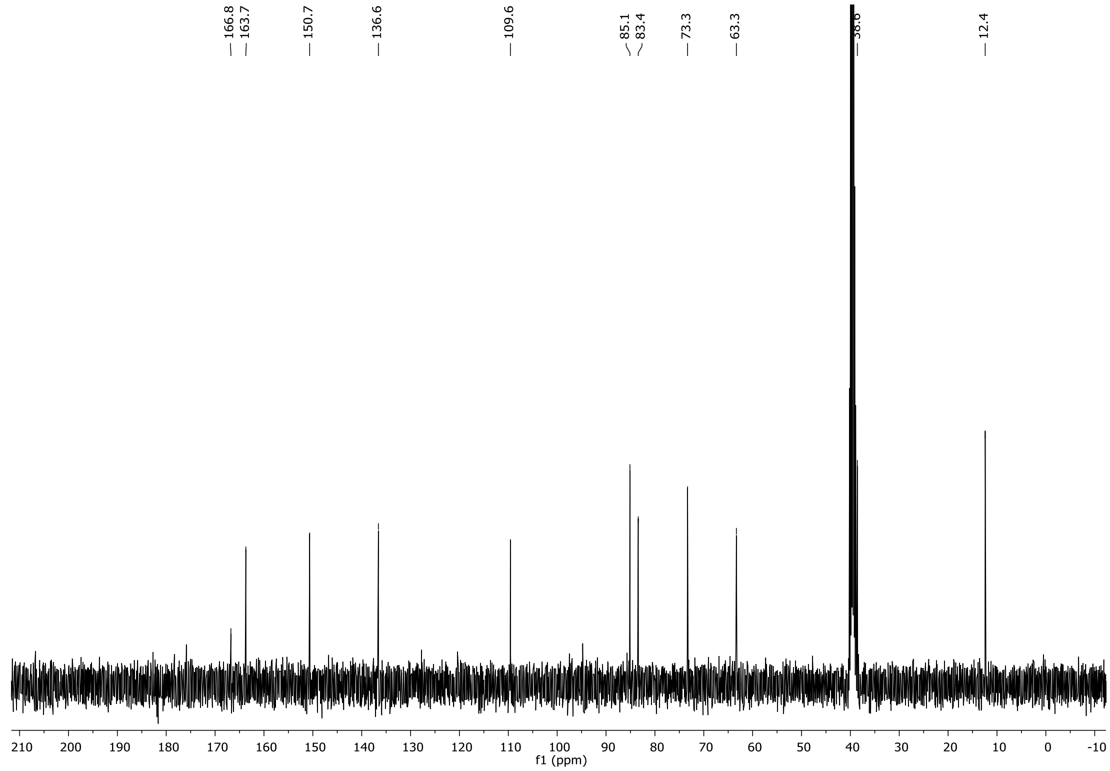

$^{1}H$ (600 MHz, $CDCl_3$) and $^{13}C$ (150 MHz, $CDCl_3$) NMR spectra of **29**

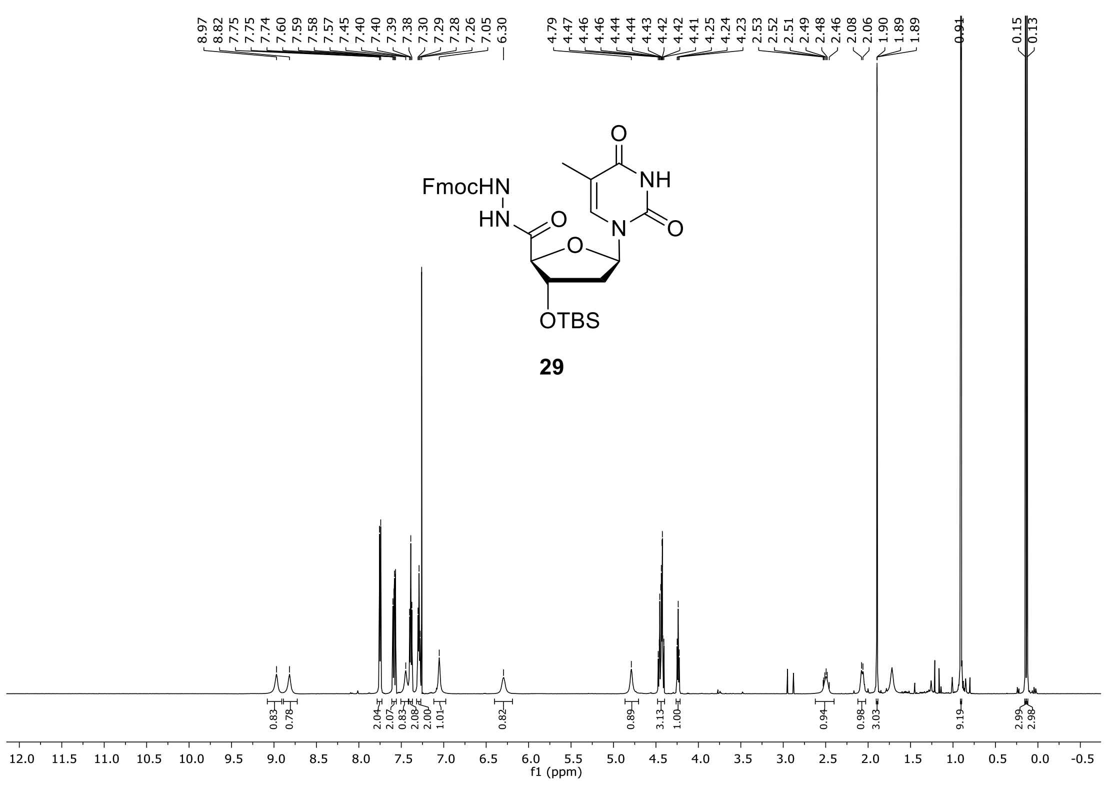


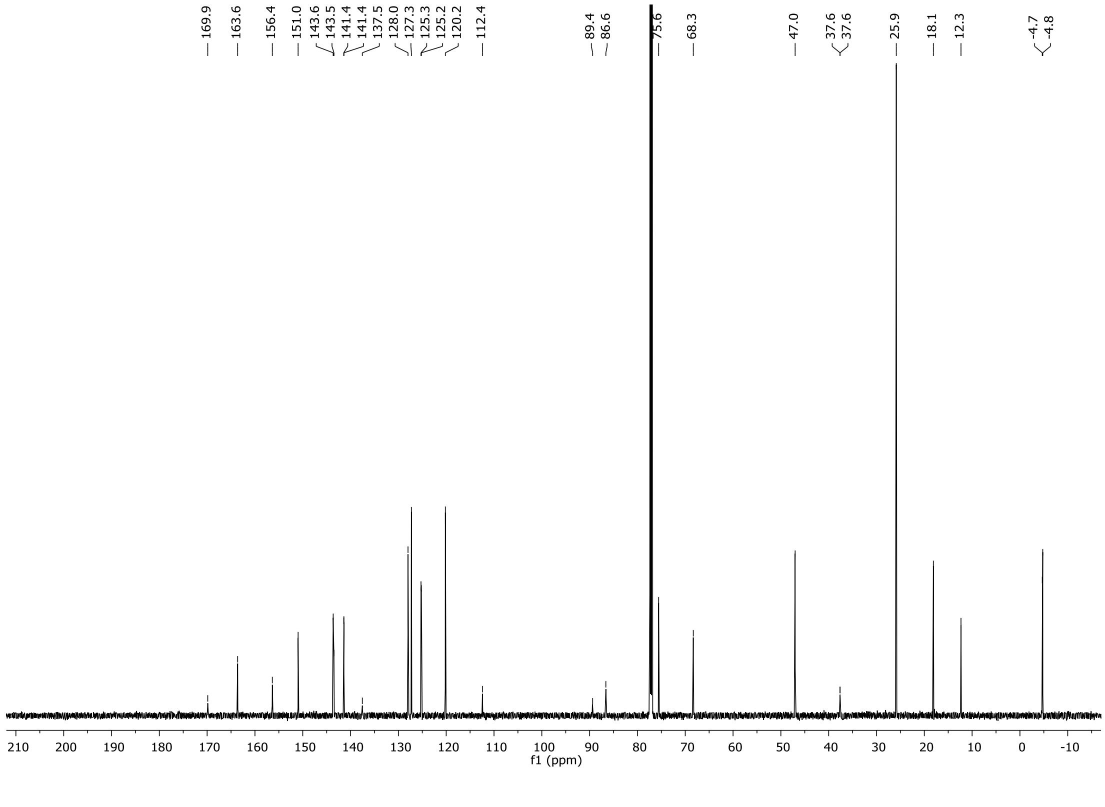

$^{1}$H (600 MHz, DMSO-$d_6$) and $^{13}$C (150 MHz, DMSO-$d_6$) NMR spectra of **30**

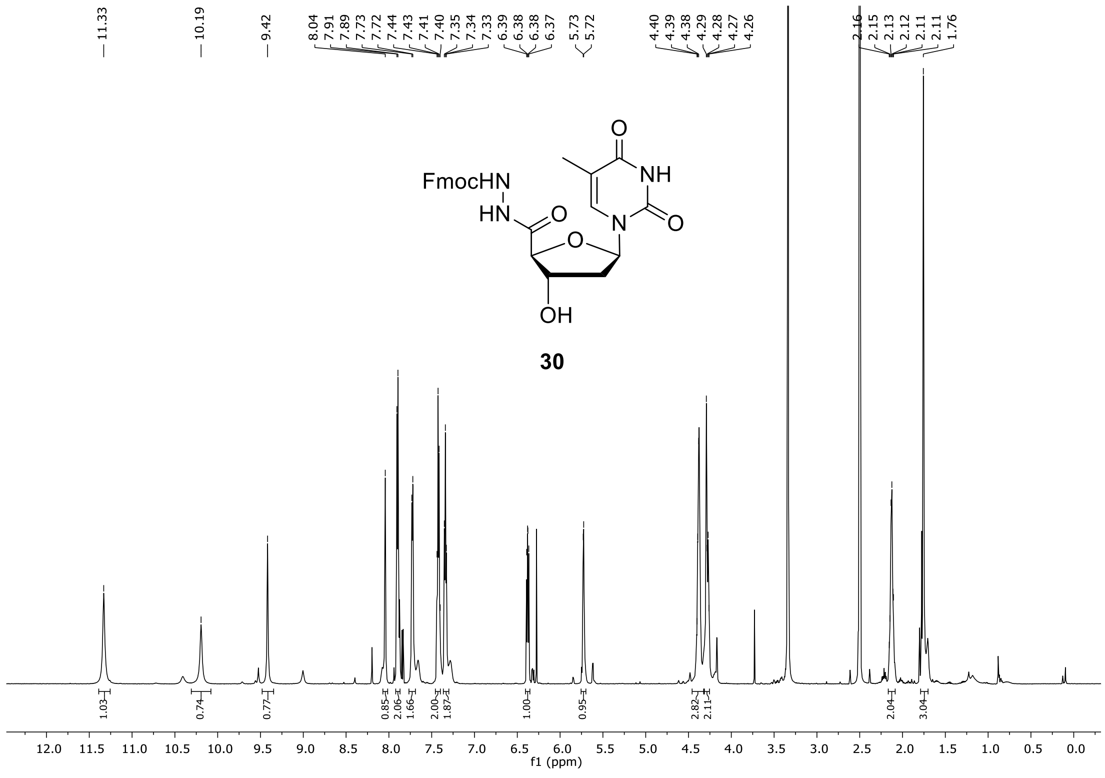

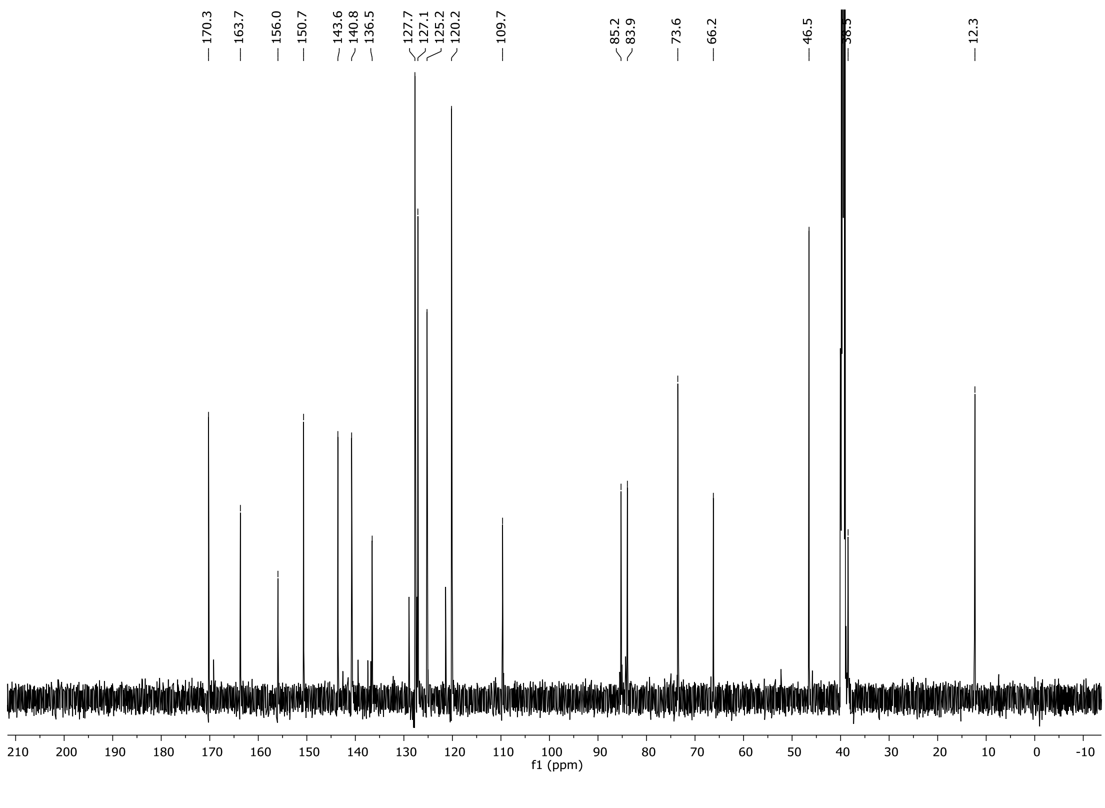

$^{1}$H (400 MHz, DMSO-$d_6$) and $^{13}$C (100 MHz, DMSO-$d_6$) NMR spectra of **31**

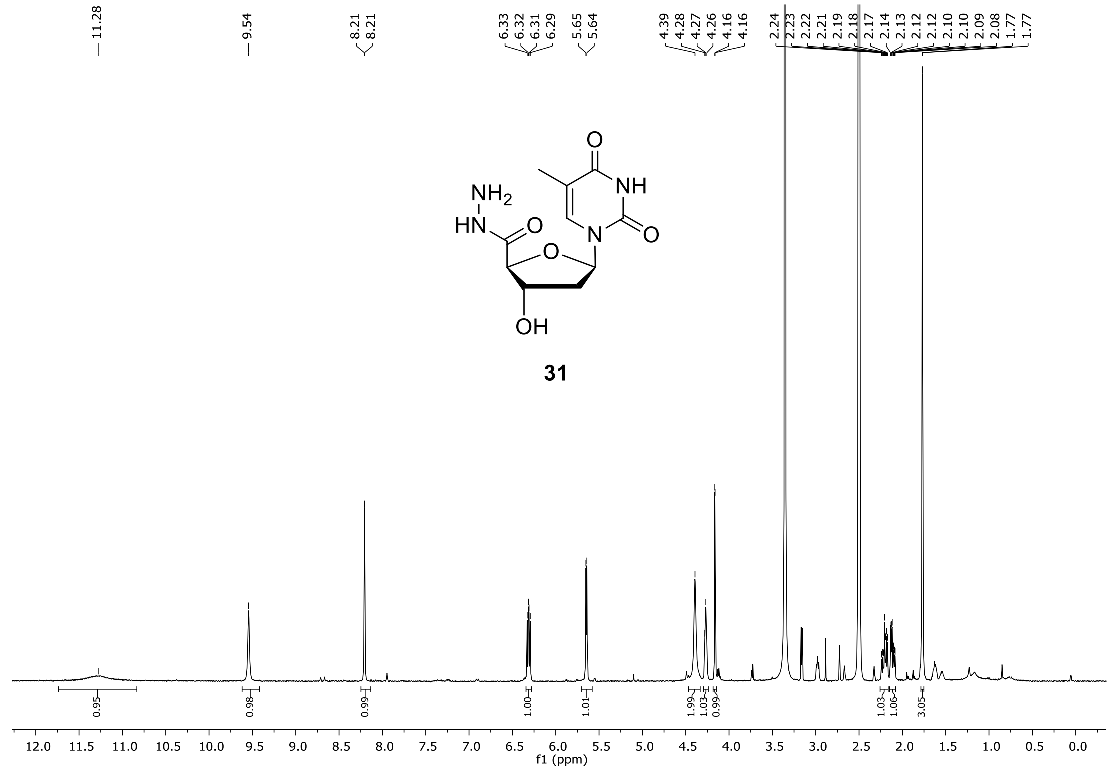


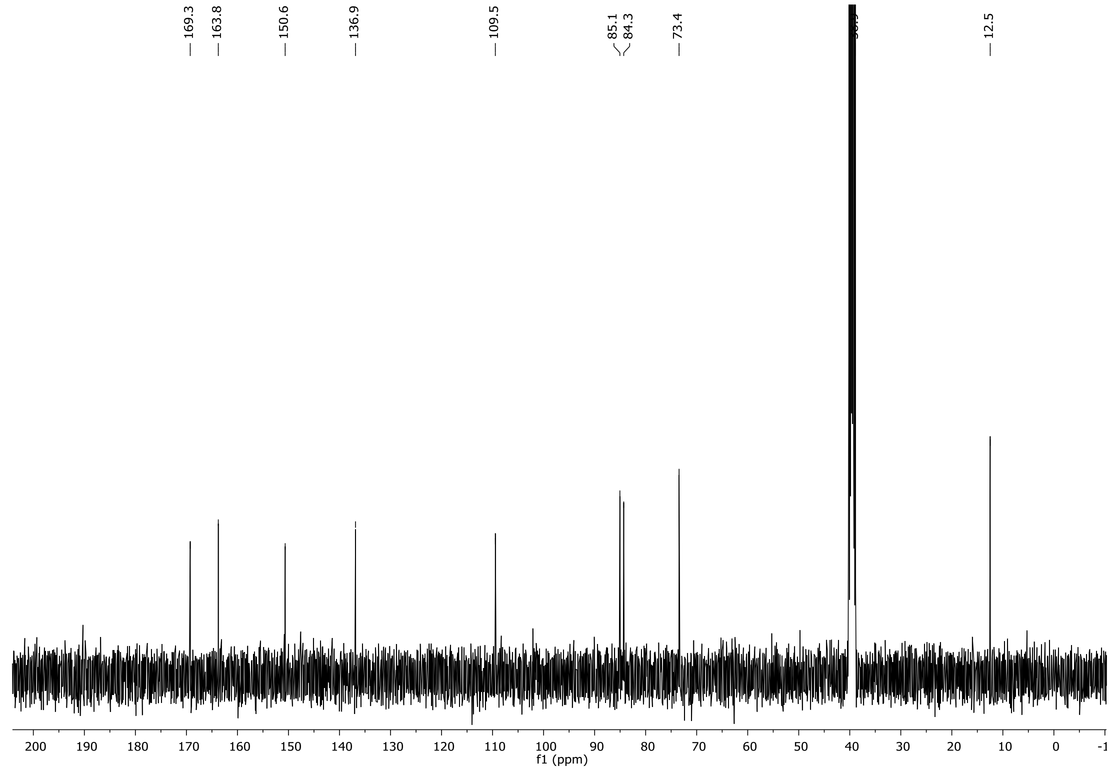

$^{1}H$ (600 MHz, DMSO-$d_6$) and $^{13}C$ (150 MHz, DMSO-$d_6$) NMR spectra of **34**

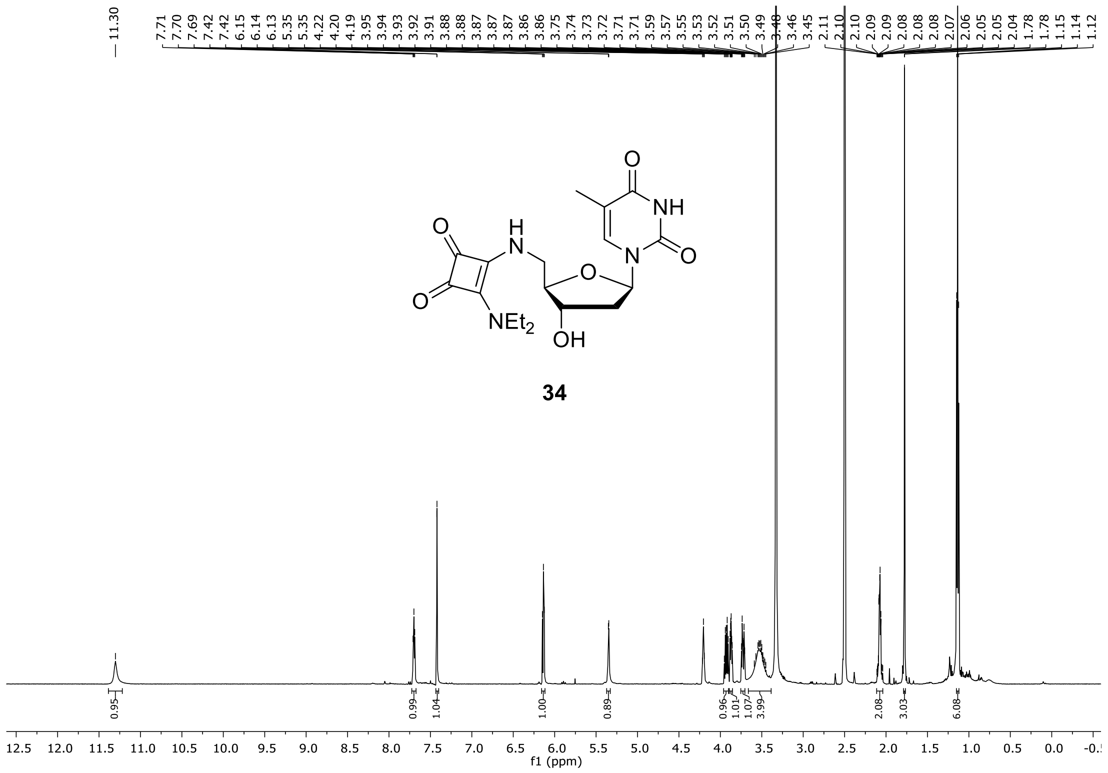


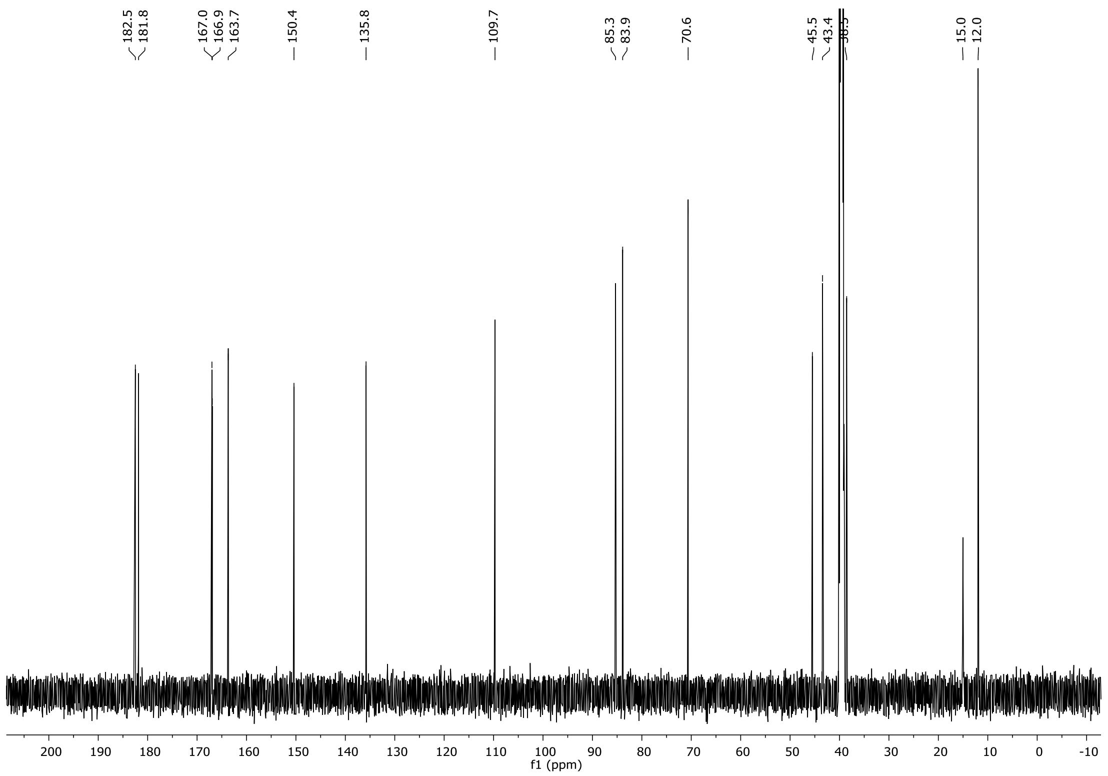

## PAMPA procedure and results

Permeability studies were carried out using the lipid−PAMPA method described by Merck Millipore.[14] A 96-well MultiScreen Filter Plate (Merck), with underdrain removed, was used as the donor plate, and a 96-well MultiScreen Transport Receiver Plate (Merck) as the acceptor plate. Carbamazepine and furosemide were used to confirm the integrity of the membrane. Solutions of hydroxamic acid **11** (500 µM), carbamazepine (500 µM) and furosemide (500 µM) in PBS pH 7.4 containing 5% dimethyl sulfoxide were prepared. PBS pH 7.4 containing 5% dimethyl sulfoxide (300 µL) was added to each well of the acceptor plate. A solution of lecithin in dodecane (5 µL, 1% w/v) was added to the filter within each donor well to form an artificial membrane. The drug solutions (150 µL) were immediately added to each well of the donor plate in quadruplicate. The donor plate was then placed into the acceptor plate and incubated at room temperature for 16 h. After the incubation, a sample of each donor well solution (100 µL) and of each acceptor well solution (250 µL) were transferred into a UV-star 96-well plate (Greiner Bio-one) and solutions of the equilibrium concentration were prepared by combining drug solution (150 µL) and PBS containing 5% dimethyl sulfoxide (300 µL) and samples of the equilibrium solution (250 µL) were also measured. Absorbance was measured from 250-500 nm using a SpectraMax Plus 384 plate reader (Molecular Devices). Concentrations were determined using a calibration curve. The effective permeability $P_e$ was calculated using the following equation:

$$\boldsymbol{P_e = -ln(1-r)\left(\frac{V_D V_A}{(V_D+V_A)At}\right)} \qquad \text{where} \qquad \boldsymbol{r = \frac{[drug]_{acceptor}}{[drug]_{equilibrium}}}$$

$V_D$ = volume of donor well, 0.15 cm$^3$ $\quad$ $V_A$ = volume of acceptor well, 0.30 cm$^3$

A = area of the filter, 0.3 cm$^2$ $\quad$ t = incubation time, 57 600 s

$P_e$ (Hydroxamic acid **11**) = 3.01727 x $10^{-8}$

log $P_e$ (Hydroxamic acid **11**) = -7.5

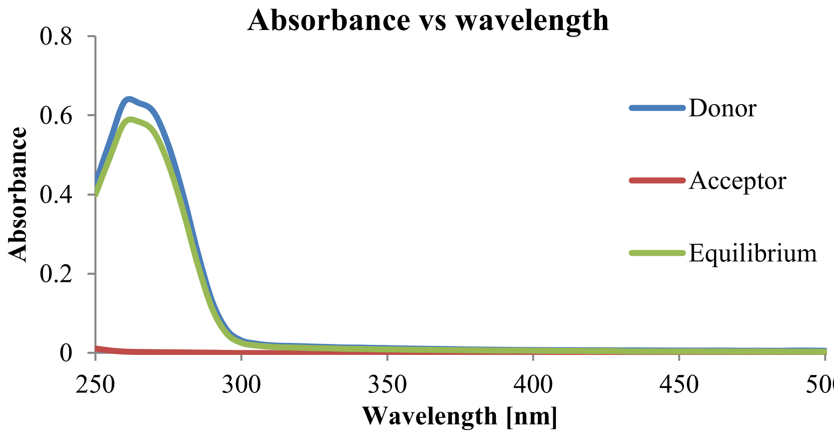


**Figure S1**: Absorption spectrum of donor wells (100 µL), acceptor wells (250 µL) and at equilibrium concentration (250 µL) containing hydroxamic acid **11.**

| | Donor | Acceptor | Equilibrium |
|---|---|---|---|
| Concentration [µM] | 488 | 1 | 182 |

**Table 1**: PAMPA of hydroxamic acid **11**. Concentrations of donor and acceptor compartments after 16 h incubation and equilibrium concentration.

## References


1. S. Peyrat and J. Xie, *Synthesis*, 2012, **44**, 1718.

2. E.-M. Dürr, W. Doherty, S. Y. Lee, A. H. El-Sagheer, A. Shivalingam, P. J. McHugh, T. Brown and J. F. McGouran, *ChemistrySelect*, 2018, **3**, 12824.

3. B. S. Ross, M. Han and V. T. Ravikumar, *Nucleosides, Nucleotides Nucleic Acids*, 2006, **25**, 765.

4. E. Werz, R. Viere, G. Gassmann, S. Korneev, E. Malecki and H. Rosemeyer, *Helv. Chim. Acta.*, 2013, **96**, 872.

5. S. Korneev and H. Rosemeyer, *Helv. Chim. Acta.*, 2013, **96**, 201.

6. K. A. Tallman and M. M. Greenberg, *J. Am. Chem. Soc.,* 2001, **123**, 5181.

7. A. Varizhuk, S. Kochetkova, N. Kolganova, E. Timofeev and V. Florentiev, *Nucleosides Nucleotides Nucleic Acids,* 2011, **30**, 31.

8. A. V. Kel′in, I. Zlatev, J. Harp, M. Jayaraman, A. Bisbe, J. O′Shea, N. Taneja, R. M. Manoharan, S. Khan, K. Charisse, M. A. Maier, M. Egli, K. G. Rajeev and M. Manoharam, *J. Org. Chem.,* 2016, **81**, 2261.

9. R. A. Aitken, J. M. Armstrong, M. J. Drysdale, F. C. Ross and B. M. Ryan, *J. Chem. Soc., Perkin Trans. 1,* 1999, 593.

10. M. Arujõe, A. Ploom, A. Mastitski and J. Järv, *Tetrahedron Lett.,* 2018, **59**, 2010.

11. S. K. V. Vernekar, L. Qiu, J. Zacharias, R. J. Geraghty and Z. Wang, *Med. Chem. Commun.*, 2014, **5**, 603.

12. K. Suthagar and A. J. Fairbanks, *Chem. Commun.*, 2017, **53**, 713.

13. D. Pan, J. Sun, H. Jin, Y. Li, L. Li, Y. Wu, L. Zhang and Z. Yang, *Chem. Commun.*, 2015, **51**, 469.

14. D. Schmidt and J. Lynch, Millipore Corporation Application Note, 2003, Lit. No. AN1728EN00
